# Laying the Foundation for Digital Material Design of Porous Transport Electrodes for PEM Water Electrolysis With Multiscale Tomography

**DOI:** 10.1002/smll.202513029

**Published:** 2026-02-18

**Authors:** Markus Bierling, David McLaughlin, Andreas Hutzler, Mingjian Wu, Darius Hoffmeister, Dennis Chalupczok, Erdmann Spiecker, Thomas Böhm, Simon Thiele

**Affiliations:** ^1^ Helmholtz‐Institute Erlangen‐Nürnberg for Renewable Energy (IET‐2) Forschungszentrum Jülich GmbH Erlangen Germany; ^2^ Department for Chemical and Biological Engineering Friedrich‐Alexander‐Universität Erlangen‐Nürnberg Erlangen Germany; ^3^ Institute of Micro‐ and Nanostructure Research, and Center for Nanoanalysis and Electron Microscopy (CENEM) Department of Materials Science & Engineering Friedrich‐Alexander‐Universität Erlangen‐Nürnberg Erlangen Germany

**Keywords:** catalyst layer, computed tomography, FIB‐SEM tomography, porous transport electrodes, proton exchange membrane water electrolysis, scale‐bridging tomography, transmission electron microscopy

## Abstract

We present the first multiscale identical location tomography of a porous transport electrode (PTE) of a proton exchange membrane water electrolyzer. The tomogram encompasses length scales between micrometer‐thick fibers to nanopores in the catalyst layer (CL) with nanometer‐thin binder coatings on the iridium‐oxide particles. It was recorded using micro‐computed tomography, focused ion beam scanning electron microscopy tomography, scanning transmission electron microscopy and spectroscopy, nitrogen adsorption, and supplemented by a modeling approach. The reconstruction of the PTE reveals a porosity of around 51% of the porous transport layer (PTL) and 61% of the CL. A thorough analysis of the CL allows a comparison with the pristine catalyst powder and a realistic prediction of the transport parameters by modeling the binder thickness to a mean of 7 to 10.5 nm. Further, the overall transport parameters of the PTE are determined. The PTL has a higher permeability in the through‐plane direction, whereas the CL shows isotropic transport properties. This study offers a comprehensive picture of the multiscale structure and properties of a PTE, which allows for a comparison with catalyst‐coated membranes and computer‐aided optimization of future PTEs.

## Graphical abstract

This paper presents the first multiscale identical location tomography of a porous transport electrode for PEM water electrolysis. Different tomography and modeling approaches determine the relevant structural properties ranging from micrometer‐thick fibers to nanometer‐thin binder films covering the catalyst particles. A correlation of the different tomograms allows the determination of the transport properties of the system.

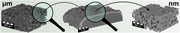

## 1 Introduction

Proton exchange membrane water electrolyzers (PEMWEs) are a promising technology to produce green hydrogen. PEMWEs show several benefits compared to other electrolysis technologies, including fast response times and a broad operational voltage range, which allows PEMWEs to be coupled excellently with volatile renewable energy sources [1]. However, it is necessary to reduce the production and operational costs of PEMWEs for their widespread application, e.g., by reduction or replacement of platinum group metals in the catalyst layers (CLs) or efficiency improvements with higher catalyst utilization and thinner membranes [2, 3].

Electrochemical reactions in a PEMWE take place at the membrane electrode assembly (MEA): the oxygen evolution reaction (OER) at the anode and the hydrogen evolution reaction (HER) at the cathode. The anode comprises a porous transport layer (PTL) and a CL that is usually iridium‐based. The cathode consists of a carbon‐based gas diffusion layer (GDL) with a microporous layer (MPL) and a platinum‐based catalyst. The solid polymer electrolyte membrane is sandwiched between the electrodes. The cathodic assembly is similar to the one found in PEM fuel cells and employs proven, stable, and well‐performing materials. The PTL is a porous substrate consisting of titanium fibers [4, 5] or sintered titanium powders [4, 6, 7] in order to withstand the high anodic potential and low pH values at elevated temperatures. The catalyst ink can be either processed on a PTL or GDL as a catalyst‐coated substrate (CCS) or onto the membrane as a catalyst‐coated membrane (CCM) [8]. CCS on the cathode side is referred to as a gas diffusion electrode (GDE), and the anodic counterpart is called a porous transport electrode (PTE). An illustration can be found in Figure S1. The decal transfer method is the current state‐of‐the‐art manufacturing technique for CCMs, but it is not applicable to CCSs. The CL of GDEs and PTEs can be coated onto the substrate using different methods [9, 10, 11, 12], such as spray coating or electrochemical deposition [13, 14, 15].

Leonard et al. and Kulkarni et al. measured significantly higher kinetic overpotentials for PTE configurations in comparison with CCM systems with the same PTLs [16, 17]. They concluded that the impaired kinetics of PTEs originate from proton transport limitations in the CL due to poor ionic conductivity. On the other hand, PTEs also offer advantages, like better electronic conductivity and a simplified manufacturing technique by directly coating the catalyst ink on the substrate, which is less restrictive toward suitable membrane and electrode binder materials than the decal transfer employed for CCM production [18, 19]. Despite the generally impaired kinetics of PTE‐based systems, Bühler et al. showed that optimized PTE configurations can achieve similar performances compared to CCMs [10]. Weber et al. [20] compared the electrochemical performance and structure of CCMs and PTEs using PTLs and graded PTLs with an MPL, called microporous electrodes (MPEs). Using an MPL, they could successfully reduce the catalyst loading from 2.5 to 0.5 mg_Ir_ cm^−2^ without a significant loss in performance due to a more homogeneous catalyst distribution. However, the CCM configuration with the same loading outperformed the MPE. They assumed a lower catalyst utilization in the PTE and MPE configurations due to the catalyst layer penetration into the deep pore space during deposition. Still, the MPE setup reduces the amount of inactive catalyst in the PTL due to reduced surface roughness and pore sizes at the surface compared to a PTE without MPL.

CCM approaches are currently considered state of the art. Yet, PTEs have also found industrial applications. By developing a new sputtering technology, Toshiba Corporation was capable of manufacturing PTEs on a large scale [21, 22]. This technique was recently implemented by Lee et al., who reported binder‐free PTEs with a catalyst loading of less than 0.1 mg cm^−2^ showing good performance, durability, and potential for recycling [23]. The same group [24] was able to further improve binder‐free PTEs by modifying the surface of a PTL with nanochannels via femtosecond laser ablation. This nanochannel structure improved the mass transport properties by supplying additional pathways for oxygen and water transport, and it increased the electrochemically active surface area due to the increased surface area of the nanochannel structure.

**FIGURE 1:**
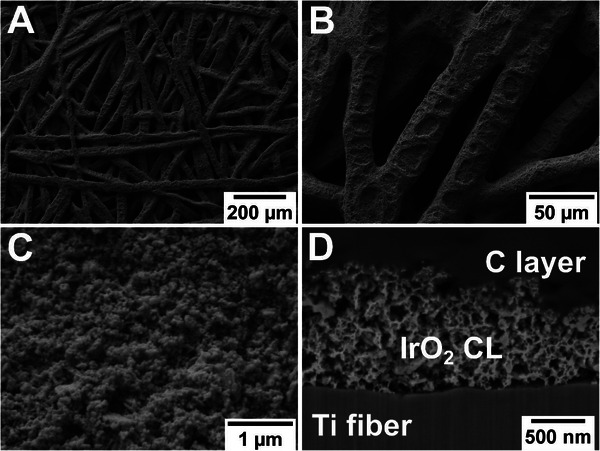
Overview of an anodic PTE of a PEMWE. In the surface images (A–C), the PTE is depicted with different magnifications: (A) Overview of IrO_2_‐coated titanium fibers, (B) closer view of the catalyst pattern on the fibers, and (C) surface morphology of the CL on a single fiber. Additionally, a FIB‐SEM cross‐section of the CL on a fiber is shown in (D), with the titanium fiber in the lower part, the porous CL in the center, and the protective carbon layer from FIB processing in the upper part of the micrograph.

Besides these advances in PTE development and the possible benefits of the concept, there is still a lack of understanding of the interplay between OER kinetics in PTE‐based MEAs and the morphology of the electrodes. This transport‐activity nexus can only be fully depicted when the structure and the electrochemical data are analyzed together [25]. A closer look at the morphology shows that a PTE is a multiscale system (see Figure 1; Figure S2). The porous network of the titanium PTL is on a micrometer length scale (Figure 1A,B). The thickness of the manufactured CL is around 1 µm, but the grain and pore sizes of the CL are in the range of several dozen to hundreds of nm (Figure 1C,D). Imaging methods require a compromise between resolution and field of view, which means that a single approach cannot resolve the whole structure at the desired resolution. Therefore, multimodal approaches are needed, like the ones that have been successfully employed in battery [26], PEM fuel cell [27, 28], alkaline water electrolysis [29], and CO_2_ electrolysis [30] research. With these approaches, the transport phenomena in a system can be correlated to its electrochemical performance, which is needed to implement digital material design.

The structural analysis of the titanium PTL was the focus of numerous studies [31, 32]. Especially, the structure‐performance relationship and the interfacial properties between PTL and CCM have been of particular interest in the last few years [5, 6, 7, 25, 31, 33, 34, 35, 36, 37, 38, 39]. Hegge et al. [40] presented the first reconstruction of a PEMWE anode CL, a commercially available FuelCellsEtc EZ CCM with a loading of 3 mg cm^−2^ IrRuO_x_, via a focused ion beam scanning electron microscopy tomography (FIB‐SEMt). Angelis et al. [41] presented X‐ray ptychographic tomography as a new characterization method for the structural analysis of the CL of PEMWE anodes consisting of IrO_2_ supported on TiO_2_. Imaging under cryogenic temperature to minimize beam damage allowed them to image agglomerations of the ionomer binder in the CL. Additionally, they were able to distinguish between IrO_2_, TiO_2_ support, binder, and pore. The same group imaged and analyzed a CL consisting of IrO_2_ supported on TiO_2_ under dry and wet conditions using ptychographic X‐ray laminography, revealing a decrease in electric conductivity under wet conditions [20]. Lee et al. [42] also used full‐field transmission X‐ray microscopy at a synchrotron facility to image iridium‐based CLs and determine effective transport parameters. The same group used transmission X‐ray microscopy tomography data sets to stochastically generate CLs and calculate their transport properties [43]. Ferner et al. [44] compared the morphology of iridium oxide CLs fabricated via spray‐coating and blade‐coating using nano‐CT. Furthermore, using FIB‐SEMt, they found a lower porosity and smaller pore sizes of the CL, caused by local compression due to the titanium PTL.

Several studies show the morphology of PTEs by scanning electron microscopy (SEM) [10, 45, 46]. The first determinations of a qualitative catalyst distribution were performed by Leonard et al. [16] and Kulkarni et al. [17] They performed *operando* imaging of a PEMWE with PTE configuration via X‐ray micro‐computed tomography (micro‐CT). Their results revealed that the CL adheres to the shape of the titanium substrate. In a previous study [47], we were able to determine the catalyst distribution of a PTE quantitatively. Most of the catalyst was located within the first 100 µm below the surface of the PTL. Weber et al. [20] compared the difference in performance and structure of a PTE with and without an MPL. They characterized the catalyst distribution of both configurations via X‐ray micro‐CT and determined a 1.5 times higher catalyst accessibility and a more homogeneous catalyst distribution when using an MPL.

However, to the best of our knowledge, no literature exists that takes the multiscale structure of a PTE into account. The above‐mentioned studies either focused on the PTL or on the catalyst distribution of the PTE [16, 17, 20, 47], but not on the application‐relevant full picture of both structures. In particular, no study exists that analyzes the CL of a PTE, which forms during the coating process on the rough, porous substrate of a PTL. Only CLs of CCMs [40, 41, 42, 44] and CLs on flat substrates [20] were investigated to date.

Hence, we aim to provide a full picture of a PTE by combining high‐resolution tomography of the CL in conjunction with a tomogram that covers the full PTL thickness. A spray‐coated iridium oxide CL on a titanium fiber PTL was investigated by several FIB‐SEMts and X‐ray computed tomography on the same sample to depict the complete structure of this PTE at all relevant length scales. This approach was complemented by transmission electron microscopy (TEM) imaging and spectroscopy, thermogravimetric analysis, and modeling to gain further insights into the CL's binder distribution. The structural and transport parameters obtained and calculated from this multiscale tomogram will help to understand the transport activity nexus of anodic PTEs better, which ultimately helps optimize the catalyst utilization and thus improve the overall performance. Furthermore, the multiscale tomogram, binder modeling, and catalyst deposition process modeling [47] can serve as a baseline and starting point for future digital material design studies in PEMWE research to elucidate the influence of these structures and improve their performance and durability using simulations. Such digital material design studies will be essential to keep pace with the rapid development of new components, such as catalysts or PTLs.

## 2 Results and Discussion

A full understanding of the properties of a PTE requires knowledge of its morphology on all relevant scales. First, the structure of the PTL and CL was analyzed separately via µCT and FIB‐SEMt. As the CL is coated onto the PTL, the two structures are interconnected and cannot be considered independently. Therefore, the components were put into relation via a FIB‐SEMt of a local CL‐fiber interface as well as catalyst distribution information based on the µCT data of the PTE. Thus, an identical‐location multiscale tomogram of the anodic PTE was obtained (Figure S3). Additionally, the binder of the CL was modeled into the structure, based on TGA data and 2D scanning transmission electron microscopy with energy dispersive X‐ray spectroscopy (STEM‐EDXS) and with electron energy loss spectroscopy (STEM‐EELS). Finally, the transport parameters of the PTL, CL, and the PTE were calculated, laying the foundation for future digital material design and optimization.

### 2.1 Individual Morphology of PTL and CL

**FIGURE 2:**
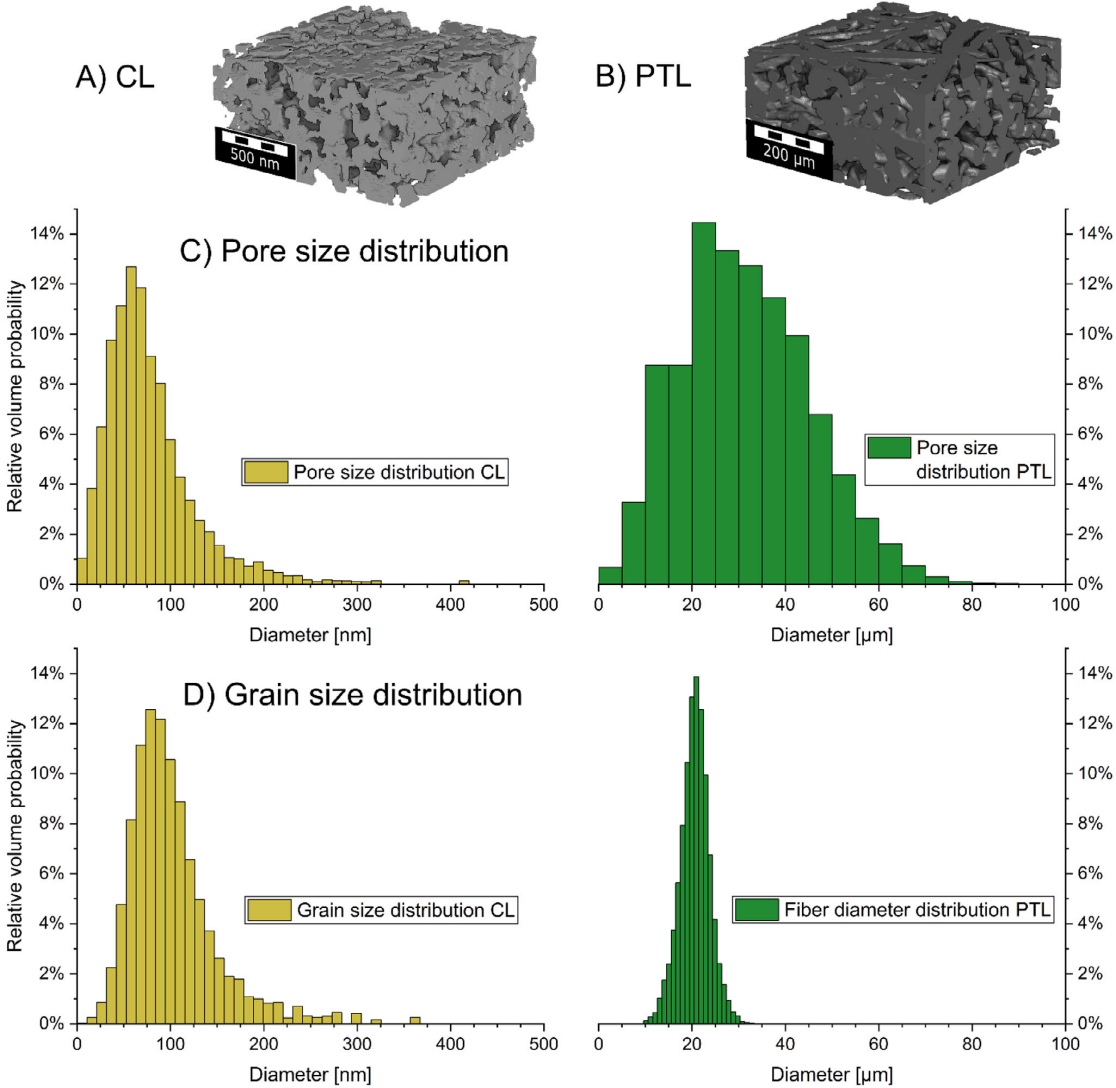
Illustration of (A) CL and (B) PTL [47]. Respective pore size and grain size distribution are visualized in (C) and (D). Pore size and fiber diameter distribution of the PTL are taken from our previous work [47].

While the structure must be considered holistically, it behooves us to begin the analysis with the analysis of the individual components. The structure of the anode CL and the titanium fiber PTL is highlighted in Figure 2A,B. The PTL structure is reproduced from our previous study [47] based on µCT. A porosity of 50.84%, a mean pore diameter of 30.20 µm (Figure 2C), a mean fiber diameter of 20.59 µm (Figure 2D), and a strong horizontal fiber orientation were determined in the segmented tomogram.

The location of the reconstructed CL volume was chosen on top of an upper horizontal fiber (Figure S3) because the horizontal plane of the uppermost catalyst‐coated fibers presumably contributes most to the electrochemical reaction due to their direct contact with the membrane. Second, the CL thickness is expected to be larger on the top of the fiber than at the fiber edges, leading to a larger and, therefore, more representative imaged volume. Finally, the penetration depth of the Gallium ions of the FIB is limited. Therefore, the top layer is more easily accessible than the fiber edges.

Three individual subvolumes of the high‐resolution FIB‐SEMt were analyzed to characterize the CL (Figure S4). The segmented CL has a porosity of 60.88% ± 0.41%, a mean grain diameter of 102.4 ± 48 nm, and a mean pore diameter of 78.6 ± 49.3 nm (Figure 2C,D). The large standard deviations originate from the broad grain (GSD) and pore size distribution (PSD), which include a wide tail. Most grains and pores have a diameter below 200 nm, but also some larger grains and pores up to 400–500 nm occur (Figure S5). The distributions between the three investigated subvolumes are similar (Figure S6), including similar mean grain diameters (102.4 ± 1.9 nm) and mean pore diameters (78.6 ± 3.0 nm) between the subvolumes. The similar PSD and GSD of the subvolumes (Figure S7), together with the representative volume analysis (Figure S8), proved the statistical significance of the volumes. The PSD of the CL follows a log‐normal distribution (Figure S7), which is in line with a report by Ferner et al. [44] who analyzed the morphology of the same iridium oxide catalyst from Alfa Aesar. They investigated a blade‐coated layer, which had a porosity of around 50% and a mean pore diameter of 123.3 ± 78.6 nm. The deviation can be due to differences in the ink formulation, as blade‐coating is performed with inks of higher viscosity (different solvent and higher solid volume content) compared with the herein performed spray‐coating. Alternatively, the difference can arise from the different fabrication approach (doctor‐bladed CCM with decal transfer from PTFE substrate compared with PTE).

Other tomograms of anode CLs in PEMWEs showed higher mean grain and pore diameters but porosities in a similar range. For example, Hegge et al. [40] determined a mean grain diameter of 500 nm, a mean pore diameter of 530 nm, and a total porosity of 55% for a commercial IrRuOx CL. Angelis et al. [41] and Weber et al. [20] analyzed core–shell CLs manufactured by spray‐coating. Their structures revealed grains and pores in a similar dimension to Hegge et al. [40] They found lower porosities of 42% [41] and 45% [20], which can be explained by the ionomer binder that they identified in the CL, which was not visible in the data by Hegge et al. [40] Lee et al. [42] examined another iridium‐based CL, with a mean pore diameter of 203 nm, a mean grain diameter of 247 nm, and a porosity of 39%.

Next, the surface area of the CL was approximated based on the tomogram. The specific surface area of the segmented CL, *S*_FIB‐SEMt_, was calculated as *S*_FIB‐SEMt_ = 2.64 ± 0.03 m^2^ m^−3^. A conversion to the mass‐referenced surface area *S’*_FIB‐SEMt_ resulted in 10.09 ± 2.06 m^2^ g^−1^. The pristine catalyst powder was also measured by N_2_ physisorption, which resulted in *S*_BET_ = 28.70 ± 1.01 m^2^ g^−1^ (see Supporting Information chapter “BET analysis” and Figure S9). Notably, literature provides a wide range of specific surface areas for this material (between 25 and 57 m^2^ g^−1^ [48, 49, 50]), which covers the value obtained by N_2_ absorption.

The difference between the specific surface area of the pure catalyst determined via N_2_ physisorption and the specific surface area of the CL can have several reasons. First, the FIB‐SEMt has a limited resolution and, therefore, cannot resolve all features of the CL despite the employed staggered‐grid compensation. Furthermore, the CL consists of IrO_x_‐agglomerates and a binder phase, which lowers the surface area compared to a pure catalyst powder when neglecting a possible loss of binder due to beam damage in FIB‐SEMt. A discrepancy between the surface area of the CL determined via FIB‐SEMt and the predicted surface area via BET was also found in the literature [40]. The specific surface area of the reconstruction can be considered as a lower limit, and the specific surface area of the BET measurement as an upper limit of the real electrochemically active surface area (ECSA). Deeper insights into the exact surface area and possible unresolved features of the CL could be obtained by cryogenic transmission electron tomography, as performed in PEMFC [51], due to the higher resolution at the nanometer scale. This is particularly important when performing quantitative simulations in the kinetic region, as the ECSA, along with the catalyst's activity, governs the electrochemical performance in this region [52]. Therefore, the presence of unresolved nanoscale features hinders the use of these structures for accurately simulating kinetics and activation losses. However, the uncertainty in the ECSA is expected to have a negligible influence on subsequent transport simulations in the ohmic and mass transport regimes since FIB‐SEMt provides a sufficient resolution to exclude the presence of unresolved pores or significantly altered grain or pore sizes between the sample and its 3D reconstruction.

**FIGURE 3:**
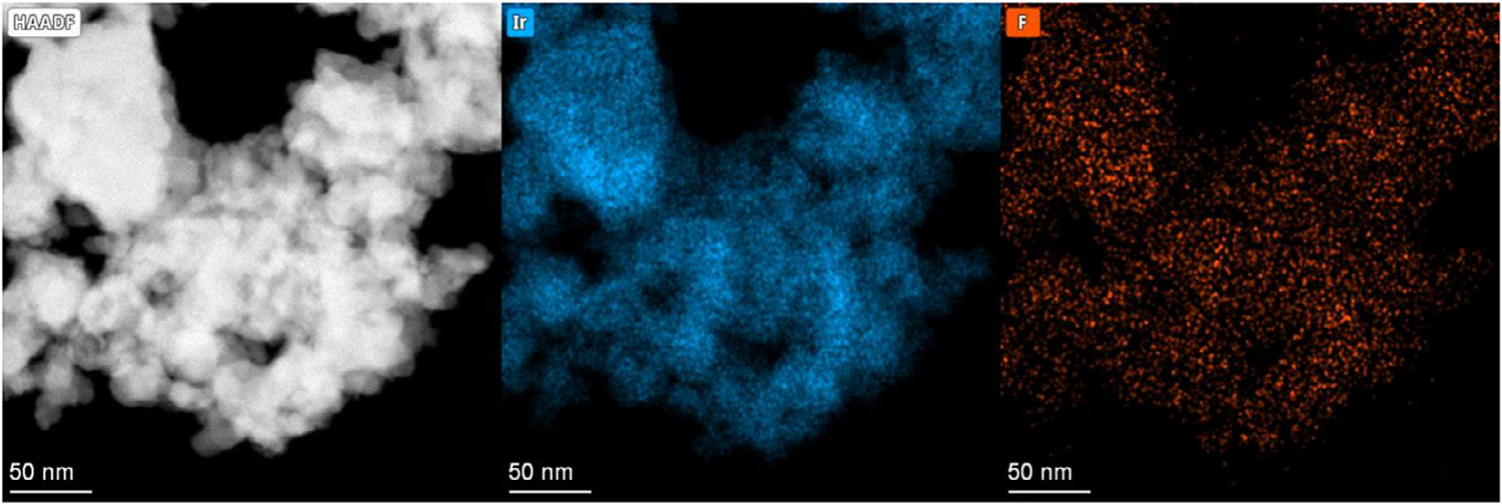
HAADF‐STEM and STEM‐EDX spectrum images at 80 kV of a small fraction of the CL. The catalyst (Ir) and ionomer (F) signals overlap, indicating a high degree of coverage of the catalyst particles with the binder.

The mean particle diameter of the pristine catalyst particles was estimated from the BET data under the assumption of homogeneous spherical particles. The calculated diameter was around 18 nm (Supporting Information chapter “BET analysis”). This value lies within a reasonable range when looking at high‐angle annular dark field (HAADF) STEM images of the catalyst particles, which also reveals the occurrence of aggregates (Figure 3). The volume ratio between the average grain size from the tomogram and the mean particle diameter from BET shows that an average grain consists of 184 single catalyst particles when neglecting the binder. HAADF‐STEM images of the pristine catalyst powder suggest that a fraction of the catalyst particles is present in the form of larger aggregates (Figure S10). It remains elusive whether the homogenization via ultrasonication is unable to separate the aggregates or if they form during drying.

### 2.2 Multiscale Character of the PTE

The electrode is produced on a highly porous substrate, which results in an anisotropic structure. Thus, we investigate the multiscale character of the PTE by analyzing the CL/PTL interface and the catalyst distribution within the PTE to enable a complete picture of the structure of the full PTE. Complementary, STEM‐EDXS and STEM‐EELS measurements provide insights into binder distribution.

#### 2.2.1 Catalyst‐Coated Titanium Fibers

Our previous analysis of the overall catalyst distribution within the PTE revealed that the through‐plane CL distribution followed a log‐normal shape with over 90% of the catalyst within the first 100 µm [47] (Figure S11D). However, this analysis is insufficient to determine the morphology of the catalyst‐coated titanium fibers, especially the interface between catalyst and titanium fiber, the local CL thickness, and its surface roughness.

**FIGURE 4:**
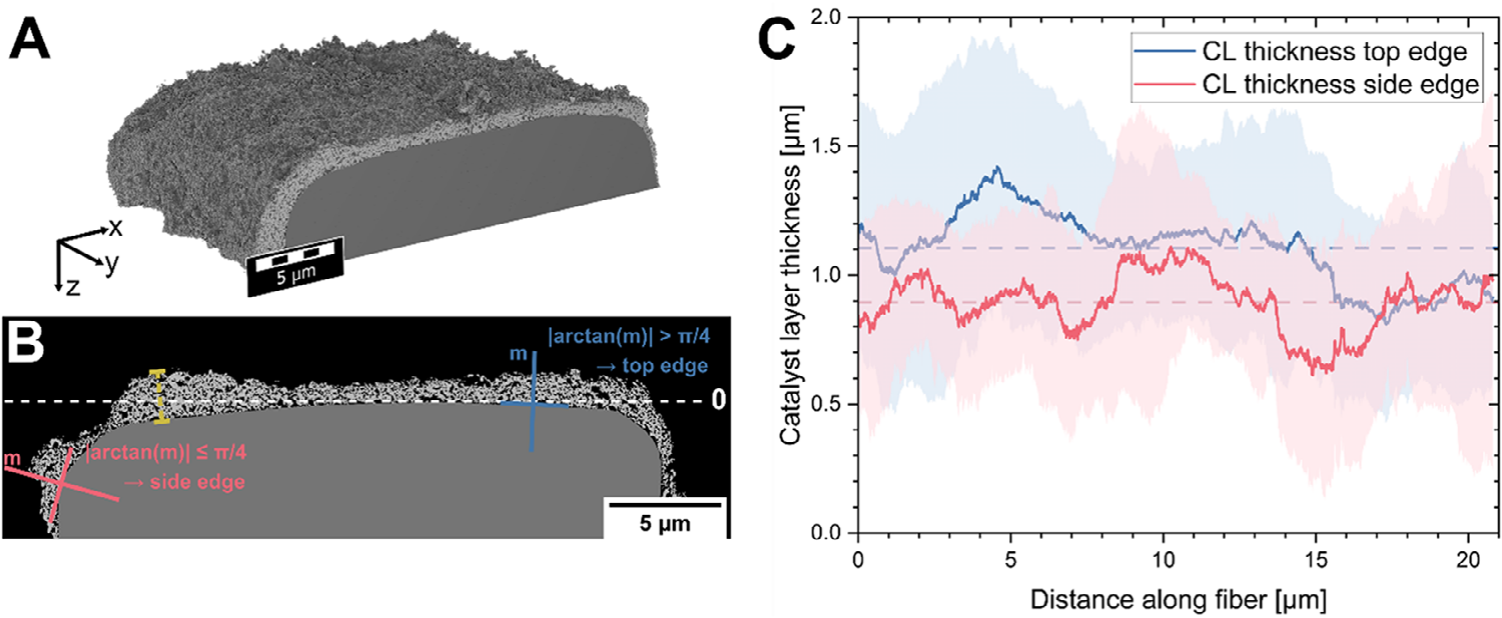
Orientation‐specific evaluation of CL thickness. (A) Overview of the tomogram of the catalyst‐coated fiber. (B) Separation of CL thickness into the side edge and the top edge. As a criterion, the slope of the normal, here the direction of the CL in each point, was selected. Slope angles between 45 and 135° (“|arctan(m)| > π/4”) were attributed to the top edge, and the rest was attributed to the side edge. See methods for a detailed explanation. (C) CL thickness of the top and side edges with standard deviation along the catalyst‐coated fiber.

Therefore, a FIB‐SEMt of the upper half of a catalyst‐coated fiber was performed for a detailed analysis of the CL morphology in dependence on the fibrous substrate (Figure 4A). In contrast to ionomer‐free PTEs produced via sputtering [23], spray‐coated PTEs are expected to have a rougher CL due to the porous structure formed during the ink‐drying process. Notably, the catalyst morphology can differ depending on the fiber location (e.g., free‐standing fiber or overlapping fibers), the distance between fibers (e.g., ink droplets trapped between two adjacent fibers), the through‐plane position (e.g., top fiber or deeper fiber), or the catalyst ink droplet size. We chose a free‐standing catalyst‐coated fiber at the surface of the PTE without visible drying artifacts (Figure S12) for two reasons. First, the upper fibers will contribute most to the electrochemical water splitting because of their direct contact with the membrane. Second, a free‐standing fiber without overlapping areas with adjacent fibers allows the best differentiation between horizontal and vertical coating surfaces. Notably, the reconstructed 20 µm of fiber length showed considerable inhomogeneity in CL thickness and titanium fiber structure (Figure S13), although this fiber section is oriented parallel to the PTE surface and, therefore, has ideal conditions for being coated (Figure S12).

A considerable deviation in the CL thickness is already visible in a single reconstructed slice of the tomogram (Figure 4B; Figure S14). Figure 4C shows the average CL thickness along the investigated fiber, which shows that the average CL thickness on the top edge (1.104 µm ± 0.466 µm) is greater than the thickness on the side edge (0.899 µm ± 0.417 µm). Notably, the thickness is very inhomogeneous, as indicated by the large standard deviations for both values. Further, the top edge CL thickness decreases along the imaged fiber volume between 15 and 20 µm, which is correlated with a steep increase in the relative fiber height (see Figure S15). The side edge CL thickness fluctuates constantly, but on a lower baseline than the top edge CL. The here‐determined CL thicknesses are in good agreement with the modeling assumptions of 1.3 µm for this PTE from our previous study [47]. The difference can be explained by the possible clogging of catalyst ink at some porous bottleneck throats between two fibers that consume additional ink and result in larger local CL thicknesses that are not represented in the tomogram (Figure 4C). Notably, the side edges of this fiber maintained a CL thickness of more than 0.7 µm at a depth of 6 µm, with strong deviations between the left and right side (Figure S16).

Finally, the roughness of the CL was also determined (Supporting Information chapter “Surface roughness catalyst layer”). A high surface roughness was suggested by the large standard deviation of the CL thickness (Figure 4C) and is confirmed by the arithmetic mean surface roughness $R_{a}^{\mathrm{CL}}$= 0.362 µm at an average thickness of 1.047 µm (Table S1).

Together with the findings of our previous paper [47], these results regarding topography and CL thickness provide a comprehensive overview of morphology on a microscopic to mesoscopic scale. The topography of the PTL defines the catalyst utilization and is necessary for modeling the PTE‐membrane interface, as done in CCM modeling for the PTL‐CL interface [39, 53]. The CL thickness, topography, and coverage determine the local CL properties and transport parameters.

#### 2.2.2 Catalyst and Binder in the CL

STEM‐EDXS analyses (Figure 3; Figure S17) show that the particles formed aggregates in the CL, which was also observed for pristine catalyst powder without binder (Figure S10). Agglomerated particles were also observed in literature for unsupported Ir [54]. An EDXS (Figure 3; Figure S17) analysis of the elemental composition indicated that the binder was homogeneously distributed in the CL. However, this result must be interpreted carefully due to a low signal‐to‐noise ratio. Low excitation energies for fluorine atoms, a low binder content in the CL (∼10 wt.%), and beam damage due to the extreme beam‐sensitive nature of Nafion [19, 55] complicate the binder detection.

Additionally, careful electron dose‐controlled experiments with electron energy loss spectroscopy (EELS) were performed. This technique has already been applied in PEMFC to distinguish the ionomer phase from the carbon support using the carbon core‐loss signal produced by STEM‐EELS [57]. However, the occurrence of beam damage limited this analysis to only qualitatively verifying the binder's presence in the CL (Figure S18 and Supporting Information chapter “STEM‐EELS core‐loss analysis”).

**FIGURE 5:**
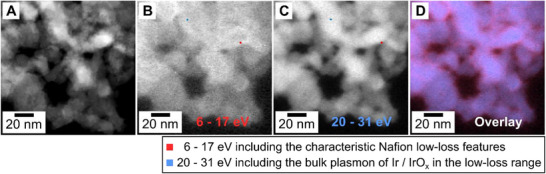
STEM‐ADF and low‐loss EELS analysis of a small fraction of the CL. (A) STEM‐ADF image of the CL with a resolution of 1.1 nm per pixel. (B) and (C) Low‐loss EELS maps after deconvolution and zero‐loss removal using energy windows of 6–17 eV (characteristic Nafion low‐loss features [56]) and of 20–31 eV (the bulk plasmon of Ir). The homogeneous intensity distribution of the 6–17 eV indicates a core–shell structure. EELS spectra of the red (at the particle center) and blue (at the particle edge) markers are shown in Figure S19. (D) Overlay of both low‐loss EELS maps highlights the binder layer's presumed core–shell characteristic.

Because unambiguous elemental mapping of the Nafion binder (using core‐loss EELS of F‐K at ∼685 eV) was precluded by severe beam sensitivity (Figure S18 and Supporting Information chapter “STEM‐EELS core‐loss analysis”), we tried to localize the ionomer using low‐loss STEM‐EELS (Figure 5). After Fourier‐log deconvolution and zero‐loss removal of the spectrum image, energy‐selective maps integrated over 6–17 eV, which is a window that encompasses the characteristic Nafion low‐loss features (∼7–9 eV with satellites to ∼17 eV [56]), revealed a “shell‐like” bright annular signal (Figure 5B) encasing the ADF‐identified Ir/IrO_x_ clusters of nanoparticles (Figure 5A). The shell appears to have an average wrapper thickness of several nm, consistent with a Nafion shell/binder layer. A complementary map integrated over 20–31 eV, where the response is dominated by the Ir/IrO_x_ bulk plasmon (peaked at ∼26 eV), reproduces the cores of clusters of nanoparticles (Figure 5C). The shell contrast remains robust across fields of view and modest variations in integration limits, indicating a physical polymer wrapper rather than other possible reasons (Figure 5D). Further statistical analysis and studies on beam damage are necessary to make more precise statements about binder thickness and the degree of binder coverage.

Furthermore, a modeling approach was used to estimate the binder distribution. Based on previous studies on the binder distribution in PEMFCs [51, 58, 59, 60] and the above performed EELS analysis, we expect several nanometer‐thick binder films to build a non‐homogeneous network around the catalyst structure.

FIB‐SEMt cannot distinguish between solid particles and nanometer‐thin volumes of binder [30]. The high energy input of the accelerated Gallium ions can decompose or even remove the binder [61]. Furthermore, the different contrast of Nafion ionomer and iridium oxide leads to different grayscale levels during image acquisition. Therefore, binder areas at the outer solid edge can be mistakenly segmented as pore space. Consequently, two extreme cases of binder distribution were considered by modeling: either all binder was segmented as part of the CL grains, or no binder was segmented because the binder was destroyed due to beam damage.

**FIGURE 6:**
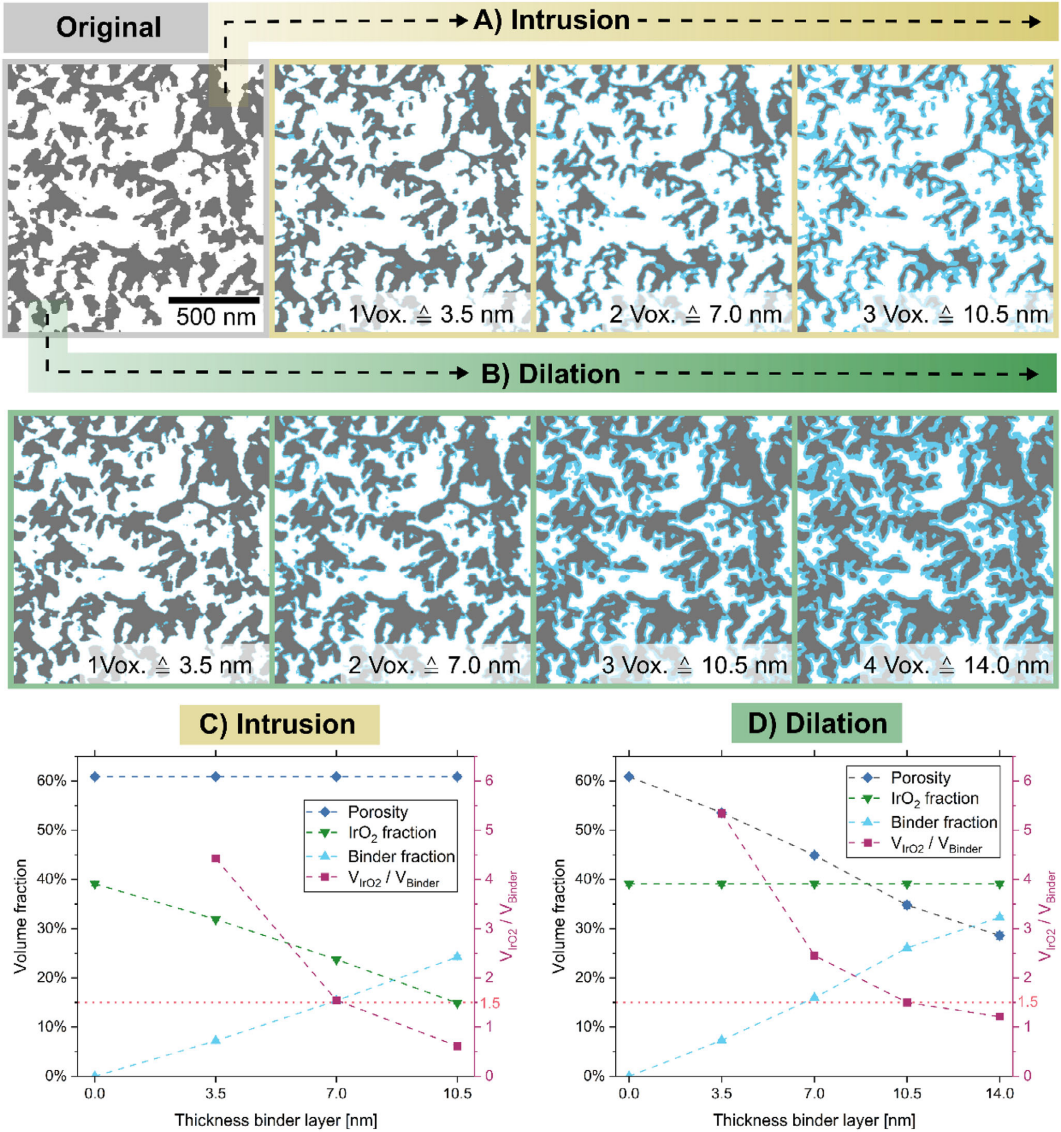
Binder modeling via intrusion (yellow) and dilation (green). Illustration of the morphology changes due to the increase in binder volume fraction via (A) intrusion (yellow) and (B) dilation (green) of agglomerates. The changes in pore volume fraction, IrO_2_, and binder phase due to intrusion and dilation are depicted in (C) and (D). The calculated volume ratio between iridium oxide and the Nafion ionomer of 1.5 is marked with a red line.

For scenario 1 (all binder segmented), the outer detected grain fraction was iteratively eroded and afterward assigned as the binder (Figure 6A). Therefore, the IrO_2_ volume fraction continuously decreases while the binder content gains the eroded amount (Figure 6C). The porosity of the structure remains constant during this reassigning process. For scenario 2 (no binder segmented), the grains were iteratively dilated and assigned as binder (Figure 6B). The constant IrO_2_ volume content, in combination with an increasing grain volume fraction, leads to a decreasing porosity (Figure 6D). Additionally, a filling of small pores is observed with increasing binder thickness.

The binder modeling approach via intrusion and dilation is a simplification that is based on the morphology of the CL and EELS observations. The actual binder distribution is assumed to be a combination of thermodynamic factors, such as ink composition [62, 63] and interactions between catalyst particles, binder, and solvents [64, 65], as well as process‐controlled factors, such as the coating method [66, 67] or drying temperature [66, 68].

Knowing the *V*_IrO2_/*V*_Binder_ fraction of around 1.5 (see Equation (4)), the mean binder coverage thickness *t̅*_binder_ of both cases can be estimated. The scenario with the segmented binder suggests that the mean thickness is around 7.0 nm, which equals a binder volume fraction of 15.4% of the CL (Figure 6C). The scenario with no segmented binder results in a mean thickness of around 10.5 nm, which equals a total binder volume fraction of 26.1% of the CL (Figure 6D). This modeling leads to an expected window of the mean binder thickness: 7.0 nm ≤ *t̅*_binder_ ≤ 10.5 nm. As a general remark, all binder volume fractions here are considered under dry conditions. Under operating conditions of 80 °C and with the supply of liquid water, the swelling of the ionomer leads to a binder volume fraction increase by a factor of up to 1.8 (1.8 [52, 69], 1.57 ± 0.31 [20]). Hence, the binder volume fraction would increase to 27.7% (scenario 1) or up to 46.9% (scenario 2), followed by a decrease in porosity down to 49.6% (scenario 1) or 13.9% (scenario 2) in the wet state (see Figure S20).

This thin mean binder thickness is consistent with the results obtained via STEM‐EDXS and STEM‐EELS. Both methods indicated a thin film covering a high degree of the catalyst particles with binder (Figures 3 and 5).

The modeling approach of adding binder via intrusion and dilating has also been performed for CCM anode CLs in PEMWE [42, 43]. Another approach was conducted by Hegge et al. [40], who modeled the binder on an IrRuO_x_ structure with median agglomerate diameters of 500 nm via an “add binder” function that simulates binder like a wetting fluid and fills smaller pores first. However, they did not consider the original binder volume fraction of their CL. Angelis et al. [41] and Weber et al. [20] both imaged all phases of a core–shell anode CL and were able to identify the binder distribution. The binder distributions mainly showed binder thicknesses in the range of several hundred nm due to the large catalyst grains (mean grain diameter > 300 nm). Notably, binder thicknesses below 20 nm could not be resolved due to the voxel size. These modeling and imaging studies had much bigger catalyst agglomerates and coarser structures than the herein analyzed system. Therefore, it is difficult to compare these configurations. Furthermore, none of these studies correlated the added binder with the real binder volume fraction determined via TGA.

Because of the lack of comparable literature in PEMWE, results from fuel cell research [51, 58, 59, 60] are taken into account. Pt/C CLs share similar feature sizes with the IrO_x_ CL, with pore diameters of 100 nm or less [70, 71]. Depending on the I/C ratio, average binder layer thicknesses ranged from 7.2 [58] to 9.2 nm [51] with a broad binder thickness distribution. The coverage ratio was up to 80% [51, 58]. Considering these results from PEMFC CLs, the binder thickness of 7.0 nm ≤ *t̅*_binder_ ≤ 10.5 nm is in a reasonable range. A high degree of coverage, but with an inhomogeneous binder thickness, can be expected. Therefore, our model is a good approximation, and the two extreme cases can serve as a baseline for the following transport parameter simulations. It shall be noted that any subsequent analysis can suffer from a bias as the binder thickness increases under operation of the MEA due to swelling of the ionomer [20]. Additionally, the density of thin films of Nafion is lower than that of bulk material [72], and therefore, the overall binder volume fraction could increase further (see Equation (4)). Hence, the modeling also considered binder layers above the estimated thickness range of 7.0 to 10.5 nm (compare Figure 6B,C).

### 2.3 Transport Parameters of the System

The morphology of the anode PTE defines how water is transported to the CL and oxygen out of the system. Furthermore, electrical, ionic, and thermal conductivity depend on the system's structure and materials. Therefore, in the following section, the effective and relative transport parameters (one‐phase permeability, diffusivity, and conductivity) of the segmented CL, the CL including the modeled binder, and the PTL are calculated. Additionally, the interplay of these layers with respect to their transport parameters is considered. Overall, these transport parameters contribute to the efficiency and associated performance losses through ohmic losses (electrical and ionic conductivity) and possible mass transport overpotentials (permeability and diffusivity).

#### 2.3.1 Transport Parameters of the Segmented CL

**FIGURE 7:**
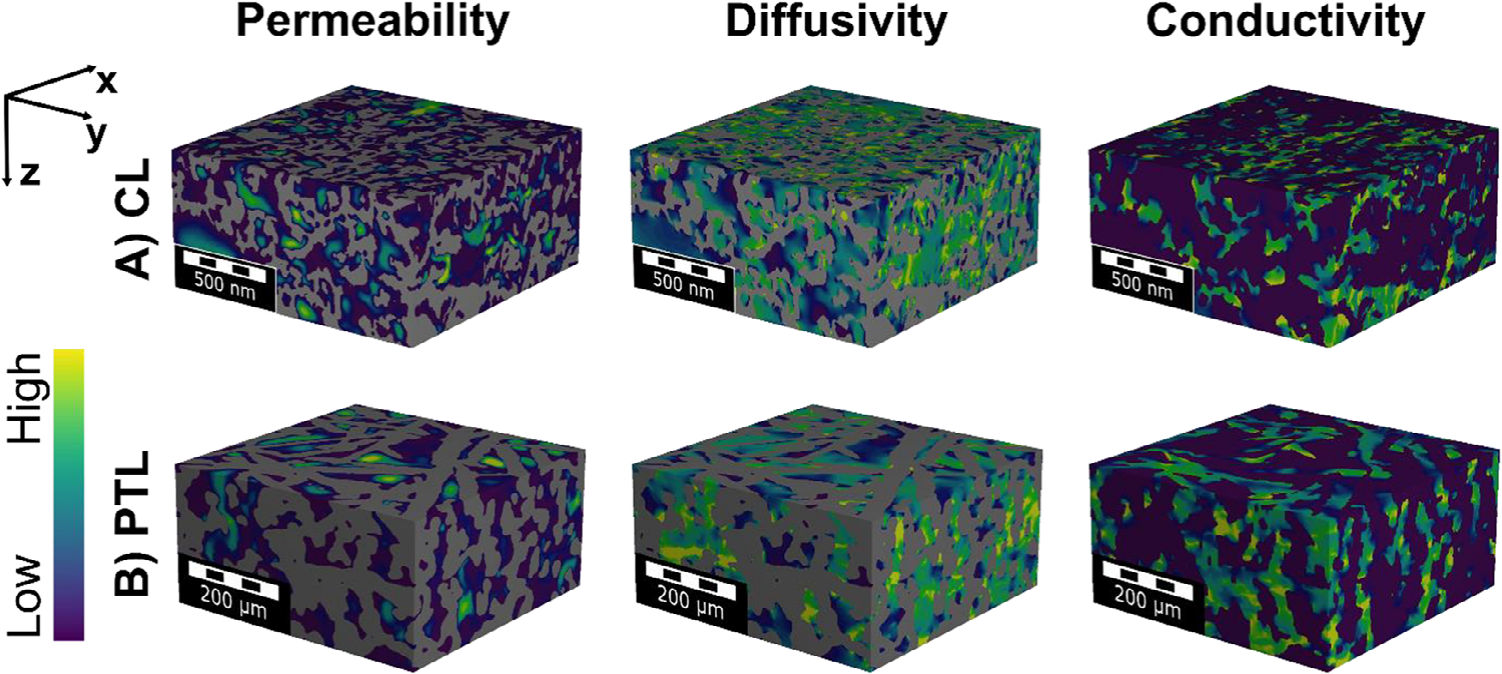
Visualization of the transport properties of the PTL and the CL. The one‐phase permeability, the bulk diffusion flux, and the electrical conductivity are depicted for small volume fractions of the full tomograms.

The Stokes flow, diffusion, and conductivity were simulated and visualized for a part of the full tomogram in Figure 7A. The values of the transport parameters were similar in in‐plane and through‐plane directions, indicating an isotropic structure (Table 1). Therefore, the mean value of the respective transport parameter was calculated. As the CL tomogram was split into three subvolumes, the obtained parameters for the single cubes are provided in the supplement (Supporting Information chapter “Transport parameters catalyst layer” and Tables S2–S32), while the data in Table 2 are averaged values of all three subvolumes. The permeability of 6.14 ∙ 10^−17^ m^2^ is lower than the one‐phase permeability values reported in literature [20, 40, 42] (between 10^−15^ m^2^ and 10^−16^ m^2^), which can be explained by the smaller pores of this CL that lead to a lower permeability. Lee [42] et al. also simulated the case of decreasing porosity and pore sizes, resulting in a permeability in the range of 10^−17^ m^2^. Based on the effective Knudsen and Laplace diffusivity, the diffusivity for the intermediate diffusion regime via the Bosanquet approximation was determined (Equation (2) and Table S16). Weber et al. [20] simulated the relative diffusivities of a core–shell anode CL with values in the range of 15% to 20%, whereas we found a relative diffusivity of around 30% (Tables S5 and S10). The 10% to 15% difference can be explained by the different porosity and PSD, which have a higher porosity and a smaller mean pore size for the herein investigated CL of the PTE.

**TABLE 1:** Effective transport parameters of the CL. The diffusivity values are the average effective diffusivity tensor of O_2_ in H_2_O from the tomogram's three subvolumes.

Direction	Permea‐bility [m^2^]	Knudsen diffusivity [cm^2^ s^−1^]	Laplace diffusivity [cm^2^ s^−1^]	Electrical conductivity [S m^−1^]	Thermal conductivity water/oxygen filled [W m^−1^ K^−1^]
IP (x)	5.85E‐17	1.54E‐05	1.80E‐05	3.41E+02	1.71/ 0.74
IP (y)	6.67E‐17	1.68E‐05	1.96E‐05	3.79E+02	1.74/0.81
TP (z)	5.90E‐17	1.44E‐05	1.76E‐05	3.40E+02	1.72/0.74
Mean	6.14E‐17	1.55E‐05	1.84E‐05	3.53E+02	1.72/0.76

**TABLE 2:** Effective transport parameters PTL

Direction	Permeability [m^2^]	Laplace diffusivity [cm^2^ s^−1^]	Electrical conductivity [S m^−1^]	Thermal conductivity water/oxygen filled [W m^−1^ K^−1^]
IP(x)	4.53E‐12	1.07E‐05	6.71E+05	7.04/6.22
IP(y)	4.82E‐12	1.10E‐05	6.79E+05	7.09/6.29
TP(z)	8.35E‐12	1.44E‐05	6.11E+05	6.69/5.68

The electrical conductivity of 3.25 ∙ 10^2^ S m^−1^ is similar to the CCM CL studied by Weber et al. [20], but lower than that reported by Lee et al. [42] due to the much lower porosity of the CCM CL that they investigated. The effective transport parameters of the electrical and thermal conductivity values should be treated cautiously. The electrical conductivity of IrO_2_ strongly depends on its film coating thickness and crystallinity [73, 74, 75]. Bernt et al. [75] showed a hundredfold increase in electrical conductivity by transforming amorphous IrO(OH)_x_ into crystalline IrO_2_ by applying a heat treatment. So, depending on the oxidation state of the used Ir catalyst, which is dependent on the manufacturing process and the operation conditions, the electrical conductivity can differ by several orders of magnitude. This affects the ohmic resistance of the CL and the catalyst utilization [39, 53]. Nearly no literature values are available for the thermal conductivity of IrO_x_. To the best of our knowledge, the thermal conductivity of an iridium‐based CL has never been experimentally determined, in contrast to Pt‐based CLs in PEMFCs [76, 77]. Furthermore, similar to the electrical conductivity, the thermal conductivity strongly depends on the crystallinity and the film structure [78, 79]. The value of 5.0 W m^−1^ K^−1^ was chosen under the assumption that no nanoplatelets are present and that IrO_x_ is forming a thin film [78]. Furthermore, we note that heat is generated in the CL during operation. This effect is not considered in our simulation results (the Poisson equation is no longer 0). Due to these uncertainties, particularly the thermal conductivity literature values of IrO_x_, a sensitivity analysis of the thermal conductivity of the CL, filled with water or oxygen, was performed using different thermal conductivity values of IrO_x_ (Table S30). The analysis shows that filling the pore space in the CL with oxygen instead of water has a similar effect to assuming a fivefold lower thermal conductivity of IrOx with water‐filled pores. Hence, bubble formation and mass transport are expected to govern the occurrence of hotspots [80]. Nonetheless, a lower thermal conductivity of the CL would decrease the ability to dissipate heat generated during electrochemical reactions, thereby increasing the thermal stress on the membrane. However, a higher membrane temperature caused by poor heat dissipation increases ionic conductivity due to increased proton mobility, thereby reducing ohmic resistance [81, 82].

**FIGURE 8:**
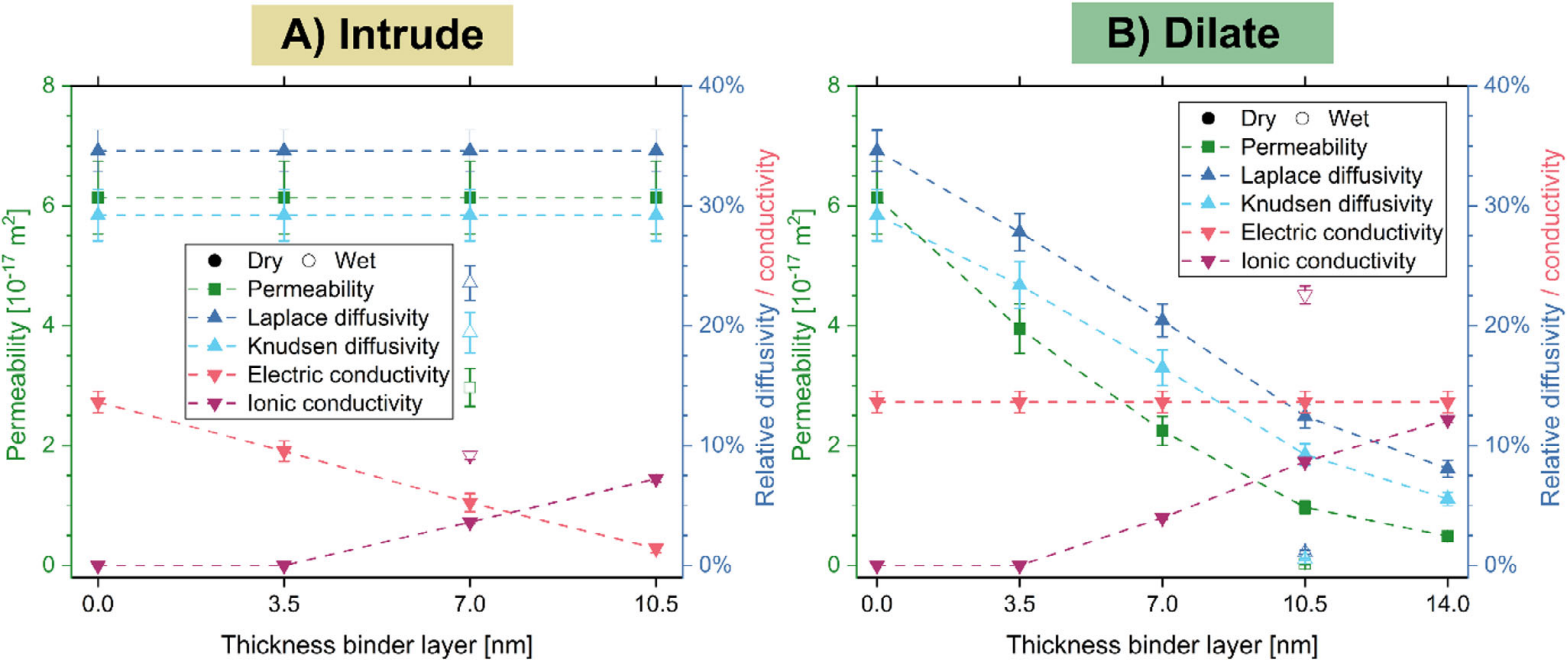
Transport parameters for binder intrusion (A) and dilation (B) with different binder thicknesses. Additionally, the transport parameters are shown with an assumed swelling factor of 1.8 under wet conditions. Due to the voxelized nature of the tomograms, the ionic conductivity of a binder layer consisting of only 1 voxel cannot be determined because the interface between two voxels can then consist of edges and no faces.

The different transport properties were additionally simulated under consideration of the modeled binder, taking both the intrusion and dilation approaches into account (Figure 8). When neglecting gas and liquid transport through the binder, the permeability and diffusivity values stay constant if the binder is intruded into the grain structure. As expected, the ionic conductivity then increases with increasing binder thickness, whereas the electric conductivity decreases due to the increasing binder fraction. For the dilation case, the porosity decreases with increasing binder film thickness, and therefore, the permeability and diffusivity decrease. The electric conductivity stays constant since the catalyst grains are not altered by this model. Overall, a larger binder fraction and a lower tortuosity of the binder phase decrease the proton transport resistance, which is equivalent to an increase in the ionic conductivity of the CL [52, 83]. For the intrusion and dilation cases, the tortuosity of the porous and binder phases falls within the range reported in the literature (Tables S31 and S32) [20, 40, 41]. However, it should be noted that literature data is only scarcely available and differs in catalyst type, binder fraction, and method of manufacturing.

In general, the ionic conductivity of a thin Nafion film depends on thickness, humidity, and temperature, and increases with increasing film thickness [84]. Therefore, the ionic conductivity is expected to rise more steeply than depicted in Figure 8 since the simulation was performed with a thickness‐independent ionic conductivity of Nafion. Keeping in mind the operational conditions of the PEMWE, the swelling of the binder must be considered. As a result, the additional binder volume in both scenarios leads to a decrease in permeability and diffusivity and an increase in ionic conductivity (Figure 8). Possible changes to the catalyst particle structure caused by the swelling of the ionomer were not considered.

Furthermore, different binder models can be considered as alternatives to modeling a homogeneous binder layer, altering the binder distribution and thereby influencing the CL transport parameters [30]. Here, the “Add Binder” function of GeoDict was chosen, as a common approach in several binder modeling papers (Figure S21A) [30, 40]. A comparison with the dilation scenario showed similar permeability values, but lower diffusivity values (Figure S21B). The reason is the preferred filling of smaller to larger pores in the binder generation process, which leads to a clogging of small pathways and, therefore, a higher tortuosity (Figure S21A). The electrical conductivity remained unaffected, but the ionic conductivity also increased with increasing binder content (Figure S21B). However, the ionic conductivity exhibited a slight increase in the dilation scenario due to the homogeneous and uninterrupted nature of the binder network, as a consequence of the constant binder thickness. Consequently, the diffusivity values and the ionic conductivity values of the dilation scenario can be interpreted as upper limits of the respective transport properties under the assumption that the binder has been removed during the tomography. Nevertheless, both auxiliary models showed the same trends and transport parameters on the same length scale. Despite the core–shell structure suggested by the presented STEM‐EELS results, future research should aim for even better‐resolved tomograms of the catalyst‐binder structure, as achieved in PEMFC via high‐resolution cryogenic transmission electron tomography [51], to improve possible binder models and thereby enhance the accuracy of the simulated transport parameters.

Additionally, the reconstructed CL with modeled binder can be used for digital material design‐aided predictions on the performance of PTEs. If the IrO_2_ catalyst skeletons of the intrusion and dilation scenario that meet the 1.5 volume ratio between binder and catalyst particles remain identical with varying binder content, an arbitrary amount of binder can be modeled around the solid IrO_2_ phase. Therefore, as the catalyst volume fraction is known, the exact binder volume can be added around the catalyst skeleton to match the desired binder content. The porosity, solid volume fraction, and binder phase were calculated with Equation (5) for different binder weight percentages. These modeling assumptions about binder and pore volume are in accordance with PEMWE and PEMFC literature. Generally, the CL thickness remains unaffected by varying binder content [85, 86, 87]. In contrast, the binder thickness increases and the porosity decreases as the binder content increases [51, 52, 85, 88].

The PTE analyzed here was manufactured according to the recipe presented in the binder optimization study by Bühler et al. [10] (same ink, PTL, and spray‐coating process). The PTEs in their study contained 5, 9, 12, and 21 wt.% binder [10]. The above‐described digitally generated CLs can explain the worse electrochemical performance of PTEs with higher binder content due to mass transport limitations (Figure S22). FIB cross sections presented in their study [10] showed partly completely binder‐filled pores at 21 wt.%. Under operational conditions, the swelling of the binder decreases the porosity of the system nearly completely (12 wt.%) or fully (21 wt.%) in the dilation model. Even for the intrusion model, the porosity drops drastically to around 12% for 21 wt.% binder content. These mass transport limitations could lead to a higher overpotential at high current densities. On the other hand, the best performing PTE, with 5 wt.% binder, had a porosity between 40% and 63%. In wet conditions, the binder volume fraction increases to between 13% and 21%, resulting in much lower mass transport resistance due to better water transport and faster removal of the produced O_2_.

**FIGURE 9:**
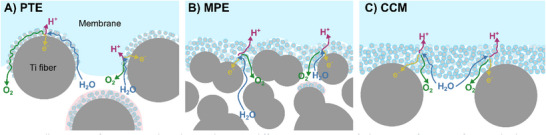
Illustration of reactant and product pathways in different MEA setups. (A) The PTE configuration features both ionically well‐connected areas close to the deformed and swollen membrane and ionically disconnected catalyst areas (colored in red) at increasing depth below the PTE surface. (B) Using an MPE makes the CL structure more homogeneous (similar to a CCM configuration), leading to an overall increase in catalyst utilization. (C) In CCM configurations, a homogeneous CL is present. Depending on the ratio of electric to ionic conductivity, there exist CL regions that contribute less to the OER due to limited protonic conductivity (regions further away from the membrane) or limited in‐plane electrical conductivity of the CL (regions between the PTL fibers) [5, 34, 53, 91].

The low ideal binder content identified by Bühler et al. [10] is in contradiction to the optimum binder content in literature for CCM setups. E.g., Bernt et al. [52] determined the optimum binder content of a CCM‐based anode CL as 11.6 wt.%. Their IrO_2_/TiO_2_‐based, and Mayer‐rod‐coated CL had a porosity of 35% and a wet‐binder volume fraction of around 35%. These values differ noticeably from the ideal binder content for the PTE identified by Bühler et al. In the presented PTE configuration, comparably large titanium fibers are coated with a thin, approximately 1 µm thick CL, which results in deeper, poorly connected catalyst areas and more superficial catalyst areas that are in direct contact with the membrane [20, 47]. Further, the uncoated membrane penetrates into the PTE structure due to the high applied clamping pressure and the swelling of the ionomer, which reduces the contact resistance for the more superficial catalyst areas. Consequently, we hypothesize that highly porous CLs that enable quick water supply and oxygen removal are more critical in this PTE configuration than good protonic pathways due to the proximity of the membrane, since the deeper catalyst areas do not contribute significantly to the reaction anyway (Figure 9A). In contradiction, the CLs of CCM‐based MEAs are much thicker (around 8–12 µm for Bernt et al. [52]), and the proton conduction pathways for accessible catalyst areas are much longer. Therefore, a higher binder content is supposedly preferable to ensure a good ionic network (Figure 9C). When using a titanium substrate with an MPL, the PTE or MPE configuration is more equivalent to a CCM configuration with regard to catalyst thickness, ionic pathways, and catalyst utilization [20] (Figure 9B). Therefore, we suggest the usage of higher binder contents in future MPE‐based setups with a lower surface roughness compared with the PTE system investigated here. Additionally, bigger catalyst particles, like core–shell particles [11, 20, 89, 90], could be beneficial to ensure sufficient pore space for good reactant and product transport. This hypothesis is strengthened by the results of Weber et al. [20], who showed a good performance of a spray‐coated MPE with a porosity of around 36% and a wet binder volume fraction of around 33%.

#### 2.3.2 Transport Parameters PTL

Additionally, the transport parameters of the bulk PTL were determined and are displayed in Figure 7B. The PTL is anisotropic, with a higher permeability in the through‐plane direction, which is in line with other results of fibrous PTLs in literature [5, 92]. In contrast, powder‐based PTLs show a more isotropic behavior in transport properties [20, 92]. The fluid transport in the PTL is dominated by the Stokes flow. Diffusion processes play a minor part, with the highest flux through larger pores and diffusion maxima in pore throats. The main diagonal elements of the transport tensor can be found in Table 2, and the full tensors in the supporting information (Supporting Information chapter “Transport parameters PTL” and Tables S33–S44). The permeability tensor, resulting from the geometrical structure, was determined for a one‐phase flow. However, we note that the flow during electrochemical operation depends on oxygen saturation and the accompanying bubble formation, which results from current density and PTL morphology [93]. Yet, this two‐phase flow could only be analyzed experimentally via *operando* X‐ray tomographic microscopy at synchrotron facilities [93, 94] or simulated via methods with high computational effort, e.g., the lattice Boltzmann method [95, 96]. The electrical and thermal conductivities in the PTL are nearly isotropic. While the electrical conductivity is constant, the thermal conductivity depends on the oxygen saturation, depending on the operation point. It lies between the two extremes of the PTL being completely filled with water or oxygen. Tortuosity and conductivity values are in agreement with literature values [5] (Tables S38 and S42).

#### 2.3.3 Transport Parameters PTE

The previous two sections determined the individual transport properties of the bulk CL and the bulk PTL. The PTE has a lower porosity compared with a plain PTL because of the infiltration with CL. Consequently, the diffusivity and permeability are changing. As an approximation, the overall transport properties of the PTE were simulated from the tomographic Micro‐CT reconstruction of the whole PTE (Figure S3), choosing different cuboid volumes, one where the solid volume fraction exceeds the mean solid volume fraction of 49.16% (volume PTE 1) and one where the solid volume fraction exceeds the 5% threshold of the mean solid volume fraction (volume PTE 2). Two factors must be considered when comparing the PTE with the bulk PTL [47]. First, an uncoated PTL has a lower solid volume content at the top and bottom sides, which is due to a lower fiber density at these surfaces. Second, the PTE has an overall higher solid volume content compared with the bulk PTL, which is due to the added CL at the electrode side of the PTE (Figure S24B). Volume PTE 1 and the bulk PTL have similar transport properties because the solid volume fractions are similar. The catalyst‐coated titanium fibers with a penetration depth of around 100 µm are only a minor fraction of the overall titanium fiber network with a thickness of around 1 mm [47]. Meanwhile, volume PTE 2 has worse TP conductivity and higher IP permeability due to the highly porous volume at the outer edges (Table S45). Notably, the transport properties of an operating electrolysis cell also depend on the interfaces of the PTE with its surrounding layers, e.g., the penetration depth of the membrane into the PTE [5].

**FIGURE 10:**
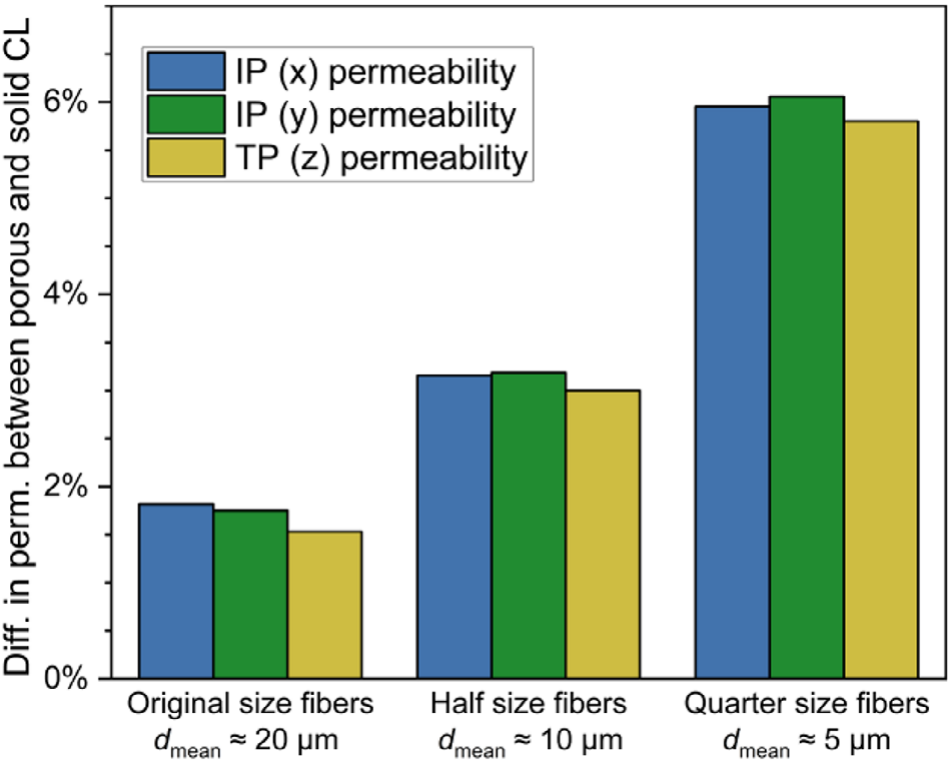
Difference in permeability of a PTE between modeling a porous and a solid CL with a thickness of around 1 µm. The increasing difference can be ascribed to the rising solid volume fraction of the CL.

A single tomography that covers the full PTE comes with restrictions such as an insufficient resolution to take the porosity of the CL into account, and the difficulty in distinguishing between the different material systems (titanium PTL and IrO_x_‐based CL). Therefore, multiscale tomography is necessary for a more accurate and locally resolved analysis of the transport properties of the PTE. This approach allows the combination of both the CL and the PTL characteristics to enable the determination of transport parameters of the PTE by modeling the CL around the titanium fibers. Due to the different length scales of the CL and the PTL, directly modeling the CL onto the fibers would require a huge computational effort to calculate the transport parameters. E.g., a PTE volume of the size (100 µm)^3^ and a voxel size of 3.5 nm (to resolve the CL) would have around 2.3 ∙ 10^13^ voxels, which would correspond to at least four orders of magnitude larger simulation datasets and computational times than the previously presented simulation. Therefore, the porous CL was modeled as a solid layer with effective transport properties determined in Section 2.3.1. “Transport parameters CL” (Figure S23). The catalyst distribution in the PTE is known [47], which allows for modeling the PTE by a homogeneous addition (1 to 6 voxels) of this catalyst amount in the reconstructed PTL around uncoated titanium fibers, which serves as an approximation of the PTE (Figure S24A,B). The porous volume decreases with the rising number of added voxels (Figure S24A). The addition of 6 voxels matched the maximum of the CL distribution, with a solid volume fraction of around 56% (Figure S24B). In the next step, the permeability values were computed. Notably, even for the 6‐voxel‐CL, the difference in permeability between a porous (porosity and transport properties as an effective medium approach) and a solid (without any porosity) CL was less than 2% (Figure 10; Table S47). Therefore, to save computational time, the CL was simplified as a solid layer for the calculations with thinner CLs (5 to 1 voxel CL thickness). As expected, permeability and diffusivity values decreased with a rising amount of CL, due to a loss of porous space (Figure S24C and Table S48). Notably, the effect of the CL on electrical conductivity is negligible. Even if the whole porous space was filled with the CL and possible interface resistances are neglected [97, 98], the electrical conductivity would stay nearly constant due to the almost 1000 times higher bulk conductivity of titanium than of iridium oxide (Table S46).

These simulations show that the porous pathways in the CL do not significantly contribute to the overall permeability. The overall permeability is dominated by the porous space between the coated fibers. However, taking into account these porous pathways in the CL will be indispensable for future PTE configurations with more MPL‐like substrates. MPL‐like structures consist of fibers or particles of smaller diameters than the titanium fibers of the bulk PTL, and simulations of the CL modeled onto thinner fibers show that the difference in permeability between modeling the CL as an effective medium vs. a solid structure increases with decreasing fiber thickness (Figure 10; Figure S25). The finer structure of the thinner fibers leads to higher solid volume fractions of the catalyst layer within the PTE and partly closed pores. As a result, the permeability of the CL becomes more important for the transport pathways of the coated substrate. However, using an effective medium approach for the CL has its limitations when analyzing MPEs instead of PTEs. When transitioning from MPLs reported in the literature, e.g., the MPL used in the publication of Weber et al. with a mean Ti particle diameter of 11.5 µm and a mean pore diameter of 11.3 µm [20], to nanoscale MPLs, such as those employed in fuel cells [30], the interface between the MPL and the CL requires more detailed investigation, particularly with respect to the structure and connectivity of the porous network. Hence, in such MPE approaches, especially when they show high catalyst concentrations in the MPL [20], a differentiation between different layers in graded PTEs is important, and it is highly recommended to model the CL as a porous layer with its associated transport properties and to simultaneously account for the different length scales involved. Furthermore, possible limitations in this approach may occur if bubble dynamics or two‐phase flows inside the PTE are simulated. Nonetheless, in this case, with a PTE that shows a stark change in pore and grain sizes between PTL and CL, these limitations do not apply, but the benefits from reducing computational efforts are in favor of the employed effective medium approach.

Overall, this approach demonstrates that data from multiscale tomography can be used as digital twins in future PTE simulations to minimize possible errors in transport and electrochemical simulations of the cell.

## 3 Conclusions

We conducted the first multiscale tomography of an anodic PTE in a PEMWE. The tomography and imaging covered several orders of scale, from more than ten µm thick fibers, over the around 100 nm big pores and grains of the CL, to the individual catalyst particles and the surrounding binder on the single‐digit nm scale, enabling a deeper understanding of PTEs in PEMWE. Micro‐CT data showed that the titanium fibers of the PTL are mainly horizontally aligned and have a diameter of around 20 µm [47]. FIB‐SEMt of the CL revealed a log‐normal pore size distribution with a mean grain diameter of around 80 nm. Comparing the data with a BET analysis of the pure iridium oxide catalyst, we concluded that the mean grain consists of up to 180 individual IrO_x_ catalyst particles.

As a further step in this multiscale approach, a catalyst‐coated Ti fiber was analyzed via FIB‐SEMt, which showed a highly heterogeneous CL. The mean CL thickness of 1.0 µm in the topmost layer of the PTE was in rough agreement with our previous estimation of 1.3 µm, but the detailed tomography revealed a larger thickness on the top surface and a reduced thickness on the side edge of the fiber [47]. FIB‐SEMt allowed this valuable insight into the CL thickness distribution. Still, it also showed that the heterogeneity of the CL requires the development of physical models to analyze the CL‐fiber interface locally and globally, given the small field of view and required effort of FIB‐SEMt. Via STEM‐EDXS and EELS analyses, the overall presence of the binder in the CL was qualitatively detected, but beam damage prevented obtaining quantitative information on its distribution. Cryo‐TEM tomography, as performed in PEMFC research [51], is a possible way to obtain this information in future studies. Nevertheless, the potential of low‐loss STEM‐EELS was shown to distinguish the catalyst and binder in anodic CLs. As a substitute, the binder in the CL was modeled. The mean binder thickness was estimated to be between 7 and 10.5 nm, based on TGA analysis, and in line with results from PEMFC [51, 58, 60].

Subsequently, the tomography data were used to calculate transport parameters for the CL under dry and wet conditions, as well as the transport parameters of the PTL. Notably, the PTL showed a higher through‐plane than in‐plane permeability and diffusion, while the conductivity was isotropic. On the other hand, the CL was nearly isotropic for all transport parameters investigated. The different binder models influenced the simulation results for the transport parameters of the CL due to differences in pore or grain sizes or binder distributions. Consequently, the transport parameters simulated herein do not yield an exact value, but rather a possible range. To address this uncertainty, future studies should combine high‐resolution TEM imaging with physical models to better characterize binder behavior. Simulations of the swelling of the binder under wet conditions indicated lower porosity and, consequently, lower permeability.

The transport properties of the complete PTE were determined, and it was shown that the overall transport properties of the presented setup are mainly dominated by the titanium fiber PTL. A comparison of modeling the CL as an effective medium vs. a non‐porous solid revealed that the influence of the CL on the transport properties of PTEs increases with decreasing fiber thickness. Therefore, simulation studies on MPEs require accurate determination of the transport properties of PTL, MPL, and CL to obtain accurate overall data on the transport parameters of the MPE.

In summary, these results help to better understand the structural and transport parameters of a PTE. The reconstruction of the CL, including modeling the surrounding binder, modeling the spray coating process [30], and modeling the CL with its transport properties around the PTL via an effective medium approach, are important steps in laying the foundation for digital material design of PTEs. For example, we were able to explain the results of Bühler et al. [10] regarding mass transport problems and optimum binder content by digital material design, and put them into context with literature results from CCM research. Our results predict improved transport properties for the usage of titanium substrates with an MPL and suggest that higher binder contents of more than 5 wt.%, and the usage of catalyst particles bigger than ∼100 nm CL grains, such as core–shell particles [11, 89] or supported catalyst structures [90], can improve water and oxygen transport in such graded systems. In the future, a coupled electrochemical model can directly connect the structural and transport properties to the electrochemical performance of the electrolyzer, yielding more quantitative results. First steps in this direction are presented in a study by our group [99], where we utilized the here presented tomography of the CL and its transport properties as input parameters for an electrochemical model to investigate the influence of morphology and transport parameters on the performance of a CCM configuration.

The combination of µCT, FIB‐SEMt, and supportive TEM imaging is a powerful toolbox to investigate challenging samples that span multiple scales. This methodology not only has the potential to assist in the rational design of PEM water electrolyzers and modeling their performances [39], but it can also be applied to other systems, such as AEMWE [100, 101], or the analysis of GDEs [46, 102], and the information gained can be applied to CLs of CCMs as well. A comparison with the only scarcely available literature reports of tomography of differently manufactured and composed electrodes emphasizes the need for a large database of CL structures. Furthermore, a deeper understanding and fundamental research of the intrinsic and extrinsic properties of the materials and their composition, ranging from the thermal conductivity of CLs to more accurate binder distributions in CLs, are essential to improve the accuracy of simulations. In total, a broad overview of systems and materials is needed to approach a digital material design library that supports the targeted optimization of porous media in electrochemical energy systems.

## 4 Experimental Section

### 4.1 Sample Fabrication

This publication was a follow‐up to our previous work on the catalyst distribution of PTEs [47]. PTE samples of the same batch or even the same sample for investigations via micro‐CT and FIB‐SEMt (Figure S3) were used for the structural analysis and the subsequent simulation of transport parameters. Details about the MEA fabrication according to the recipe of Bühler et al. [10] and the electrochemical characterization are provided in our previous publication [47]. Here, the manufacturing is briefly summarized:

Ultrasonic spray coating (Exacta Coat, Sonotek) was used to manufacture the PTEs. Sintered titanium fibers (2GDL40‐1.0 Bekaert) with a porosity of 56%, a mean fiber diameter of 20 µm, and a size of 5 mm x 5 mm were used as a substrate. The catalyst ink consisted of IrO_2_ (Premion, Alfa Aesar) and Nafion (D520, Chemours). The final CL had a loading of 1.5 mg cm^−2^ iridium oxide and a binder content of around 10 wt.%. Additional CL samples with the same catalyst ink and spray‐coating procedure were produced on glass (microscope slides, DURAN WHEATON KIMBLE) for TEM analysis.

### 4.2 BET Analysis

Nitrogen physisorption was used to determine the specific surface area of the IrO_x_ catalyst with a surface area and porosity analyzer, Tristar II Plus (Micromeritics). Samples were degassed for 16 h at 150 °C at < 0.05 mbar before being weighed and measured. Afterward, nitrogen adsorption and desorption isotherms were recorded at 77 K. The N_2_ adsorption isotherm was evaluated in the relative pressure range from 0.05 to 0.35 according to the Brunauer–Emmett–Teller (BET) theory [103] to determine the specific surface area (see Supporting Information chapter “BET analysis”). In total, two individual BET measurements for the same IrO_x_ catalyst batch were performed.

### 4.3 STEM Imaging and Spectroscopy Analysis

Different sample preparations were necessary for the STEM imaging and spectroscopy analysis. For the CL, the catalyst ink was spray‐coated onto glass slides with parameters equal to those for the PTE production. Then, the TEM sample was prepared by dry‐dispersion, according to Girod et al. [51] For this procedure, parts of the CL were scratched off the glass substrate with a scalpel blade. Aggregates were carefully crushed between two glass slides. Then, the CL particles were transferred onto TEM grids by dipping a TEM grid (Lacey carbon film on finder net, Plano) several times into the powder.

A small amount of the catalyst powder was dissolved in ethanol to image pristine catalyst particles. The suspension was homogenized for several minutes in an ultrasonic bath. Immediately afterward, a single droplet was drop‐casted onto a lacey carbon‐coated copper TEM‐grid with an Eppendorf pipette.

The CL as well as single particles were investigated using HAADF‐STEM and STEM‐EDXS (Talos F200i, Thermo Fisher Scientific) at an acceleration voltage of 80 and 200 kV, and double‐corrected STEM‐EELS (Titan^3^ Themis 60–300, Thermo Fisher Scientific, with a Gatan Quantum ERS spectrometer) at 300 kV.

The STEM‐EELS experiments used a 15 pA probe current, a pixel dwell time of 10 ms, and a spectrometer dispersion of 0.5 eV/channel. DualEELS was enabled, with loss‐loss set to [−100, 924] eV and 50 µs exposure and high‐loss set to [200, 1224] eV and 9.95 ms exposure time. In this way, we acquire both the intense zero‐loss and plasmon peak at the initial stage of electron beam irradiation and a longer time for the core‐loss range for possible identification of the component. The pixel sampling was adjusted between 1.1 and 4.8 nm/pixel. Thus, the total (instantaneous) fluence for the low‐loss signal range was between ∼20 and ∼380 e^−^/A^2^, and the high‐loss signal range between ∼4000 and ∼77400 e^−^/A^2^.

### 4.4 Scanning Electron Microscopy and FIB‐SEM Tomography

A Zeiss Crossbeam 540 FIB‐SEM microscope with a Gemini II column was used for SEM imaging of sample surfaces and FIB‐SEMt. Samples were attached to aluminum SEM specimen stubs (G301 and G399, Plano GmbH) with conductive carbon pads (G3347 and G3348, Plano GmbH). The samples were sputter‐coated either with Gold (108 Manual Sputter Coater, Cressington) or with Carbon (Balzers Union, MED 010) before imaging and contacted with conductive silver paint (G3692, Plano GmbH) at the edge to obtain better conductivity. The surface of the PTE was imaged via a secondary electron (SE) detector with an accelerating voltage of 3 kV and a current of 750 pA.

FIB‐SEMts were performed with the Zeiss software package Atlas 3D Nanotomography, similar to our previous publication [30]. Two types of FIB‐SEMts are presented in this paper, one that only reconstructs a partial volume of the CL (tomography CL) and one that includes the upper half of a titanium fiber and the spray‐coated CL (tomography spray coated titanium fiber). The presented tomographies were at the resolution limit of FIB‐SEMt, mainly due to the poor electric conductivity of iridium oxide and the comparably long conduction pathway of the fibrous titanium PTL. Sputter coating and drift correction during the acquisition were performed to minimize the drifting effects due to charging.

The FIB‐SEMt of the CL was performed on a horizontal plane of a top edge fiber (Figure S3). FIB‐SEMt requires the deposition of a highly conductive, protective layer on top of the sample to improve image quality. Here, Pt was used for this purpose. However, due to the similar Z numbers between Pt and Ir, a layer of carbon was deposited as a contrast layer before depositing the protective Pt layer (Figures S26 and S27). Trenches were cut laterally from and in front of the selected area to expose the cross‐section of the CL and to minimize redeposition effects. Afterward, auto‐tune and tracking markers were cut to ensure stability (i.e., constant cutting slice thickness and sharp imaging procedure). These markers were highlighted with carbon before a protective carbon layer was deposited on top. The cross‐section was polished with a FIB current of 100 pA to smooth the surface before serial sectioning.

Serial sectioning was performed with a nominal slice cutting thickness of 7 nm using a FIB current of 50 pA and a voltage of 30 kV. The average slice thickness of 7.335 nm was determined by the tracking markers. Tilt‐corrected images with a pixel size of 3.5 nm were recorded with two SE detectors (Everhart‐Thornley and In‐Lens SE detector) applying an accelerating voltage of 3 kV and a current of 750 pA. The small pixel size was necessary to resolve the CL features (Figure S5). The information from the Everhart‐Thornley detector was used to reconstruct the CL volume, and the In‐Lens SE detector was used to scan the markers during the imaging acquisition.

The data acquisition of the spray‐coated titanium fiber was conducted similarly to the tomography of the CL. Here, an area was chosen that contained a fiber that was parallel to the surface of the PTE over more than 20 µm (Figure S12). The same protective and contrast‐enhancing layers of platinum and carbon as for the CL tomogram were deposited on the sample. However, no lateral trenches were milled for the preparation, as the whole fiber diameter was investigated (Figure S27). A targeted slice thickness of 10 nm resulted in a final average cutting thickness of 10.4 nm. The pixel size was chosen as 10 nm.

The exact FIB‐SEM parameters for both tomographies are summarized in Table S49.

### 4.5 Segmentation, Image, and Data Processing of FIB‐SEM Tomograms

For both tomographies, the acquired image series were registered using stationary background features. Then, the images were transformed using in‐house developed Matlab (Mathworks) scripts to account for the acquisition angle *α* (angle between FIB and SEM beam, 54° for this FIB‐SEM) and the slice thickness *d*. The process consists of a downward shift of *d ⋅* cos(*α*) on each image. A detailed explanation can be found in our previous publications [104, 105].

Several subvolumes of the CL tomogram were used for the subsequent steps. This procedure was required as the evaluation of structural parameters requires cuboid volumes. A single cuboid volume was not considered sufficient for a representative analysis due to the low thickness, high surface roughness, and the variance in the thickness of the CL. Thus, three cubes were selected from the full tomogram and further processed (Figure S4). The subvolumes were manually selected by choosing regions with a CL thickness of at least 900 nm to ensure a large volume filled with CL. The dimensions of the subvolumes are provided in Table 3.

**TABLE 3:** Overview of the used tomography and imaging methods. Results of the structural analysis of the PTL and the catalyst distribution are taken from our previous publication [47].

Sample area	Method	Pixel/Voxel length	Segmented area or reconstructed volume
CL	FIB‐SEMt	3.5 nm x 3.5 nm x 7.3 nm	2.35 µm x 1.30 µm x 2.60 µm; 4.80 µm x 0.91 µm x 2.43 µm; 4.59 µm x 0.95 µm x 3.31 µm
Catalyst‐coated fiber	FIB‐SEMt	10.0 nm x 10.0 nm x 10.4 nm	20.8 µm along the fiber
Catalyst distribution	BSD [47]	97.7 nm	4.11 mm x 0.20 mm; 3.16 mm x 0.20 mm; 4.14 mm x 0.20 mm;
PTE/PTL	Micro‐CT [47]	2.5 µm	3.00 mm x 4.25 mm x 1.16 mm
Binder	STEM‐EDXS	596 pm (80 kV), 284 pm (200 kV)	Several images
Binder	SEELS	1.1 nm, 3.8 nm, 4.8 nm (300 kV)	Several images

The segmentation of the tomograms was performed in GeoDict (Math2Market GmbH). Initially, several filters were applied to improve the image quality of each dataset (Figure S28): Flickering gradients and brightness deviations within the image stack were removed by applying a gradient brightness correction. A non‐local mean filter was executed to denoise the 3D dataset. A sharpening filter was run to improve the visibility of the boundaries between pore and grain.

The segmentation was done using an Unet 3D algorithm in GeoDict to distinguish between grain and pore (Figure S29) [106]. This 3D AI‐based segmentation was chosen to segment different gray‐value features and avoid typical shine‐through artifacts. A direct comparison between this AI segmentation and a standard Otsu segmentation shows the improved segmentation (Figure S30). Nevertheless, segmentation artifacts cannot be totally avoided. They were minimized by training the Unet 3D model iteratively using manual labels for grains and pores in all directions and successively checking via manual inspection (Figures S29 and S30). The trained model was used to segment the individual subvolumes. Then, the image stack was resized to account for the larger cutting distance (7.335 nm) compared with the pixel pitch of the single images (3.5 nm). The voxel size was set to the pixel size of 3.5 nm using bicubic interpolation with an in‐house developed Matlab script [105]. The smaller voxel size of 3.5 nm was chosen for a possible finer gradation in subsequent steps for binder modeling. The segmented and resized volumes were smoothed by applying morphological image processing algorithms (opening and closing with a 1 voxel mask). Finally, the segmentation was manually inspected, and the Unet 3D model was improved if necessary.

The segmentation of the titanium fiber dataset was done by processing the whole tomogram with an Unet 2D algorithm implemented in GeoDict after image registration, as described above [106]. The model was trained with images of different slices. This segmentation differentiates between background, titanium fiber, and CL area (Figure S11). Additionally, a cleansing step was performed to remove unconnected fiber and catalyst pixels, applying a cleanse‐operation implemented in GeoDict. Furthermore, the dataset was carefully inspected and manually corrected for false segmentation results if necessary. Afterward, the segmented CL area was used as a mask and applied to the raw grayscale image dataset to obtain a raw dataset consisting only of the CL. A local Otsu segmentation was used via GeoDict to differentiate between pore and grain of the CL (Figure S11). Then, post‐processing was applied to improve the segmentation. On the one hand, a connectivity analysis of the CL was done to identify unconnected catalyst grain particles and to remove them. On the other hand, the interface between the CL and the titanium fiber was smoothed by applying a morphological opening and closing operation.

### 4.6 Structural Analysis of Tomograms

The structural analysis of the tomograms was performed using GeoDict. The packages ProcessGeo, MatDict, PoroDict, and GrainGeo were used to determine the porosity and the grain and pore size distributions. The average structural properties of the CL were calculated by taking the individual structural properties of the analyzed subvolumes weighted by volume. The standard deviation for a discrete probability distribution was taken as an error for all structural parameters. A bin size of 3 voxels was chosen to compare the GSD and PSD of the individual tomograms. A representative volume analysis in the form of a quadrant analysis, suggested by McLaughlin et al. [47], was performed. Therefore, the individual subvolumes were bisected along the two in‐plane directions (the through‐plane direction was omitted due to the low thickness). The standard deviation and coefficient of variation of the porosity served as a measure of the representative character of the subvolumes.

The surface area *A*_FIB‐SEMt_ of the CL was estimated with GeoDict using a staggered grid approach, which compensates for the discretized voxels of the tomogram [107]. The specific surface *S*_FIB‐SEMt_ is calculated by dividing surface area by volume. *S*_FIB‐SEMt_ must be converted from m^2^ m^−3^ to m^2^ g^−1^ to compare it against the specific surface area *S*_BET_ of the N_2_ adsorption measurement. Knowing the in‐plane area *A*_IP, FIB‐SEMt_ of the tomogram and the fraction in thickness *t*_TP, FIB‐SEMt_/*t*_total_, the mass referenced specific surface area *S’*_FIB‐SEMt_ can be expressed and calculated as:

(1)

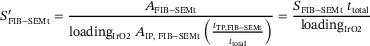




The CL loading of 1.5 mg cm^−2^ iridium oxide with an estimated error of ±0.1 mg cm^−2^, the in‐plane area *A*_IP, FIB‐SEMt,_ and thickness *t*_TP, FIB‐SEMt_ of the analyzed volume are known. The total thickness *t*_total_ of the CL was estimated in our previous paper [47] between 5.01 and 6.44 µm and, therefore, assumed to be the mean value of 5.73 ± 0.72 µm. The calculation error was approximated with the total differential of Equation (1).

The CL thickness on the fiber was determined using an in‐house‐written MATLAB script. The challenge in obtaining a meaningful layer thickness was given by the non‐planar substrate, consisting of fibers with a non‐circular circumference. The principle of the analysis is illustrated in Figure S14. Individual 2D slices were used for the thickness evaluation instead of the 3D structure. This approach was valid since the tomogram was performed on a fiber that was oriented parallel to the through‐plane direction of the stack. In the first step, the interface between CL and the titanium fiber of each slice was determined by the Canny edge detection algorithm [108] that was applied on the segmented 2‐D images using MATLAB. If the interface line consisted of more than one pixel, only the outer pixel was considered, so as not to falsify the later thickness analysis (Figure S31). Afterward, the thickness of the CL was determined at each point of the interface line by calculating the length of the normal between the fiber‐catalyst interface line and the outer edge of the CL (Figure S14). The normal of each point of the interface line was determined by applying an iterative algorithm based on the distance transformation of the inverse of the fiber.

Several geometric error sources were evaluated when determining the CL thickness. If the fiber has a tilt or the cutting direction was not entirely parallel to the fiber, an overestimation of the CL thickness occurs. Another possible issue was the definition of the outer CL edge, namely, whether a pore at the outer edge was considered part of the CL or not. This can lead to an error of up to the mean pore size times the porosity. However, these factors (Figures S15, S32, and S33, and Supporting Information section “Possible geometric CL thickness error”) showed a negligible effect and were, therefore, not considered in the further thickness analysis.

Since the CL was deposited on the fibrous substrate using a spray‐deposition method, a difference in layer thickness between the horizontal top surface of the fiber and the side edges can occur. Hence, the calculated thickness was further divided into top‐edge and side‐edge thicknesses. They were obtained by considering the slope of the normal between the catalyst‐fiber interface and the outer catalyst edge. If the slope angle was between 45° and 135° (when the absolute value of the arctangent was greater than π/4), it was considered part of the top‐edge thickness. Otherwise, it was counted as part of the side‐edge thickness (Figure 4). Additionally, a through‐plane dependent CL thickness was determined that was further distinguished between the left and right sides relative to the fiber center.

The tomogram and structural data of the bulk PTL and the PTE without differentiation between the titanium fiber and CL (obtained via micro‐CT) were taken from our previous publication [47]. Data on the catalyst distribution via embedded cross‐sectional imaging were also taken from this publication. Table 3 summarizes the investigated volumes and regions on the PTE.

### 4.7 Calculation of Transport Parameters

Transport parameters were calculated using GeoDict, similar to previous publications of our group [30, 109]. A comprehensive overview of the physical and mathematical equations can be found in the supplement (“Theoretical background of the simulation of transport parameters”). The tool was used to obtain the permeability, the Reynolds number (FlowDict), the tortuosity, the diffusivity, the Knudsen number (DiffuDict), and the electrical and thermal conductivity (ConductoDict). The GeoDict LIR‐solver was used to solve the differential equations (simulation stopping criterion 0.001) if not stated otherwise. Symmetric boundary conditions were used in the computation direction and periodic boundary conditions in the corresponding tangential directions. The transport tensors were calculated, dependent and independent of the material properties, if reasonable.

Flow and permeability were determined via the Stokes equation and Darcy's law [110]. Bulk and Knudsen diffusion were simulated with the DiffuDict toolbox implemented in GeoDict. In the intermediate diffusion regime (Knudsen number Kn ≅ 1), the Bosanquet approximation [111] was used as the sum of two parallel diffusion resistances, the Knudsen and Laplace diffusion:

(2) $$D_{\mathrm{Bosanquet}}={\left(D_{\mathrm{Knudsen}}^{-1}+D_{\mathrm{Laplace}}^{-1}\right)}^{-1}$$

The effective diffusivity was determined with the relative diffusivity *D*_rel_ and the diffusion of oxygen in water (*D*_0@80°C,atm_ = 5.31×10^−5^ cm^2^ s^−1]^ [112]). Furthermore, the geometric tortuosity was obtained according to the following definition [113]:

(3) $$\kappa =\tau^{2},\;\;\;\;\;with\;\kappa =\phi /D_{\mathrm{rel}}$$

where *κ* is the tortuosity factor, *τ* is the geometric tortuosity, and *ϕ* is the porosity.

The conductivity was calculated using Ohm's law and the Poisson equation. The electrical bulk conductivity of titanium was taken as *σ*_0,Ti_ = 2.38⋅10^6^ S m^−1^ [97]. The electric conductivity of IrO_2_ nanoparticles was assumed as *σ*_0,IrO2_ = 2.59⋅10^3^ S m^−1^ [98]. The proton conductivity of the ionomer binder in the electrode was supposed to be the same as the bulk conductivity of Nafion of *σ*_0,Nafion_ = 0.095⋅10^3^ S m^−1^ [84, 114, 115]. The thermal conductivity was solved with Fourier's law. The thermal conductivities were obtained from literature, with 21.9 W m^−1^ K^−1^ for titanium [116], 0.667 W m^−1^ K^−1^ for water at 80°C [117], 0.030 W m^−1^ K^−1^ for oxygen at 80°C [118], and 5.0 W m^−1^ K^−1^ for iridium oxide [78].

### 4.8 Transport Parameters PTE

The transport parameters of the PTE were determined in two ways. In the first approximation, the Micro‐CT tomogram was used for the transport simulations. A cuboid volume was necessary for the calculations. The edges of the PTE cannot be identified straightforwardly due to partially cut fibers and droplets of CL between fibers at the boundaries of the field of view. Therefore, two cuboid volumes were cropped with different solid volume fractions as starting criteria for the PTE edges, one with a solid volume threshold 5% and one with 49.19% as the mean solid volume fraction of the PTL bulk.

In the second approximation, a through‐plane dependent transport tensor was determined. The catalyst distribution in the PTE was known from our previous publication [47], which allows the homogeneous addition of this catalyst amount around uncoated titanium fibers as an approximation. Therefore, 10 arbitrary subvolumes of the PTL with a side length of 100 voxels were chosen, with a porosity of 50.81 % ± 0.50 %. Afterward, the volume was scaled up from a voxel size of 2.5 µm to 0.15625 µm without smoothing, using the “grow strictly” function in GeoDict (equals a grow factor of 2^4^ = 16) to allow small incremental steps of adding catalyst to the fiber by dilation (Figure S25). In total, catalyst‐coated fibers up to 6 dilated voxels were created to reach the maximum solid volume fraction of around 56% determined in our previous publication [47]. The added CL voxels were later considered either solid or porous. In the porous case, the effective transport parameters of the CL were taken without considering the binder that had been previously calculated.

Furthermore, the correlation between fiber diameter size and the difference in transport parameters between solid and porous CLs was examined. First, similar to the above‐described procedure, 5 arbitrary subvolumes of the PTL with a side length of 150 voxels were selected within the same porosity range. Afterward, the voxel size of the titanium fiber subvolumes was changed from 2.5 to 1.25 µm and 0.675 µm to receive half‐size fibers and quarter‐size fibers without affecting the porosity of the sample and the number of voxels. Next, the datasets were scaled up from 1.25 and 0.675 µm to 0.15625 µm without smoothing, using the “grow strictly” function in GeoDict (equals a grow factor of 2^3^ = 8/2^2^ = 4). Hence, all subvolumes had the same voxel size, which allowed a dilation by 6 voxels to add the same CL thickness to each subvolume. Finally, the mean permeability values between solid and porous CL were compared in dependence on the fiber thickness.

### 4.9 Binder Modeling

The binder was not visible in the FIB‐SEMt reconstruction of the anode, which was due to the limited resolution and the sensitivity of the binder to beam damage from ion beam milling and electron microscopy. Hence, the binder was modeled as a layer covering the catalyst particles. Two model cases were considered, both with the constraint that the binder covers the catalyst particles homogeneously. The first scenario assumed that no binder was detected in the tomography due to beam damage. For that reason, the binder was added via a morphological dilation of the segmented grains. The second scenario supposed that the ionomer binder was detected and surrounded by the catalyst, but could not be segmented individually due to limits in resolution. This case was implemented via a morphological erosion, where the eroded areas were then assigned as a binder.

As a reference point for the binder modeling, the volume ratio between the catalyst particles and the binder was based on TGA data of the CL, performed in our previous study [47], and the densities of the components of the CL:

(4) $$\frac{V_{\mathrm{IrO}2}}{V_{\mathrm{binder}}}=\frac{\rho_{\mathrm{Nafion}}\cdot w_{\mathrm{IrO}2}}{\rho_{\mathrm{IrO}2}\cdot w_{\mathrm{Nafion}}}=1.47$$

where *ρ*_IrO2_ = 11.66 g cm^−3^ and *ρ*_Nafion_ = 2.05 g cm^−3^ [19] are the densities, and *w*_IrO2_ = 89.29% [47] and *w*_Nafion_ = 10.71% [47] are the mass fractions of the CL components.

The binder volume fraction under wet conditions was calculated by multiplying the binder volume fraction by the swelling factor of 1.8 [52, 69], which was implemented by dilating the binder phase. Since dilation operations can only be performed with integers as factors, the dilation was performed twice for the two closest values of the swelling factor of 1.8. The transport parameters were then linearly interpolated between the two resulting structures.

Depending on the above‐described scenario, two different *V*_IrO2_ contents, “all binder segmented” and “no binder segmented”, that matched the volume ratio of Equation (4), and the related IrO_2_ volume fraction *V*_IrO2_/*V*_total_ were determined. Under the assumption that the catalyst structure and the CL thickness would remain unchanged with varying binder content, which was in accordance with PEMWE and PEMFC literature [85, 86, 87], the solid volume fraction SVF*_x_*_wt.%_ of a CL with an arbitrary binder content x wt.% can be determined for both scenarios:

(5) $$\begin{array}{ccc} & & \mathrm{SV}F_{x\;\mathrm{wt}.\%}=\frac{V_{\mathrm{IrO}2}+V_{x\;\mathrm{wt}.\%\;\mathrm{binder}}}{V_{\mathrm{total}}}=\frac{V_{\mathrm{IrO}2}}{V_{\mathrm{total}}}\left(1+\frac{1}{y}\right);\\ & & \mathrm{with}\;\;y=\frac{V_{\mathrm{IrO}2}}{V_{x\;\mathrm{wt}.\%\;\mathrm{binder}}}\;\;\mathrm{and}\;\;V_{\mathrm{total}}=V_{\mathrm{IrO}2}+V_{x\;\mathrm{wt}.\%\;\mathrm{binder}}+V_{\mathrm{pore}} \end{array}$$

The volume ratio *V*_IrO2_/*V*_total_ stayed constant for the respective scenario. The volume ratio *y* between the catalyst particles and the binder was calculated with Equation (4) depending on the binder mass fraction *w*_Binder_. The binder volume fraction and the porosity were determined based on the SVF*_x_*_wt.%_ and the catalyst volume ratio *V*_IrO2_/*V*_total_.

Furthermore, the binder was modeled around the reconstructed CL volume with the “Add Binder” function from GeoDict as an alternative modeling approach. This function adds binder by placing material in the form of a concave meniscus at locations where the surfaces of the structure were close together. Therefore, an isotropic distribution of the binder was assumed, along with a contact angle of 0°. The added solid volume percentage of binder was matched roughly with the solid volume percentage of binder added via the dilation operation.

## Funding

Bundesministerium für Bildung und Forschung, 03SF0666A; Bundesministerium für Wirtschaft und Energie 03EI3029A.

## Conflicts of Interest

The authors declare no conflicts of interest.

## Supporting information




**Supporting File**: smll72868‐sup‐0001‐SuppMat.docx.

## Data Availability

The data that support the findings of this study are available from the corresponding author upon reasonable request.
