# Supplementary material for: Laying the Foundation for Digital Material Design of Porous Transport Electrodes for PEM Water Electrolysis With Multiscale Tomography
Source: Small. 2026 Feb 18;22(22):e13029. doi: 10.1002/smll.202513029 (PMC13089096; doi:10.1002/smll.202513029)
Supplement: Supplementary file 1 — Supporting File: smll72868‐sup‐0001‐SuppMat.docx. [file SMLL-22-e13029-s001.docx]

Supporting Information

**Laying the foundation for digital material design of porous transport electrodes for PEM water electrolysis with multiscale tomography**

Markus Bierling, David McLaughlin, Andreas Hutzler, Mingjian Wu, Darius Hoffmeister, Dennis Chalupczok, Erdmann Spiecker, Thomas Böhm, Simon Thiele

**Overview CCM vs. CCS**

**
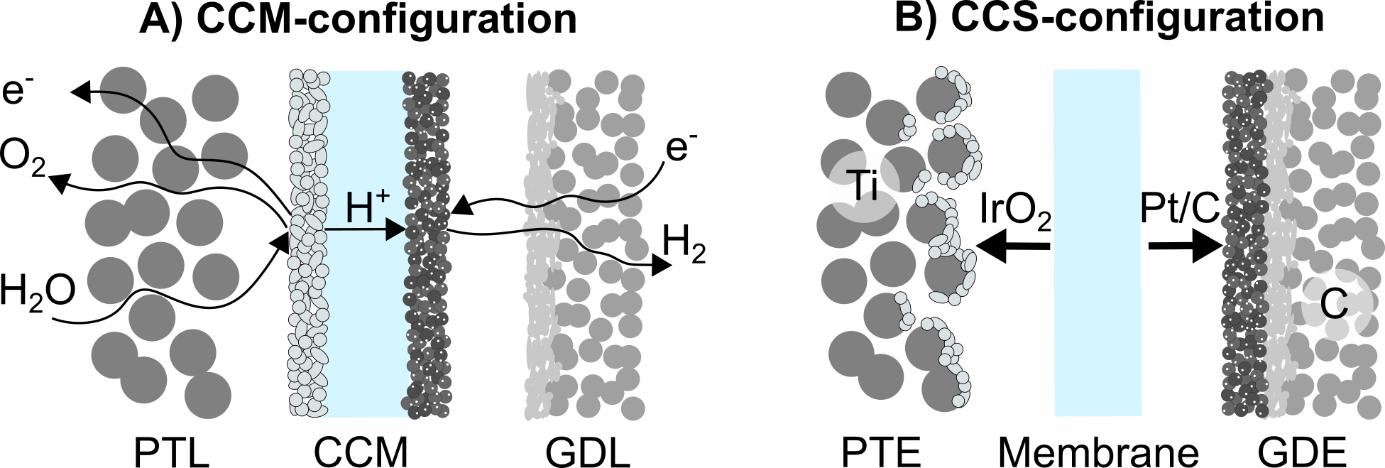
**

Figure 1 Illustration of the difference between A) a catalyst-coated membrane (CCM) and B) a catalyst-coated substrate (CCS) configuration. CCS on the cathode side is referred to as a gas diffusion electrode (GDE), and the anodic counterpart is called a porous transport electrode (PTE).

**General information on multiscale tomography**


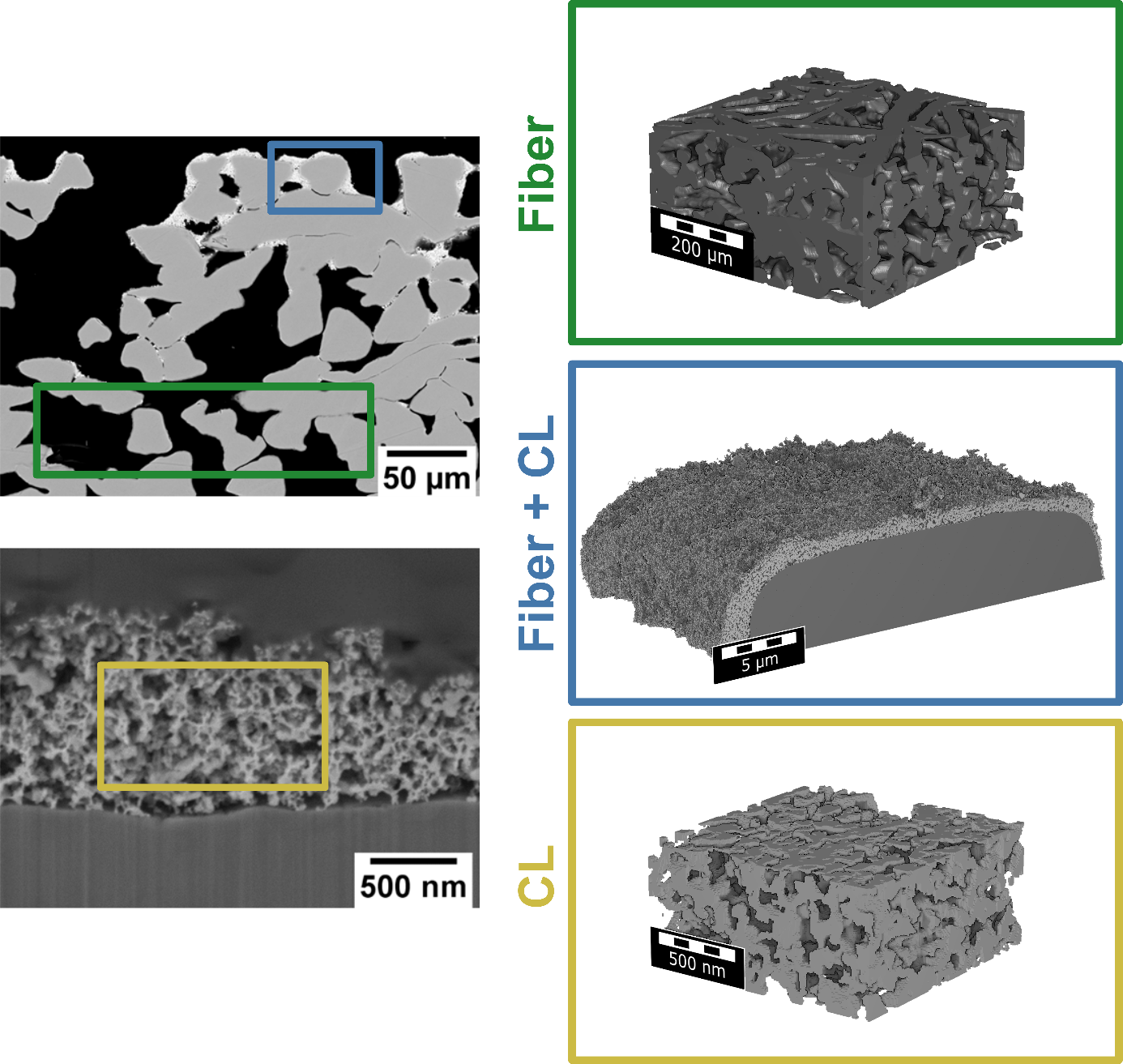


Figure 2 Overview of the multiscale nature of anodic PTEs in PEMWE. Tomograms of the titanium fiber, the catalyst-coated fiber, and the CL are shown. Micro-CT data of the titanium fibers segmentation were taken from our previous publication.^[1]^


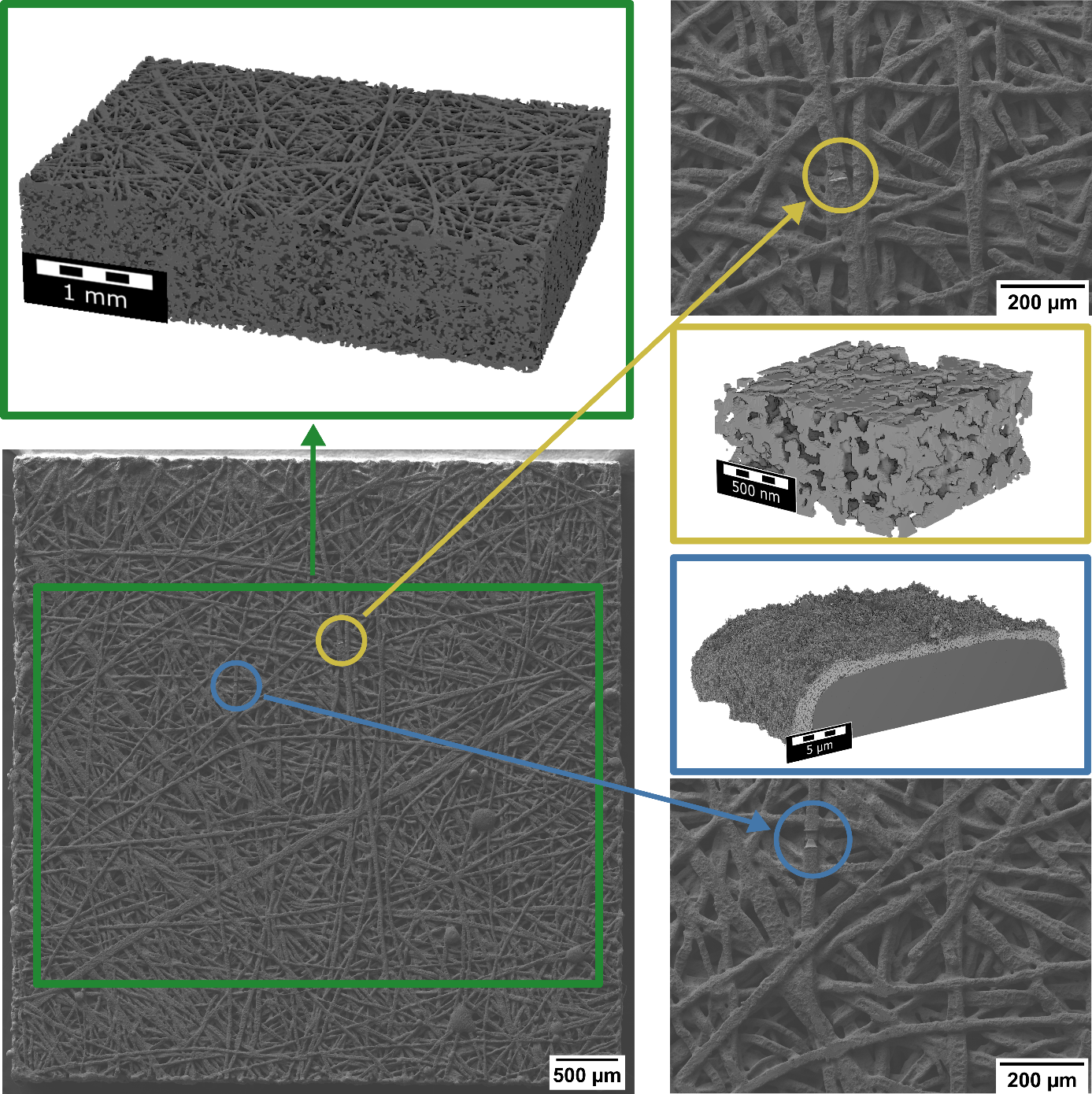


Figure 3 Illustration of the identical location tomography. All FIB-SEMts (yellow and blue) were in the same sample area as the Micro-CT (green). SEM surface images illustrate the exact locations of the tomographies. Micro-CT data were adapted from our previous publication.^[1]^

**CL morphology**


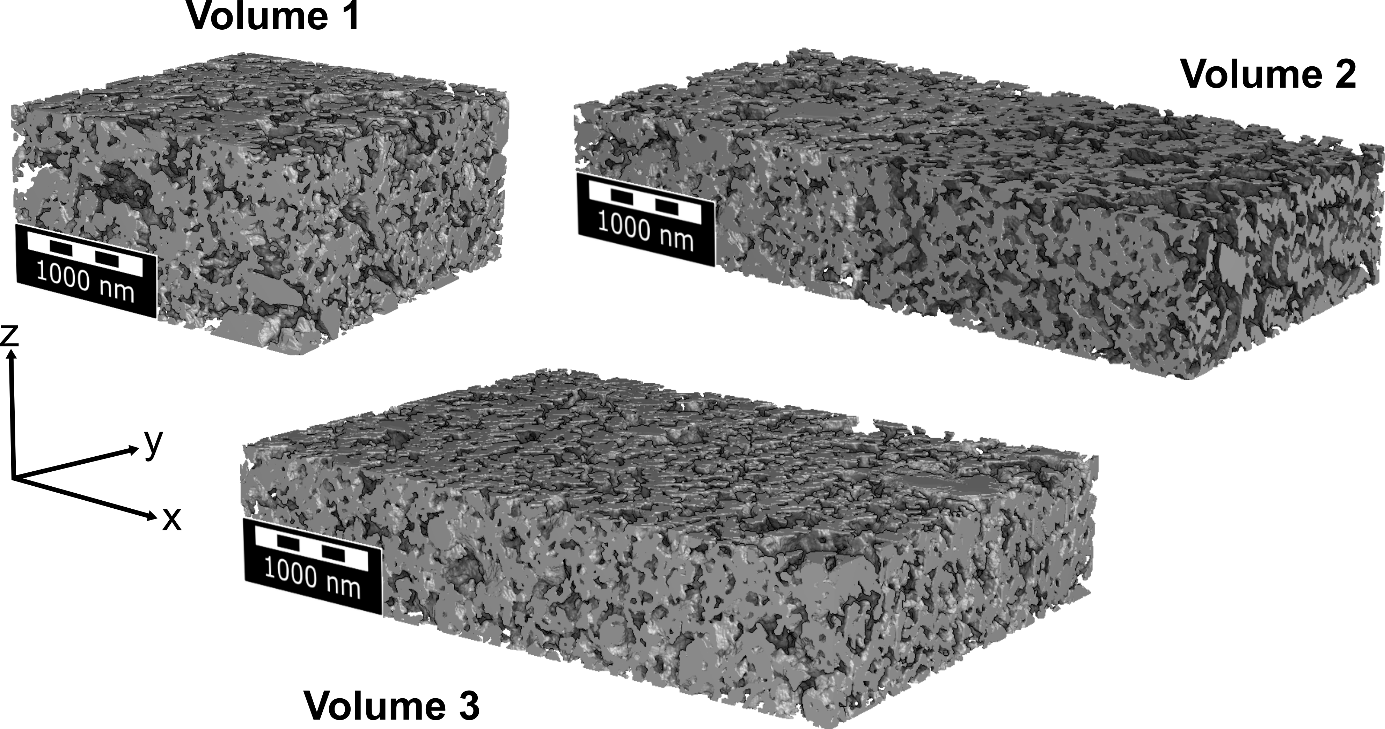


Figure 4 Three subvolumes for CL segmentation. Taking larger areas (volume 2 and volume 3) comes with the drawback of a lower CL thickness in comparison to volume 1 due to inhomogeneous coating thickness.


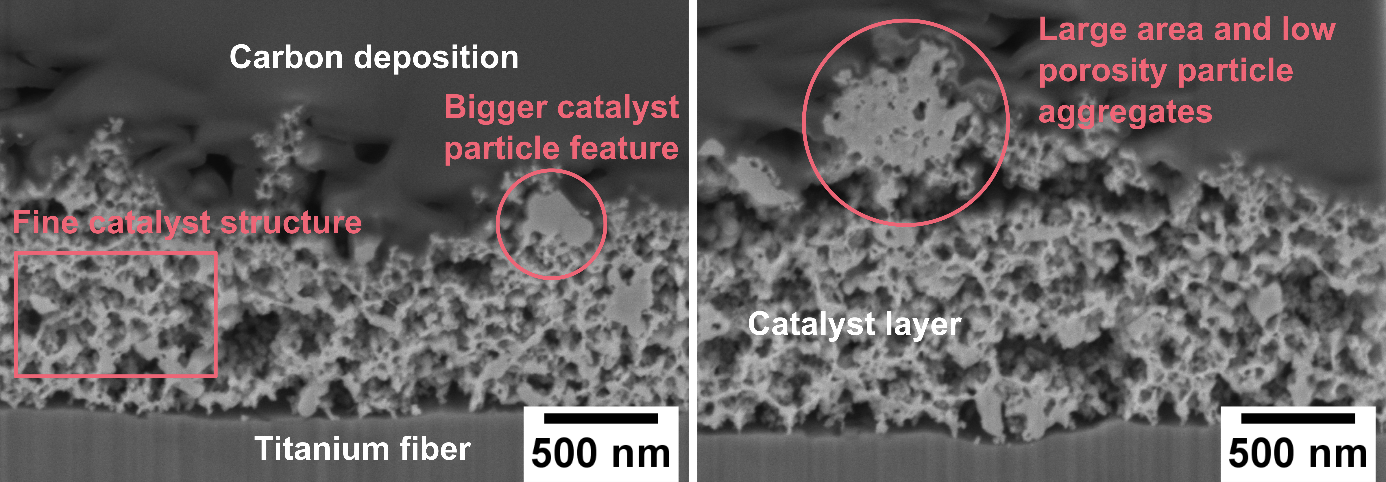


Figure 5 Features of the CL can be divided into a fine catalyst structure, bigger catalyst particle features, and large areas with low porosity particle aggregates.


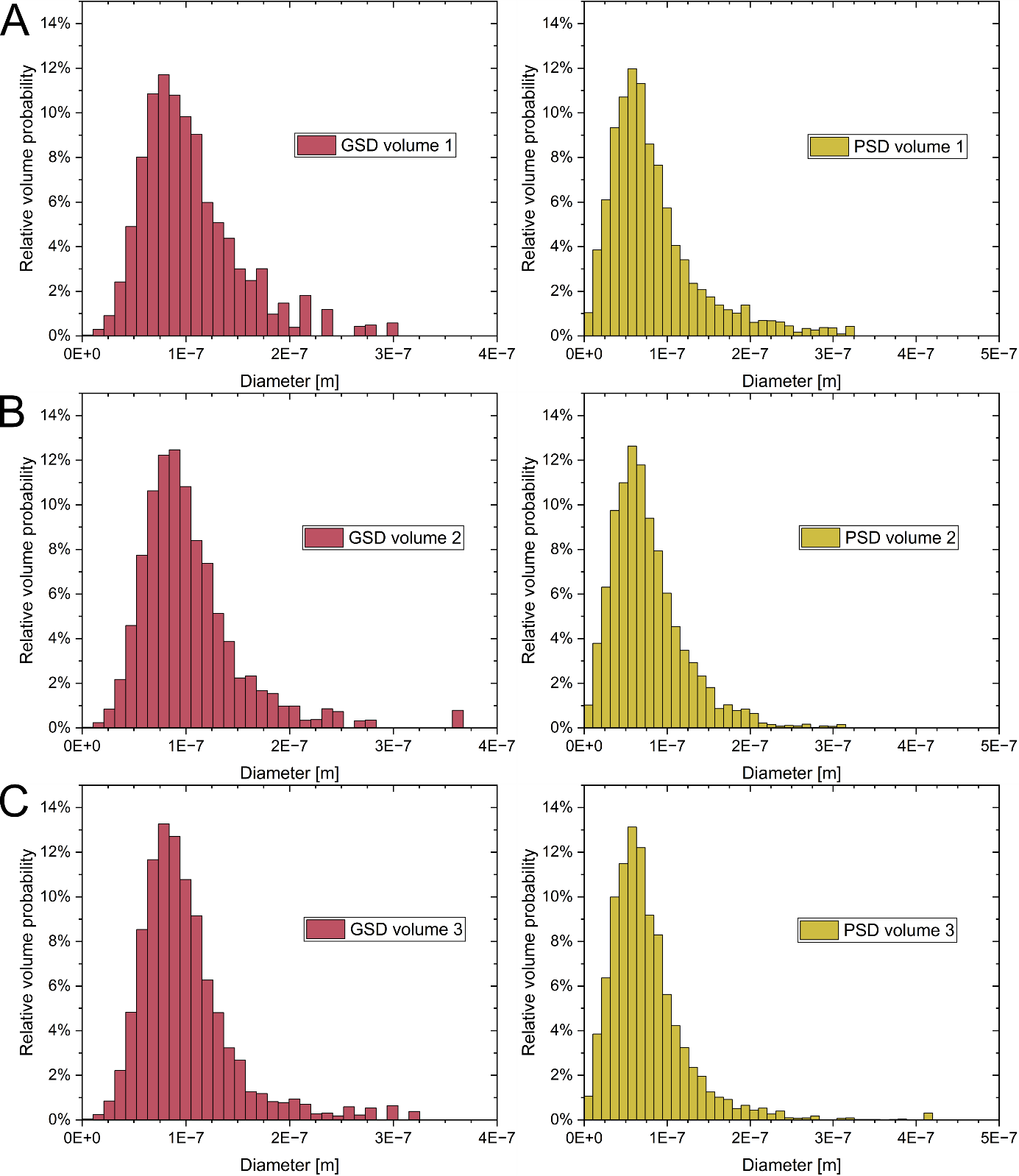


Figure 6 Grain and pore size distributions of the individual volumes. A) Volume 1 has a mean grain diameter of 104.6 nm, a median grain diameter of 94.6 nm, a mean pore diameter of 84.0 nm, and a median pore diameter of 69.7 nm. B) Volume 2 has a mean grain diameter of 103.7 nm, a median grain diameter of 93.8 nm, a mean pore diameter of 76.9 nm, and a median pore diameter of 67.8 nm. C) Volume 3 has a mean grain diameter of 100.3 nm, a median grain diameter of 90.9 nm, a mean pore diameter of 77.0 nm, and a median pore diameter of 66.2 nm.


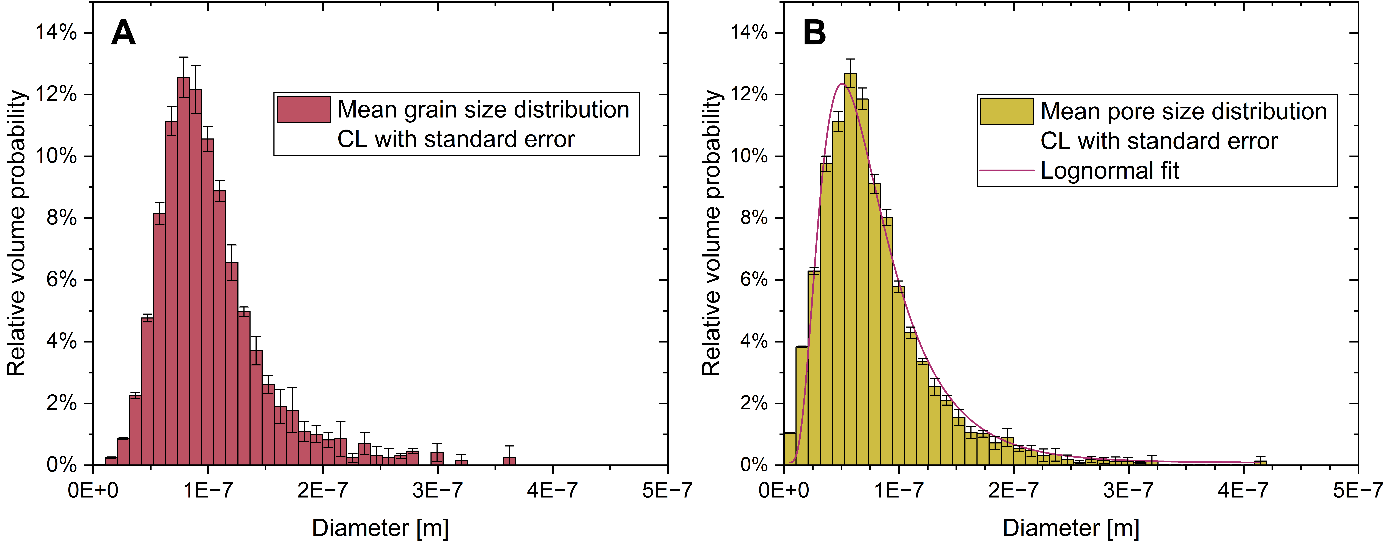


Figure 7 A) Mean grain and B) mean pore size distribution with standard deviation of the three segmented subvolumes. The PSD followed a lognormal distribution (R^2^-value of 97.92%).


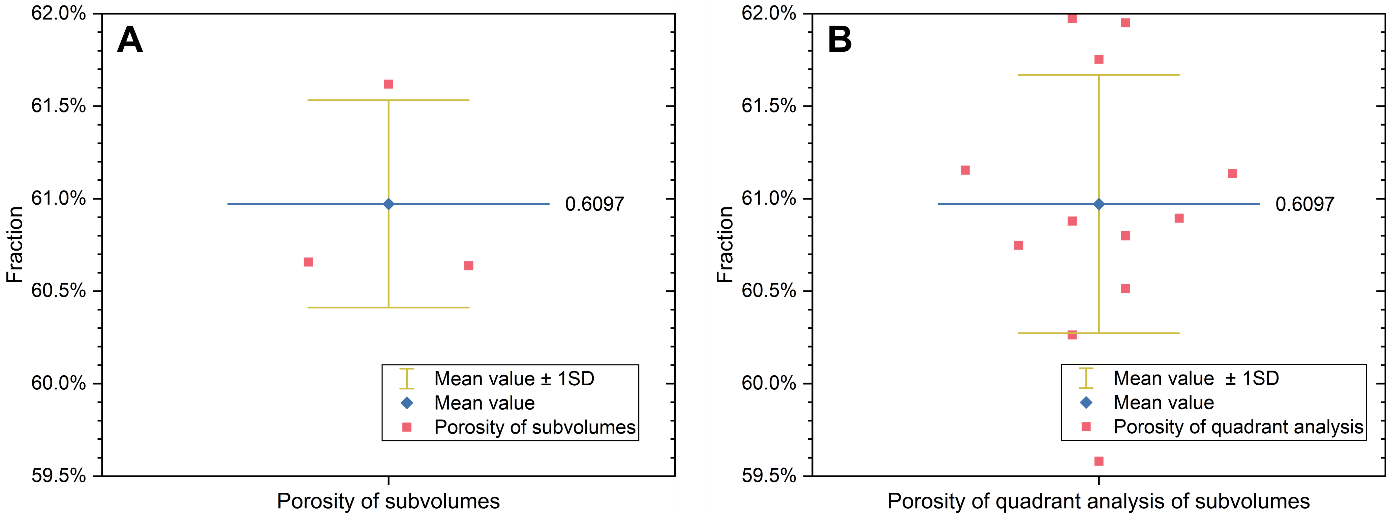


Figure 8 Representative volume analysis via porosity and quadrant analysis of the subvolumes according to McLaughlin et al.^[2]^ The representative character of the subvolumes is confirmed by the low standard deviation of the porosity (± 0.56 % around a mean of 60.97 %), which equals a coefficient of variation (ratio of standard deviation and mean value) of 0.92 % (A). B) shows the quadrant analysis of the subvolumes, with 60.97 % ± 0.70 % (mean ± SD) and a coefficient of variation of 1.15 %.

**BET analysis**

By bringing the BET equation into a linear expression, the slope $\frac{c-1}{v_{m}c}$ and intersect $\frac{1}{v_{m}c}$ can be used to determine the absorbed gas volume needed to form a monolayer *v*_m_ (see Figure S9):

| $\frac{1}{v\left( \frac{p_{0}}{p}-1 \right)}=\frac{c-1}{v_{m}c}\left( \frac{p}{p_{0}} \right)+\frac{1}{v_{m}c}$ | (1) |
| --- | --- |
|  |  |

Where *v* is the adsorbed gas quantity (see Figure S9), *p*_0_ the saturation pressure of the adsorbate, *p* the equilibrium pressure of the adsorbate, and *c* the BET constant.

The specific surface area can then be calculated from the monolayer absorbed gas volume *v*_m_, the Avogadro number *N*_A_, the cross-sectional area of an adsorbed nitrogen molecule *s*, the molar volume of the adsorbed gas *V,* and the mass of the sample *m*:

| $S_{\mathrm{BET}}=\frac{v_{m}N_{A}s}{Vm}$ | (2) |
| --- | --- |

Furthermore, the mean diameter *d*_BET_ can be estimated via the specific surface area assuming the catalyst consists of *n* homogeneous and spherical particles with radius *r* and density *ρ*_IrO2_ = 11.7 g cm^-3^:

| $S_{\mathrm{BET}}=\frac{nS_{IrO2 particle}}{m_{IrO2 particles}}=\frac{nS_{IrO2 particle}}{{n\rho_{IrO2}V}_{IrO2 particles}}=\frac{n4\pi r^{2}}{n\rho_{IrO2}4/3{\pi r}^{3}}$ | (3) |
| --- | --- |
|  |  |
| $d_{\mathrm{BET}}=2r= \frac{6}{\rho S_{\mathrm{BET}}}=17.87 \mathrm{nm}$ | (4) |


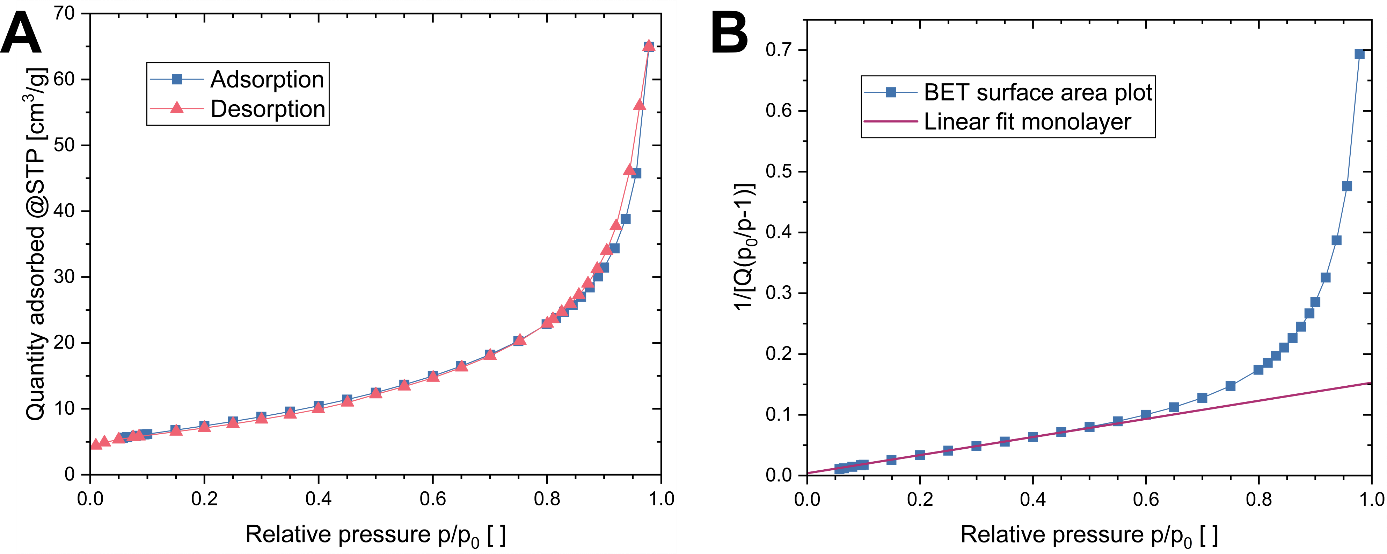


Figure 9 A) Isotherm plot of the adsorption and desorption process. B) BET surface area plot with linear fit between (0.05 and 0.35 p/p_0_).

**Pristine catalyst particles**


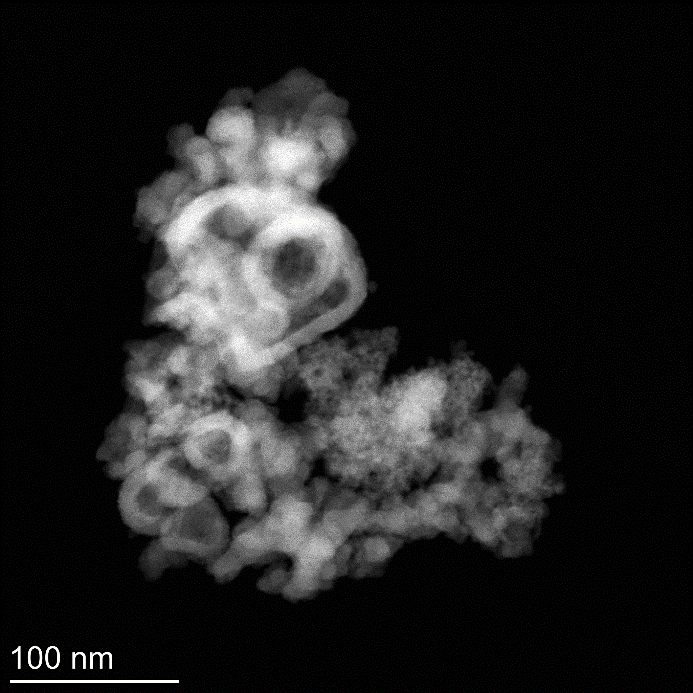


Figure 10 HAADF-STEM image of pristine catalyst powder.

**Catalyst-coated titanium fiber**


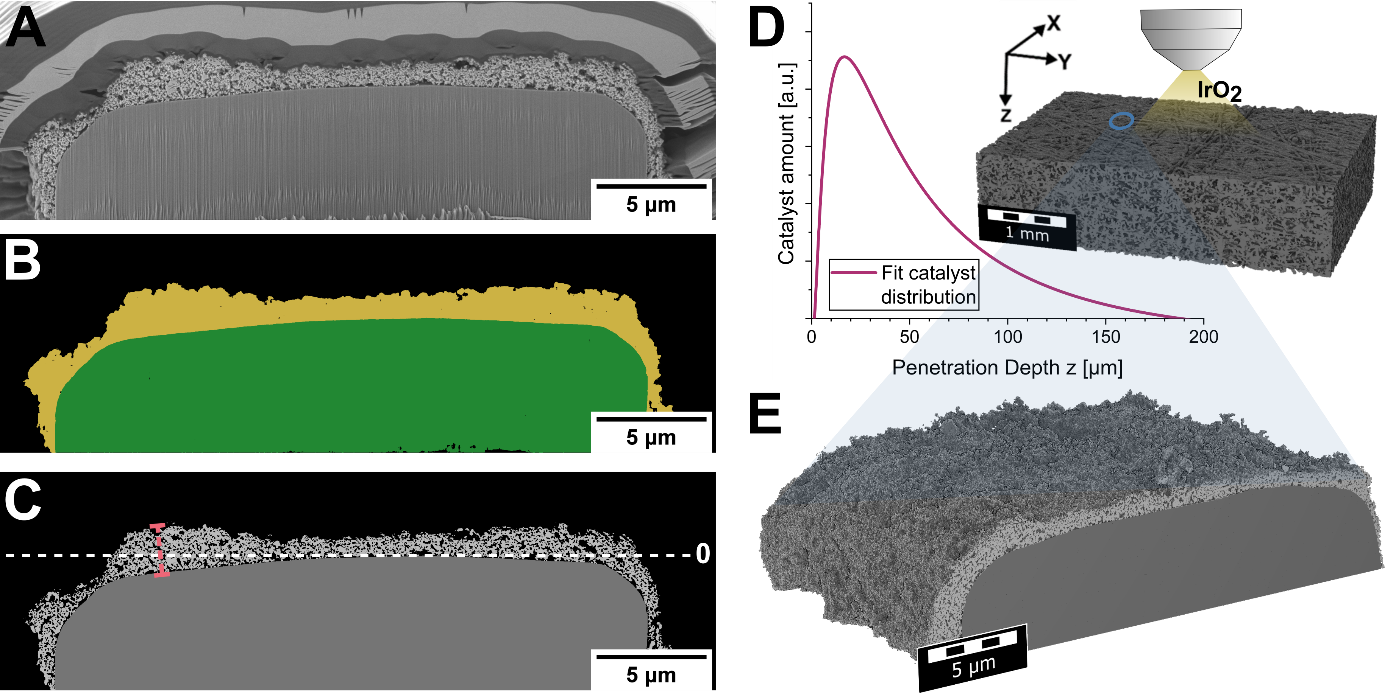


Figure 11 Overview of the tomogram of a catalyst-coated PTL fiber. A) Raw data slice of the FIB-SEMt; B) First segmentation and differentiation between pore, fiber, and CL via an AI algorithm; C) Segmentation of the catalyst layer after post-processing and Otsu-thresholding. 0-line (z=0) is defined as the highest fiber edge point. D) Illustration of the catalyst coating process with catalyst distribution fit vs. penetration depth. Data taken and adapted from our previous publication^[1]^. E) Final reconstructed catalyst-coated fiber volume.


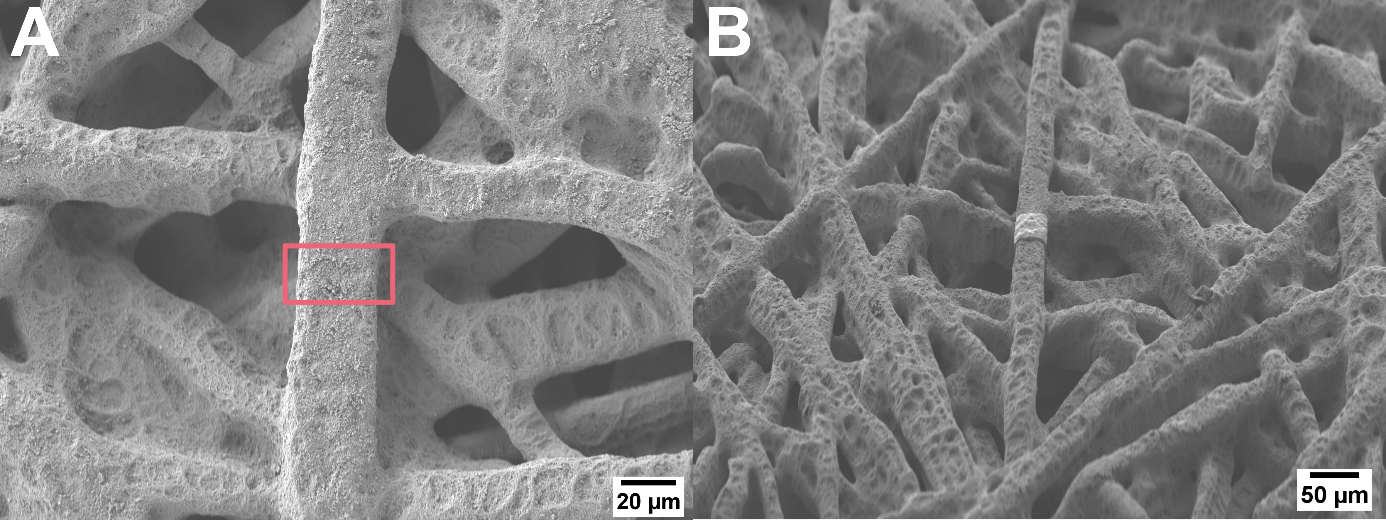


Figure 12 A) Area of fiber-catalyst-layer tomography (red rectangle). B) Deposited Pt coating of the tomography area.


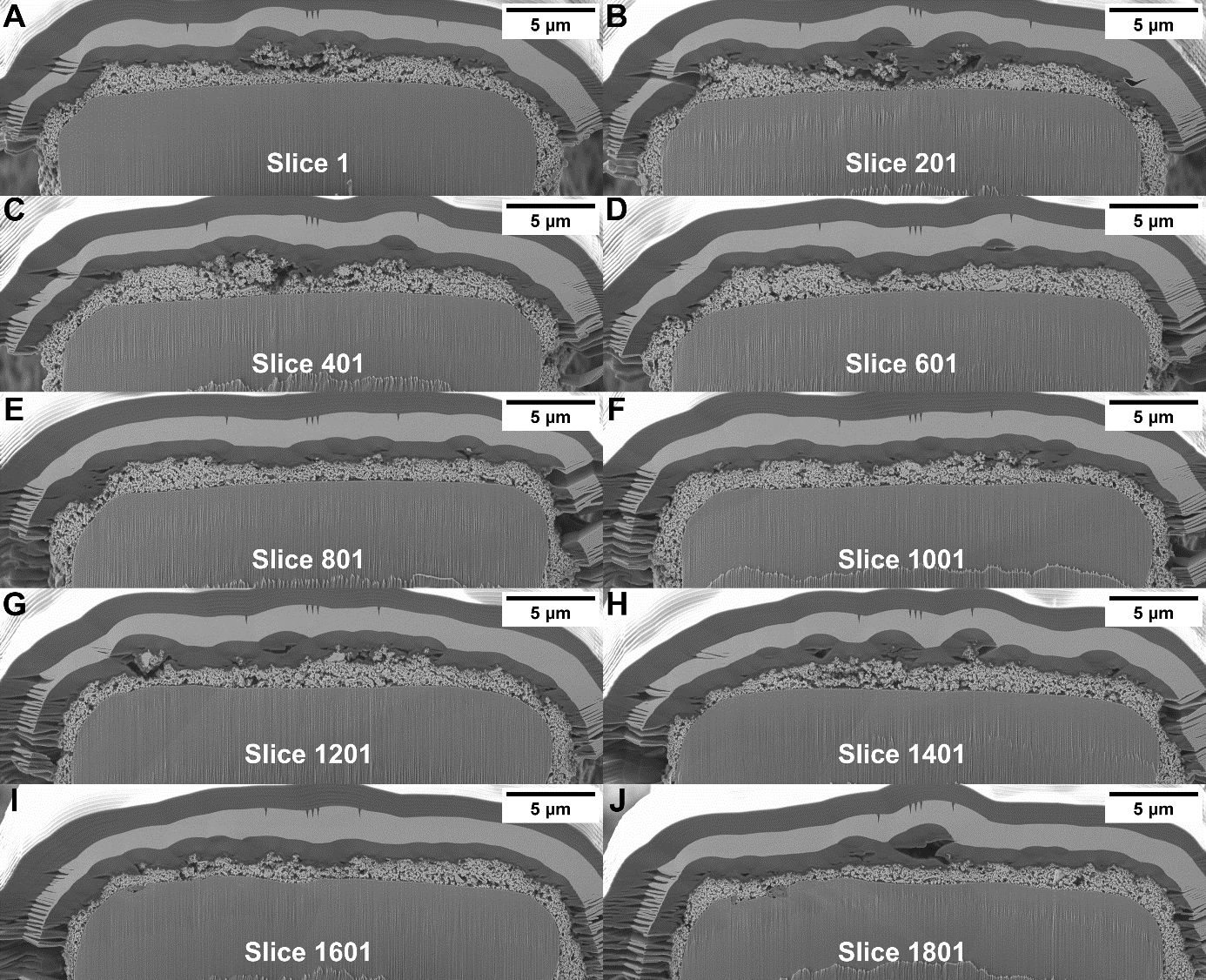


Figure 13 Raw SEM images of the tomography data set of the single fiber. Here, a sequence of images with an interval of 200 slices is displayed to give an impression of a single spray-coated fiber and its variance in the catalyst layer morphology.


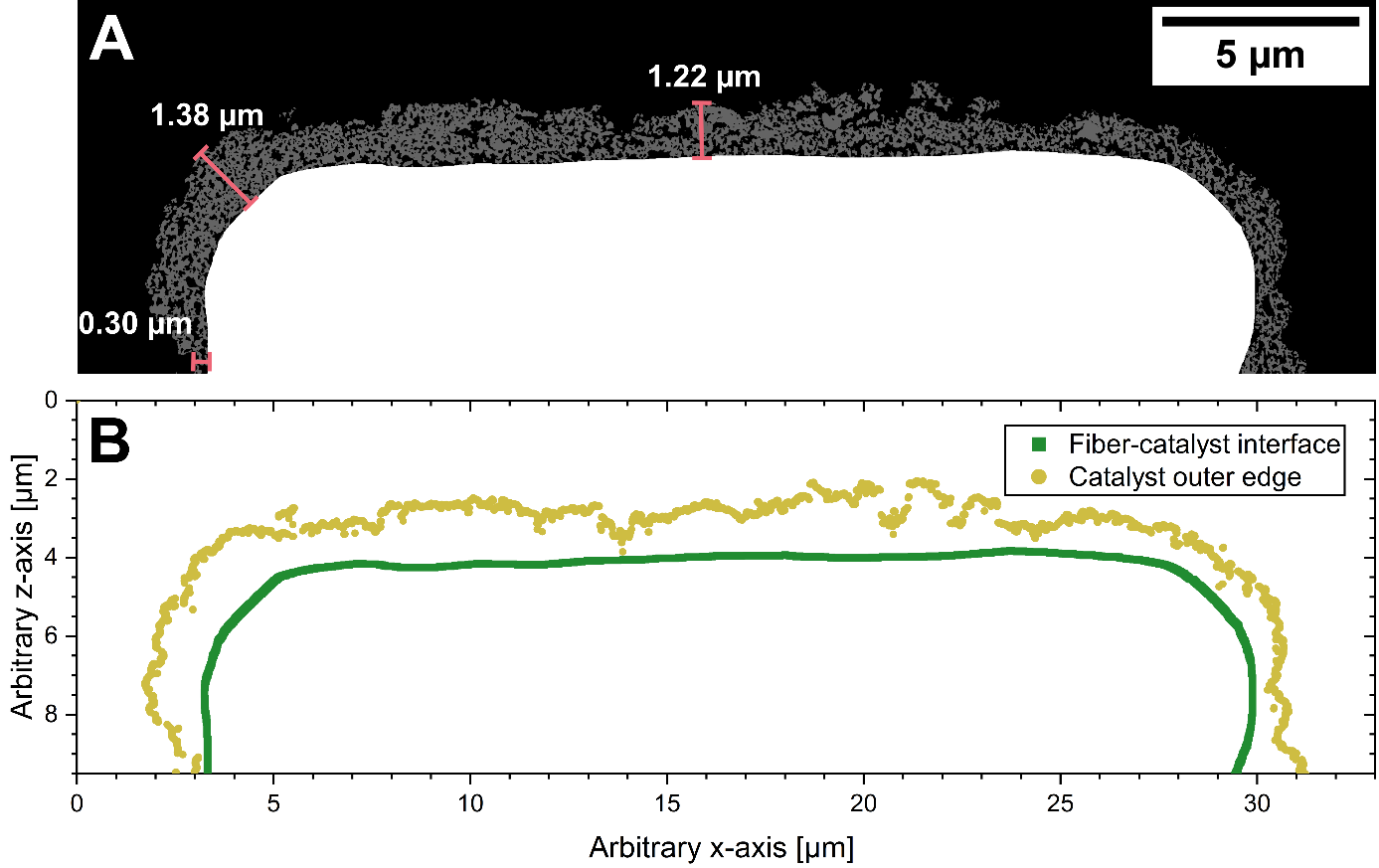


Figure 14 A) Illustration of the fiber-catalyst segmentation with several catalyst layer thicknesses. B) An exemplary analysis of a single slice includes the detection of the fiber-catalyst interface (green) and the outer edge of the catalyst layer.


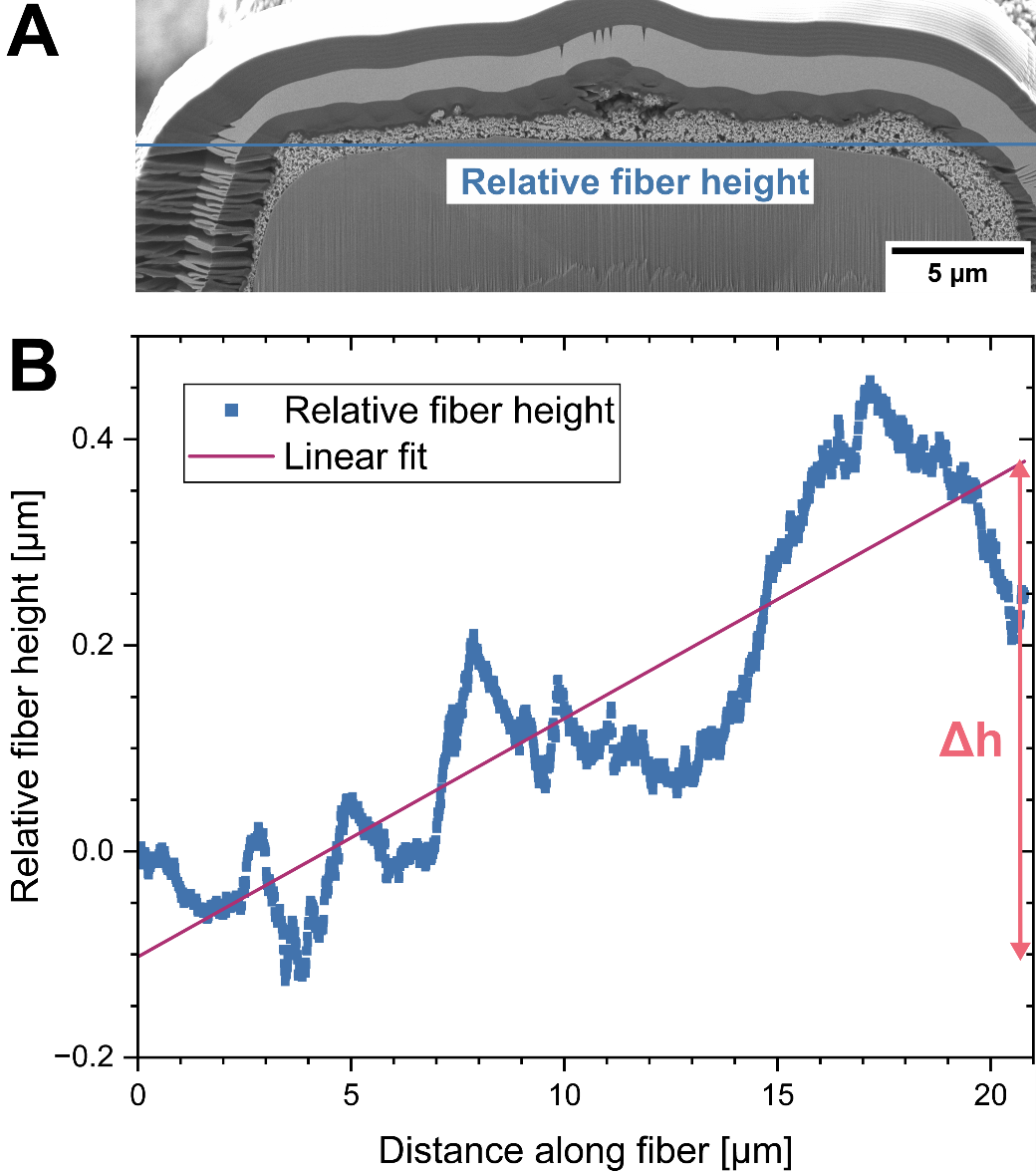


Figure 15 A) Illustration of the definition of the relative fiber height as a mean value of 1000 highest fiber edge points. B) Trend of the relative fiber height along the fiber. The linear fit suggests a small positive fiber slope along the imaged volume.


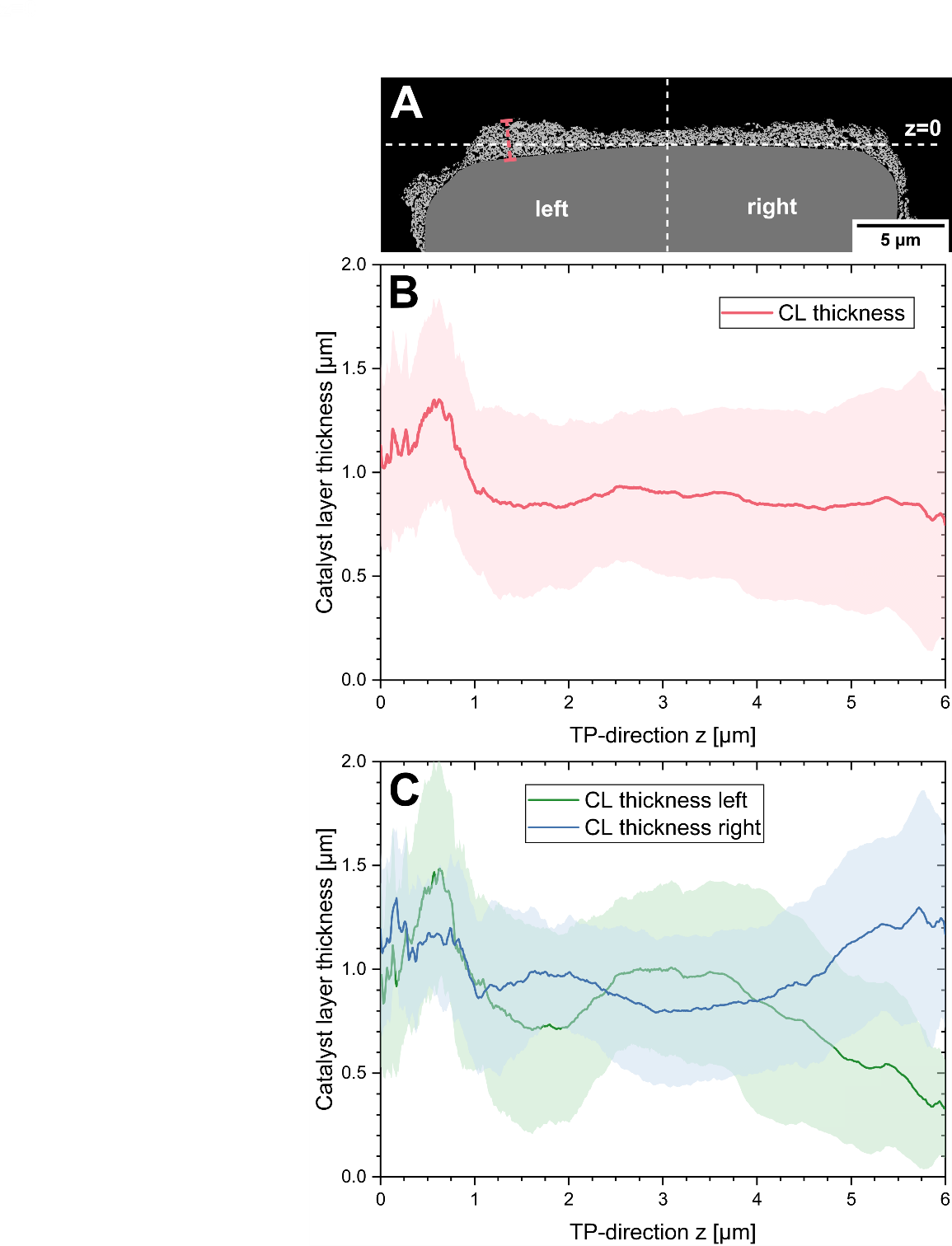


Figure 16 Catalyst layer thickness distribution in dependence on TP-direction z. A) Sketch to differentiate between the left and right fiber side and definition of z=0 as the highest fiber point. TP-direction z was ended at 6 µm (criterion of more than 1000 slides) B) Mean CL thickness in dependency of z. C) Differentiation between left and right CL thickness.

**Surface roughness catalyst layer**

The following definitions try to quantify the surface roughness of the catalyst layer spray-coated onto the fiber. The definitions are based on the surface roughness calculations of our previous paper ^[1]^ and Schuler *et al.* ^[3]^

- Mean thickness $t_{m}^{\mathrm{CL}}$

| $t_{m}^{\mathrm{CL}}=\frac{1}{\sum_{i=0}^{M-1} N_{i}}\sum_{i=0}^{M-1} \sum_{j=0}^{N_{i}-1} t_{ij}^{\mathrm{CL}}$ | (5) |
| --- | --- |

- Arithmetic mean surface roughness $R_{a}^{\mathrm{CL}}$

| $R_{a}^{\mathrm{CL}}=\frac{1}{\sum_{i=0}^{M-1} N_{i}}\sum_{i=0}^{M-1} \sum_{j=0}^{N_{i}-1} \vert\Delta t_{ij}\vert$ | (6) |
| --- | --- |
| $\Delta t_{ij}=t_{ij}-t_{m}$ | (7) |

- Root mean square surface roughness $R_{q}^{\mathrm{CL}}$

| $R_{q}^{\mathrm{CL}}=\sqrt{\frac{1}{\sum_{i=0}^{M-1} N_{i}}\sum_{i=0}^{M-1} \sum_{j=0}^{N_{i}-1} (\Delta t_{ij})^{2}}$ | (8) |
| --- | --- |

Where:

- *M* is the number of slices of the tomography, and *N_i_* is the corresponding thickness of slice *i*
- $t_{ij}$ is the total catalyst layer thickness at probing positions *i* and *j*
- $\Delta t_{ij}$ is the mean deviation thickness of each point from the mean thickness

Table 1 Calculated roughness parameters of the total fiber, the top edge part of the fiber, and the side edge part of the fiber.

|  | Total fiber | Top edge | Side edge |
| --- | --- | --- | --- |
| $t_{m}^{CL}$ | 1.047 μm | 1.104 μm | 0.899 μm |
| $R_{a}^{CL}$ | 0.362 μm | 0.366 μm | 0.332 μm |
| $R_{q}^{CL}$ | 0.462 μm | 0.466 μm | 0.417 μm |

**STEM-EDXS measurements of CL**

STEM-EDXS measurements were performed at different locations. The acceleration voltage was either 80 kV or 200 kV. Nafion with the chemical sum formula C_7_HF_13_O_5_S·(C_2_F_4_)_m_ (here m=5.6 for Nafion with EW 1000) was the ionomer of the catalyst layer. Due to the large quantity of Fluor atoms, F atoms were used to identify the binder in the STEM-EDXS measurements. All measurements showed that the catalyst particles are covered with binder. The degree of coverage could not be determined because of the low fluorine signal intensity.


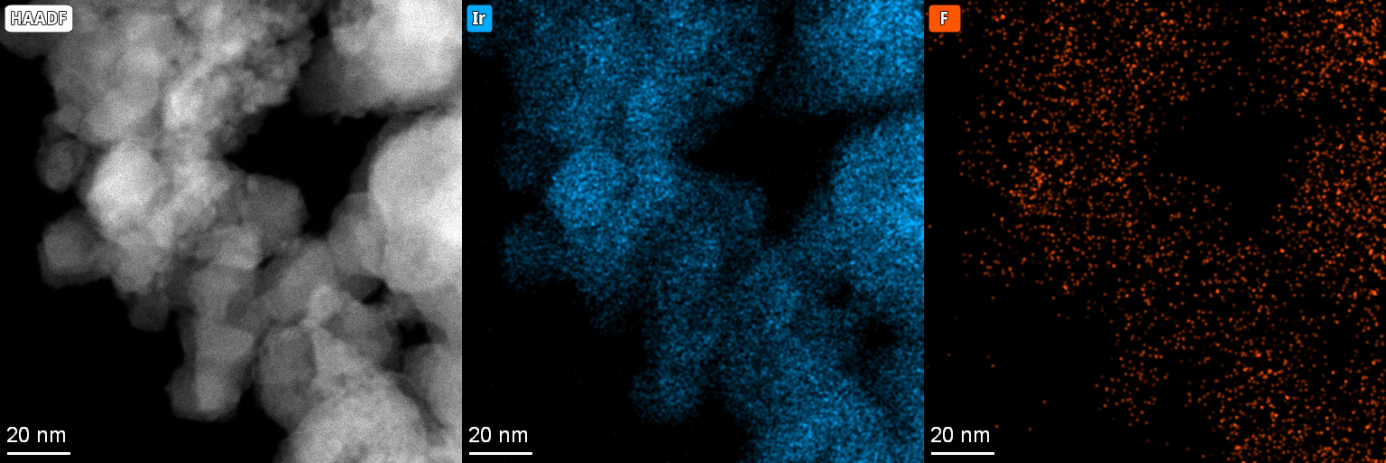


Figure 17 HAADF image of a small fraction of the catalyst layer at 200 kV. Additionally, EDX measurement revealed the location of the catalyst (Ir signal) and ionomer (Fluor signal). The HAADF image displays catalyst agglomerates formed from individual particle grains with a size of around 20 nm.

**STEM-EELS core-loss analysis**

STEM-EELS measurements were performed on the same sample with the expectation of mapping the different components. With the known beam sensitivity in mind, we changed the spatial sampling (from 4.8 nm/pixel to 3.8 nm/pixel to 1.1 nm/pixel) to alter the dosage for core-loss signals, which are expected to be used to identify the components.

The EELS detector uses all the information of the primary electron beam and is, in principle, more suited to track low percentages of light elements than the EDXS detector, which only measures a fraction of the emitted X-ray photons.

Even though the required spatial resolution and dwell time for a good enough signal-to-noise ratio of the core-loss EEL spectra demanded a dose that has caused sample degradation. This phenomenon is known in the literature.^[4,5]^ Melo *et al.*^[4]^ reported that beam damage (mass loss) can already occur at fluences higher than 300 e^−^/nm^2^. The conditions applied there for the core-loss signals were conducted with much higher fluences that were at least a factor of 10 higher (Figure S18). For that reason, the expected F-K signal was not detectable under the current conditions and is attributed to electron beam damage and mass loss. Therefore, the discussions here are concentrated at the C-K signal region (Figure S18). The loss of mass at higher dose can be seen from the lower carbon signals at higher dosage.


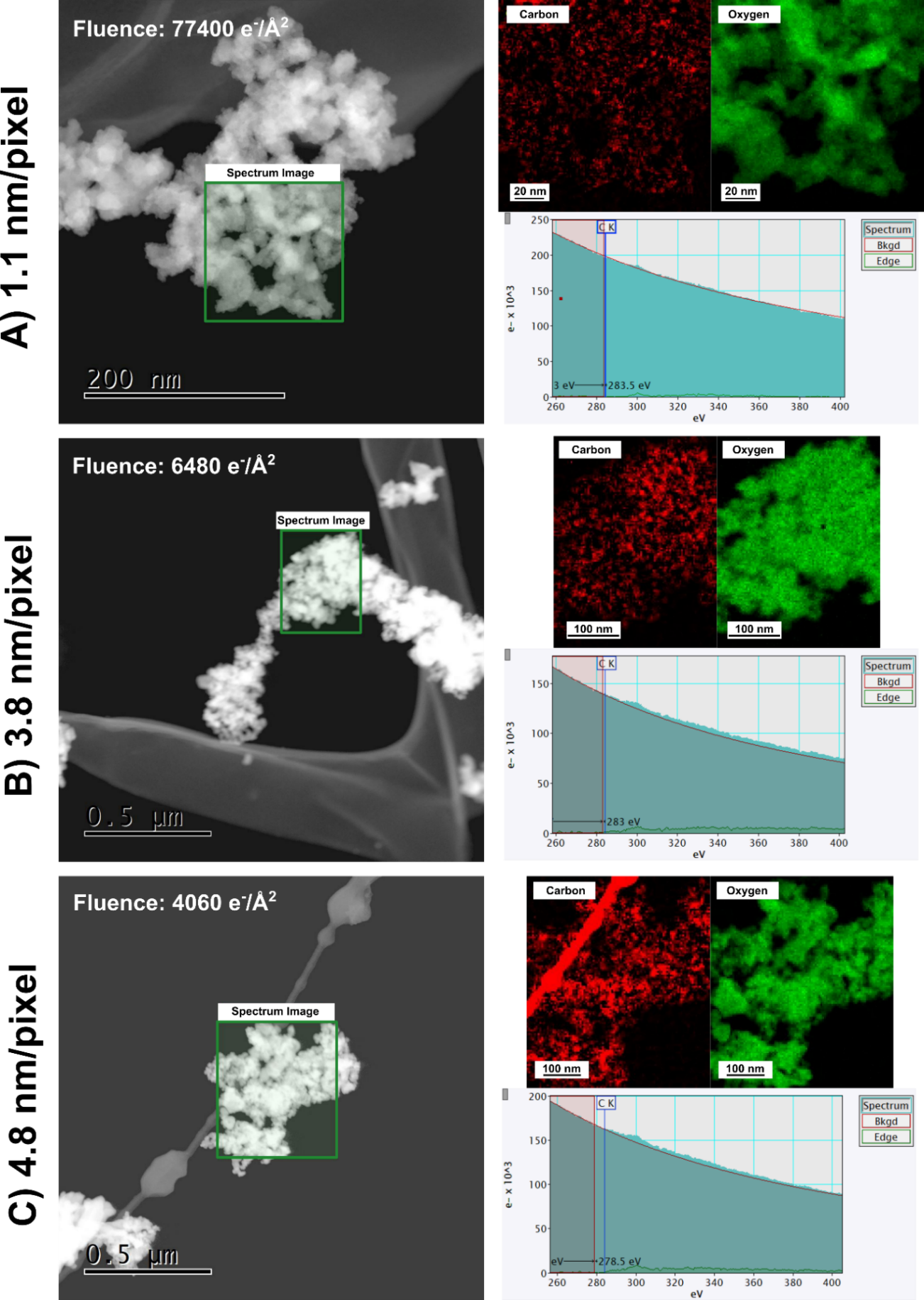


Figure 18 STEM-EELS measurements at constant probe current of 15 pA and pixel dwell time of 10 ms with different instantaneous dosage via changing the spatial sampling A) 1.1 nm/pixel and fluence of 77400 e^-^/Å^2^, B) 3.8 nm/pixel and fluence of 6480 e^-^/Å^2^, and C) 4.8 nm/pixel and fluence of 4060 e^-^/Å^2^ . For each dataset, the sum signal and the elemental maps of Carbon and Oxygen are evaluated using model-based fitting. In all cases, F-K signal at ~685 eV could not be identified. The loss of mass at higher dose can be seen from the lower carbon signals at higher dosage.

**
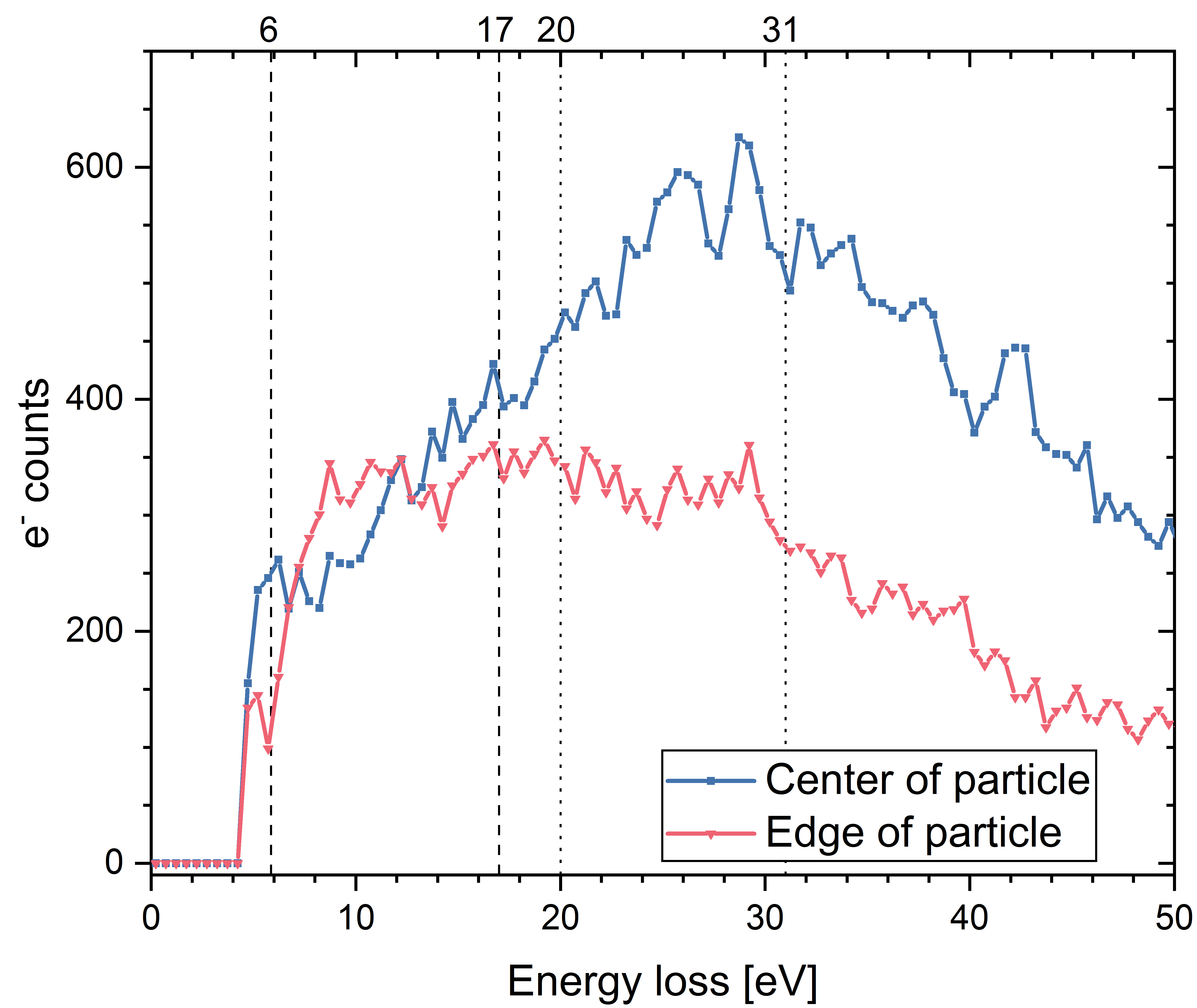
**

Figure 19 Single pixel low-loss EEL spectra (zero-loss removed) extracted from the red and blue dots in Figure 4B and 4C of the main manuscript, respectively. The dashed lines highlight the 6 – 17 eV interval that includes the characteristic Nafion low-loss features. The dotted lines represent the 20-31 eV interval that includes the bulk plasmon of Ir / IrOx in the low-loss range.

**Binder modeling in the CL**


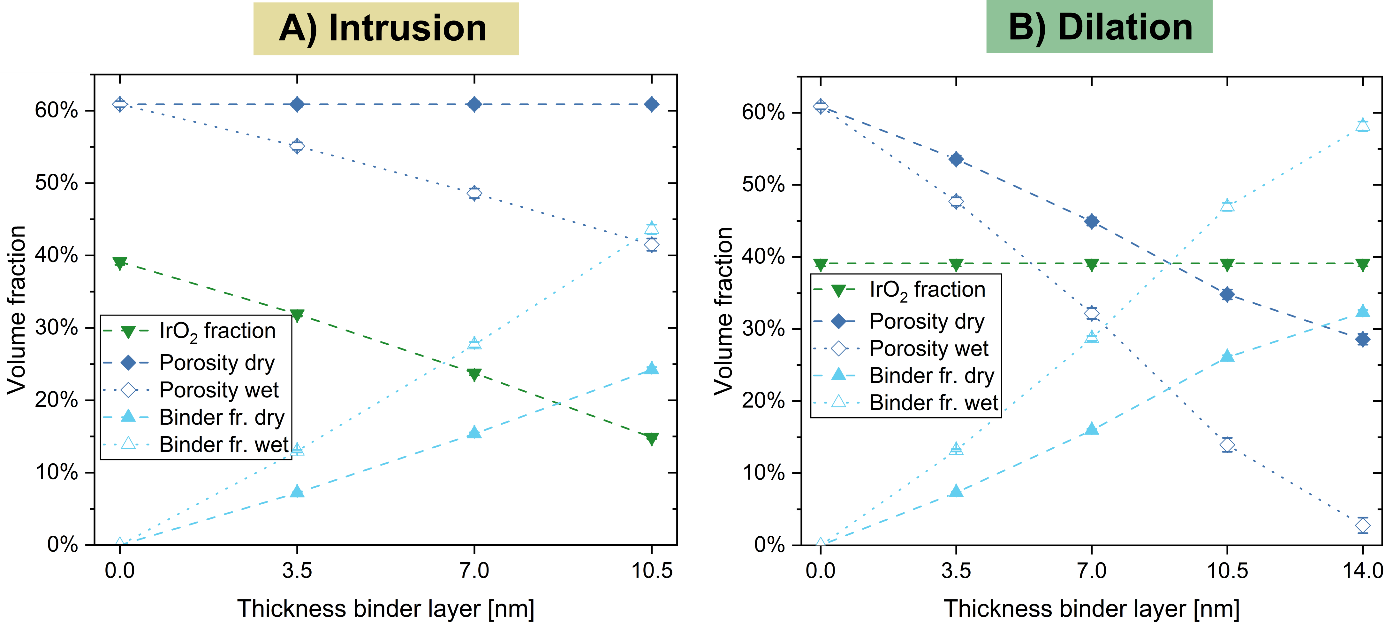


Figure 20 Comparison between the volume phase fraction under dry and wet conditions for the A) intrusion and B) dilation scenario. The binder volume increased by a factor of 1.8 under operating conditions (liquid water and 80 °C).


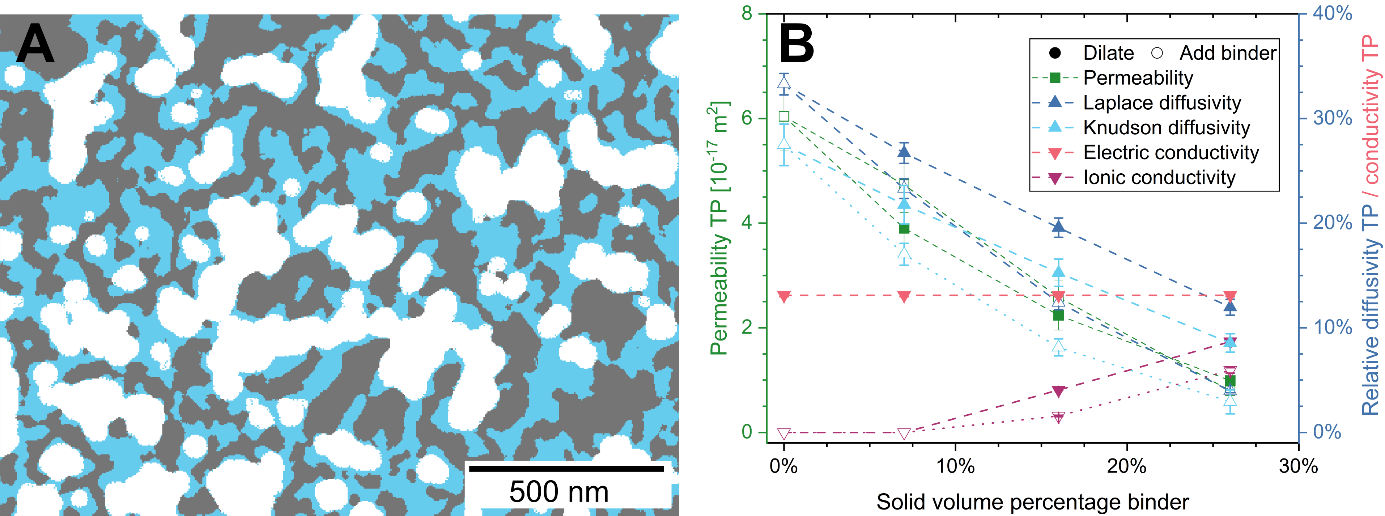


Figure 21 A) Visualization of binder phase (light blue) in CL modeled via the “Add Binder” function. B) Comparison of transport properties in TP direction between homogeneous coating with binder via dilation and “Add Binder” modeling of the binder. Solid volume percentages of the binder were chosen to match roughly the thickness of the binder of 0.0 nm (0 vox.), 3.5 nm (1 vox.), 7.0 nm (2 vox.), and 10.5 nm (3 vox.).


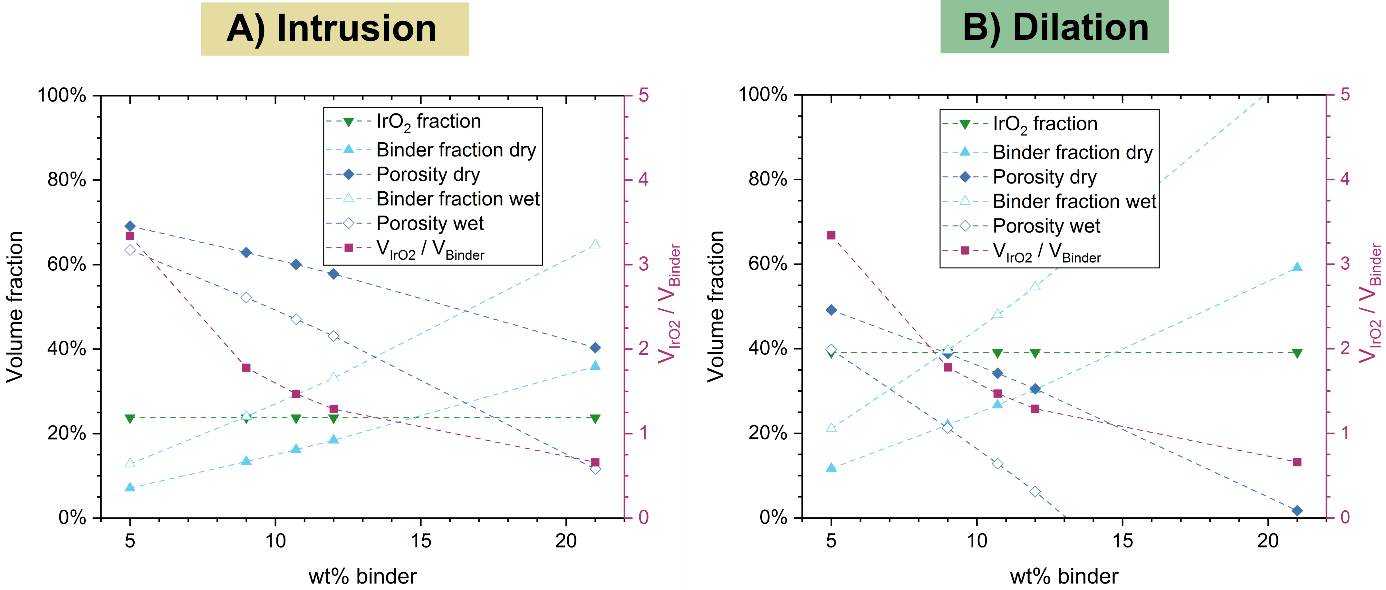


Figure 22 Determination of the volume fractions under dry and wet conditions dependent on the wt.% of the binder for the intrusion A) and dilation B) scenario. Explicitly depicted are the wt% binder investigated in the publication of Bühler et al.^[6]^

**Transport parameter catalyst layer**

***Knudsen diffusion CL***

Table 2 Relative diffusivity tensor of the CL of volume 1

| Relative Diffusivity Volume 1 [%] | X (In-plane) | Y (In-plane) | Z (Through-plane) |
| --- | --- | --- | --- |
| X (In-plane) | 29.33 | 0.23 | 2.08 |
| Y (In-plane) | 0.23 | 32.27 | 0.21 |
| Z (Through-plane) | 2.08 | 0.21 | 30.29 |

Table 3 Relative diffusivity tensor of the CL of volume 2

| Relative Diffusivity Volume 2 [%] | X (In-plane) | Y (In-plane) | Z (Through-plane) |
| --- | --- | --- | --- |
| X (In-plane) | 29.05 | 1.80 | 1.07 |
| Y (In-plane) | 1.80 | 31.76 | 0.76 |
| Z (Through-plane) | 1.07 | 0.76 | 26.33 |

Table 4 Relative diffusivity tensor of the CL of volume 3

| Relative Diffusivity Volume 3 [%] | X (In-plane) | Y (In-plane) | Z (Through-plane) |
| --- | --- | --- | --- |
| X (In-plane) | 28.80 | 1.09 | 0.28 |
| Y (In-plane) | 1.09 | 31.05 | 0.65 |
| Z (Through-plane) | 0.28 | 0.65 | 25.84 |

Table 5 Averaged relative diffusivity tensor of the CL

| Mean Relative Diffusivity [%] | X (In-plane) | Y (In-plane) | Z (Through-plane) |
| --- | --- | --- | --- |
| X (In-plane) | 29.01 | 1.11 | 0.97 |
| Y (In-plane) | 1.11 | 31.58 | 0.57 |
| Z (Through-plane) | 0.97 | 0.57 | 27.07 |

Table 6 Averaged effective diffusivity tensor of O_2_ in H_2_O in CL

| Mean Effective Diffusivity [%] | X (In-plane) | Y (In-plane) | Z (Through-plane) |
| --- | --- | --- | --- |
| X (In-plane) | 1.54E-05 | 5.90E-07 | 5.15E-07 |
| Y (In-plane) | 5.90E-07 | 1.68E-05 | 3.05E-07 |
| Z (Through-plane) | 5.15E-07 | 3.05E-07 | 1.44E-05 |

***Laplace diffusion CL***

Table 7 Relative diffusivity tensor of the CL of volume 1

| Relative Diffusivity Volume 1 [%] | X (In-plane) | Y (In-plane) | Z (Through-plane) |
| --- | --- | --- | --- |
| X (In-plane) | 34.53 | 0.50 | 1.05 |
| Y (In-plane) | 0.50 | 37.08 | -0.17 |
| Z (Through-plane) | 1.04 | -0.03 | 34.68 |

Table 8 Relative diffusivity tensor of the CL of volume 2

| Relative Diffusivity Volume 2 [%] | X (In-plane) | Y (In-plane) | Z (Through-plane) |
| --- | --- | --- | --- |
| X (In-plane) | 33.79 | 1.36 | 0.80 |
| Y (In-plane) | 1.33 | 36.80 | 0.25 |
| Z (Through-plane) | 0.75 | 0.35 | 32.91 |

Table 9 Relative diffusivity tensor of the CL of volume 3

| Relative Diffusivity Volume 3 [%] | X (In-plane) | Y (In-plane) | Z (Through-plane) |
| --- | --- | --- | --- |
| X (In-plane) | 33.70 | 1.06 | 0.42 |
| Y (In-plane) | 1.05 | 36.70 | 0.06 |
| Z (Through-plane) | 0.42 | 0.10 | 32.26 |

Table 10 Averaged relative diffusivity tensor of the CL

| Mean Relative Diffusivity [%] | X (In-plane) | Y (In-plane) | Z (Through-plane) |
| --- | --- | --- | --- |
| X (In-plane) | 33.93 | 1.02 | 0.69 |
| Y (In-plane) | 1.01 | 36.82 | 0.07 |
| Z (Through-plane) | 0.68 | 0.15 | 33.05 |

Table 11 Averaged effective diffusivity tensor of O_2_ in H_2_O in CL

| Mean Effective Diffusivity [cm^2^ s^-1^] | X (In-plane) | Y (In-plane) | Z (Through-plane) |
| --- | --- | --- | --- |
| X (In-plane) | 1.80E-05 | 5.41E-07 | 3.68E-07 |
| Y (In-plane) | 5.36E-07 | 1.96E-05 | 3.55E-08 |
| Z (Through-plane) | 3.59E-07 | 7.94E-08 | 1.76E-05 |

Table 12 Tortuosity factor κ and geometric tortuosity τ of the CL of volume 1

|  | Tortuosity factor *κ* | Geometric tortuosity τ |
| --- | --- | --- |
| X (In-plane) | 1.78 | 1.34 |
| Y (In-plane) | 1.66 | 1.29 |
| Z (Through-plane) | 1.78 | 1.33 |

Table 13 Tortuosity factor κ and geometric tortuosity τ of the CL of volume 2

|  | Tortuosity factor *κ* | Geometric tortuosity τ |
| --- | --- | --- |
| X (In-plane) | 1.79 | 1.34 |
| Y (In-plane) | 1.65 | 1.28 |
| Z (Through-plane) | 1.84 | 1.36 |

Table 14 Tortuosity factor κ and geometric tortuosity τ of the CL of volume 3

|  | Tortuosity factor *κ* | Geometric tortuosity τ |
| --- | --- | --- |
| X (In-plane) | 1.80 | 1.34 |
| Y (In-plane) | 1.65 | 1.29 |
| Z (Through-plane) | 1.88 | 1.37 |

Table 15 Averaged tortuosity factor κ and geometric tortuosity τ of the CL

|  | Tortuosity factor *κ* | Geometric tortuosity τ |
| --- | --- | --- |
| X (In-plane) | 1.79 | 1.34 |
| Y (In-plane) | 1.65 | 1.29 |
| Z (Through-plane) | 1.84 | 1.36 |

***Bosanquet approximation CL***

Table 16 Averaged effective diffusivity tensor of O_2_ in H_2_O in CL with the Bosanquet approximation

| Mean Effective Diffusivity [cm^2^ s^-1^] | X (In-plane) | Y (In-plane) | Z (Through-plane) |
| --- | --- | --- | --- |
| X (In-plane) | 8.30E-06 | 2.86E-07 | 2.29E-07 |
| Y (In-plane) | 2.85E-07 | 9.05E-06 | 9.75E-08 |
| Z (Through-plane) | 2.27E-07 | 1.07E-07 | 7.91E-06 |

***Flow permeability CL***

Table 17 Flow permeability tensor of the CL of volume 1

| Flow Permeability [m^2^] | X (In-plane) | Y (In-plane) | Z (Through-plane) |
| --- | --- | --- | --- |
| X (In-plane) | 6.49E-17 | 4.44E-21 | 9.94E-18 |
| Y (In-plane) | -1.58E-18 | 7.40E-17 | -1.40E-18 |
| Z (Through-plane) | 3.20E-18 | 4.15E-19 | 6.93E-17 |

Table 18 Flow permeability tensor of the CL of volume 2

| Flow Permeability [m^2^] | X (In-plane) | Y (In-plane) | Z (Through-plane) |
| --- | --- | --- | --- |
| X (In-plane) | 5.64E-17 | 5.99E-18 | 1.26E-17 |
| Y (In-plane) | 1.54E-18 | 6.65E-17 | 1.91E-18 |
| Z (Through-plane) | 3.89E-19 | 4.36E-19 | 5.76E-17 |

Table 19 Flow permeability tensor of the CL of volume 3

| Flow Permeability [m^2^] | X (In-plane) | Y (In-plane) | Z (Through-plane) |
| --- | --- | --- | --- |
| X (In-plane) | 5.64E-17 | 2.95E-18 | -7.41E-19 |
| Y (In-plane) | 1.44E-18 | 6.28E-17 | 2.82E-18 |
| Z (Through-plane) | 7.70E-20 | 3.19E-19 | 5.43E-17 |

Table 20 Averaged flow permeability tensor of the CL

| Flow Permeability [m^2^] | X (In-plane) | Y (In-plane) | Z (Through-plane) |
| --- | --- | --- | --- |
| X (In-plane) | 5.85E-17 | 3.22E-18 | 6.12E-18 |
| Y (In-plane) | 7.47E-19 | 6.67E-17 | 1.51E-18 |
| Z (Through-plane) | 9.30E-19 | 3.80E-19 | 5.90E-17 |

***Electrical conduction CL***

Table 21 Relative electrical conductivity tensor of the CL of volume 1

| Relative Conductivity [%] | X (In-plane) | Y (In-plane) | Z (Through-plane) |
| --- | --- | --- | --- |
| X (In-plane) | 11.87 | 0.02 | 0.58 |
| Y (In-plane) | -0.02 | 13.61 | 0.33 |
| Z (Through-plane) | 0.70 | 0.36 | 13.20 |

Table 22 Relative electrical conductivity tensor of the CL of volume 2

| Relative Conductivity [%] | X (In-plane) | Y (In-plane) | Z (Through-plane) |
| --- | --- | --- | --- |
| X (In-plane) | 13.50 | 0.76 | 0.51 |
| Y (In-plane) | 0.78 | 14.86 | 0.11 |
| Z (Through-plane) | 0.54 | 0.05 | 12.92 |

Table 23 Relative electrical conductivity tensor of the CL of volume 3

| Relative Conductivity [%] | X (In-plane) | Y (In-plane) | Z (Through-plane) |
| --- | --- | --- | --- |
| X (In-plane) | 13.68 | 0.33 | 0.26 |
| Y (In-plane) | 0.36 | 15.03 | 0.18 |
| Z (Through-plane) | 0.26 | 0.11 | 13.20 |

Table 24 Averaged relative electrical conductivity tensor of the CL

| Relative Conductivity [%] | X (In-plane) | Y (In-plane) | Z (Through-plane) |
| --- | --- | --- | --- |
| X (In-plane) | 13.18 | 0.39 | 0.42 |
| Y (In-plane) | 0.41 | 14.63 | 0.19 |
| Z (Through-plane) | 0.46 | 0.15 | 13.11 |

Table 25 Averaged effective electrical conductivity tensor of the CL

| Effective Conductivity [S/m] | X (In-plane) | Y (In-plane) | Z (Through-plane) |
| --- | --- | --- | --- |
| X (In-plane) | 3.41E+02 | 1.01E+01 | 1.09E+01 |
| Y (In-plane) | 1.06E+01 | 3.79E+02 | 4.92E+00 |
| Z (Through-plane) | 1.19E+01 | 3.89E+00 | 3.40E+02 |

***Thermal conduction CL***

Table 26 Thermal conductivity tensor of the CL of volume 1 filled with water or oxygen

| Thermal Conductivity [W m^-1^K^-1^] | X (In-plane) | Y (In-plane) | Z (Through-plane) |
| --- | --- | --- | --- |
| X (In-plane) | 1.67E+00 / 6.85E-01 | 6.45E-03 / 3.05E-03 | 2.16E-02 / 2.94E-02 |
| Y (In-plane) | 6.72E-03 / 1.78E-03 | 1.70E+00 / 7.58E-01 | 2.09E-02 / 1.89E-02 |
| Z (Through-plane) | 2.20E-02 / 3.34E-02 | 2.06E-02 / 1.93E-02 | 1.70E+00 / 7.43E-01 |

Table 27 Thermal conductivity tensor of the CL of volume 2 with water or oxygen

| Thermal Conductivity [W m^-1^K^-1^] | X (In-plane) | Y (In-plane) | Z (Through-plane) |
| --- | --- | --- | --- |
| X (In-plane) | 1.72E+00 / 7.56E-01 | 2.56E-02 / 3.81E-02 | 1.78E-02 / 2.58E-02 |
| Y (In-plane) | 2.54E-02 / 3.87E-02 | 1.75E+00 / 8.20E-01 | 2.74E-02 / 1.18E-02 |
| Z (Through-plane) | 1.77E-02 / 2.70E-02 | 2.67E-02 / 9.54E-03 | 1.72E+00 / 7.36E-01 |

Table 28 Thermal conductivity tensor of the CL of volume 3 with water or oxygen

| Thermal Conductivity [W m^-1^K^-1^] | X (In-plane) | Y (In-plane) | Z (Through-plane) |
| --- | --- | --- | --- |
| X (In-plane) | 1.72E+00 / 7.64E-01 | 1.68E-02 / 1.82E-02 | 1.07E-02 / 1.38E-02 |
| Y (In-plane) | 1.71E-02 / 1.97E-02 | 1.75E+00 / 8.28E-01 | 2.40E-02 / 1.45E-02 |
| Z (Through-plane) | 1.03E-02 / 1.34E-02 | 2.28E-02 / 1.08E-02 | 1.72E+00 / 7.48E-01 |

Table 29 Averaged thermal conductivity tensor of the CL with water or oxygen

| Thermal Conductivity [W m^-1^K^-1^] | X (In-plane) | Y (In-plane) | Z (Through-plane) |
| --- | --- | --- | --- |
| X (In-plane) | 1.71E+00 / 7.42E-01 | 1.71E-02 / 2.10E-02 | 1.56E-02 / 2.14E-02 |
| Y (In-plane) | 1.73E-02 / 2.15E-02 | 1.74E+00 / 8.09E-01 | 2.43E-02 / 1.47E-02 |
| Z (Through-plane) | 1.55E-02 / 2.26E-02 | 2.35E-02 / 1.25E-02 | 1.72E+00 / 7.43E-01 |

Table 30 Simulated averaged thermal conductivity values of the CL filled with water or oxygen, assuming different thermal conductivities for iridium oxide.

| Thermal Conductivity [W m^-1^K^-1^] | Water filled | Oxygen filled |
| --- | --- | --- |
| λ_IrOx_ = 1 W m^-1^K^-1^ | 0.79 | 0.20 |
| λ_IrOx_ = 5 W m^-1^K^-1^ | 1.72 | 0.76 |
| λ_IrOx_ = 10 W m^-1^K^-1^ | 2.60 | 1.45 |

***Literature comparison of the tortuosity of pore space and binder of CL***

Table 31 Comparison of the tortuosity of the pore space between the here reconstructed CL with (scenario “dilate” 3 voxels and scenario “intrude” 2 voxels) and without modeled binder, and CLs including a binder phase from literature.

| Tomography CL | Pore volume | IP tortuosity factor pore | TP tortuosity factor pore |
| --- | --- | --- | --- |
| IrOx without binder | 60.88 % | 1.73 | 1.88 |
| IrOx 3 vox. dilate | 34.89 % | 2.73 | 2.89 |
| IrO_x_ 2 vox. intrude | 60.88 % | 1.73 | 1.88 |
| IrRuOx^[7]^ | 29 % | - | 6.7 |
| IrO_2_ @ TiO_2_^[8]^ | 39 % | 2.3 | 2.8 |
| IrO_2_ @ TiO_2_^[9]^ | 45 % | 1.65 | 1.5 |

Table 32 Comparison of the tortuosity of the pore space between the here reconstructed CL with (scenario “dilate” 3 voxels and scenario “intrude” 2 voxels) and without modeled binder, and CLs including a binder phase from literature. Tortuosity factors from Weber et al. ^[9]^ were determined from the ionic conductivity of the CL.

| Tomography CL | Binder volume | IP tortuosity factor binder | TP tortuosity factor binder |
| --- | --- | --- | --- |
| IrOx without binder | 0 % | 0 | 0 |
| IrOx 3 vox. dilate | 26.08 % | 3.01 | 2.99 |
| IrO_x_ 2 vox. intrude | 15.37 % | 4.29 | 4.16 |
| IrRuOx^[7]^ | 26 % | - | 3.5 |
| IrO_2_ @ TiO_2_^[8]^ | 15 % (22 % ink) | 6.1 | 7.2 |
| IrO_2_ @ TiO_2_^[9]^ | 21 % (20 % ink) | 3.5 | 8.5 |

**Transport parameters PTL**

***Laplace Diffusion PTL***

Table 33 Relative diffusivity tensor of the PTL

| Relative Diffusivity [%] | X (In-plane) | Y (In-plane) | Z (Through-plane) |
| --- | --- | --- | --- |
| X (In-plane) | 20.19 | 0.25 | -0.04 |
| Y (In-plane) | 0.25 | 20.66 | -0.08 |
| Z (Through-plane) | -0.03 | -0.06 | 27.15 |

Table 34 Effective diffusivity tensor of O_2_ in H_2_O

| Effective Diffusivity[cm^2^ s^-1^] | X (In-plane) | Y (In-plane) | Z (Through-plane) |
| --- | --- | --- | --- |
| X (In-plane) | 1.07E-05 | 1.30E-07 | -2.25E-08 |
| Y (In-plane) | 1.30E-07 | 1.10E-05 | -4.08E-08 |
| Z (Through-plane) | -1.34E-08 | -3.39E-08 | 1.44E-05 |

Table 35 Tortuosity factor κ and geometric tortuosity τ of the PTL

|  | Tortuosity factor *κ* | Geometric tortuosity τ |
| --- | --- | --- |
| X (In-plane) | 2.52 | 1.59 |
| Y (In-plane) | 2.46 | 1.57 |
| Z (Through-plane) | 1.87 | 1.37 |

Table 36 Geometric tortuosity literature comparison

| Geometric Tortuosity τ | τ Micro-CT | τ literature ^[3]^ |
| --- | --- | --- |
| X (In-plane) | 1.59 | 1.6 |
| Y (In-plane) | 1.57 | 1.6 |
| Z (Through-plane) | 1.37 | 1.5 |

***Flow permeability PTL***

Table 37 Flow permeability tensor of the PTL

| Flow Permeability [m^2^] | X (In-plane) | Y (In-plane) | Z (Through-plane) |
| --- | --- | --- | --- |
| X (In-plane) | 4.53E-12 | 6.35E-14 | 6.32E-14 |
| Y (In-plane) | 1.31E-13 | 4.82E-12 | -1.85E-13 |
| Z (Through-plane) | 4.63E-15 | -4.24E-15 | 8.35E-12 |

Table 38 Flow permeability literature comparison

| Flow Permeability $\boldsymbol{\kappa}$ | $\boldsymbol{\kappa}\boldsymbol{10}^{\boldsymbol{-12}}$ Micro-CT | $\boldsymbol{\kappa}\boldsymbol{10}^{\boldsymbol{-12}}$ literature ^[3]^ |
| --- | --- | --- |
| X (In-plane) | 4.53 | 3.7 |
| Y (In-plane) | 4.82 | 3.3 |
| Z (Through-plane) | 8.35 | 5.0 |

Table 39 Reynolds number of flow simulation. For the calculation of the Reynolds number Re, the density of the fluid ρ, the flow speed v, the characteristic length L (here determined via the square root of the permeability), and the dynamic viscosity η are required. The density and dynamic viscosity values of water were taken from the GeoDict material data base: ρ_H2O@80°C,1atm_ = 917.8029 kg m^-3^ ^[10]^ and η_ρH2O@80°C,1atm_ = 0.000354355021 kg m^-1^ s^-1 [10]^.

|  | Reynolds number |
| --- | --- |
| X (In-plane) | 0.00710916 |
| Y (In-plane) | 0.00732856 |
| Z (Through-plane) | 0.00965039 |

***Electrical conduction PTL***

Table 40 Relative electrical conductivity tensor of the PTL

| Relative Conductivity [%] | X (In-plane) | Y (In-plane) | Z (Through-plane) |
| --- | --- | --- | --- |
| X (In-plane) | 28.20 | 0.36 | -0.04 |
| Y (In-plane) | 0.36 | 28.52 | -0.01 |
| Z (Through-plane) | -0.03 | -0.00 | 25.67 |

Table 41 Effective electrical conductivity tensor of the PTL

| Effective Conductivity [S/m] | X (In-plane) | Y (In-plane) | Z (Through-plane) |
| --- | --- | --- | --- |
| X (In-plane) | 6.71E+05 | 8.50E+03 | -1.02E+03 |
| Y (In-plane) | 8.64E+03 | 6.79E+05 | -1.46E+02 |
| Z (Through-plane) | -7.42E+02 | -4.19E+01 | 6.11E+05 |

Table 42 Electrical conductivity literature comparison

| Electrical Conductivity [S/m] | $\boldsymbol{\sigma}_{\boldsymbol{elec}} \boldsymbol{10}^{\boldsymbol{5}}$ | $\boldsymbol{\sigma}_{\boldsymbol{elec}} \boldsymbol{10}^{\boldsymbol{5}}$ literature ^[3]^ |
| --- | --- | --- |
| X (In-plane) | 6.71 | 5.9 |
| Y (In-plane) | 6.79 | 5.5 |
| Z (Through-plane) | 6.11 | 5.2 |

***Thermal conduction PTL***

Table 43 Thermal conductivity tensor of the PTL (completely with water flooded PTL)

| Thermal Conductivity [W m^-1^K^-1^] | X (In-plane) | Y (In-plane) | Z (Through-plane) |
| --- | --- | --- | --- |
| X (In-plane) | 7.04 | 0.07 | -0.01 |
| Y (In-plane) | 0.07 | 7.09 | -0.00 |
| Z (Through-plane) | -0.01 | -0.00 | 6.69 |

Table 44 Thermal conductivity tensor of the PTL (completely with oxygen invaded PTL)

| Thermal Conductivity [W m^-1^K^-1^] | X (In-plane) | Y (In-plane) | Z (Through-plane) |
| --- | --- | --- | --- |
| X (In-plane) | 6.22 | 0.08 | -0.01 |
| Y (In-plane) | 0.08 | 6.28 | -0.00 |
| Z (Through-plane) | -0.01 | -0.00 | 5.68 |

**Transport parameter PTE**

Table 45 Comparison of the permeability values of different cuboid volumes of the PTE. The first cuboid begins in the TP direction, where the SVP exceeds the value of 5% of the mean SVP of the bulk PTL, and ends where it undercuts this value. The second cuboid has the boundary value of the mean SVP of the bulk PTL. And the third cuboid is the bulk PTL.

| X / Y / Z | PTE 5% mean SVP | PTE mean SVP | PTL bulk |
| --- | --- | --- | --- |
| Permeability 10^-12^ [m^2^] | 6.02 / 6.20 / 8.66 | 4.66 / 4.95 / 8.39 | 4.53 / 4.82 / 8.35 |
| Relative diffusivity [%] | 21.53 / 21.87 / 27.00 | 19.85 / 20.35 / 26.55 | 20.19 / 20.66 / 27.15 |
| Rel. elect. conductivity [%] | 28.10 / 28.32 / 9.67 | 28.71 / 29.10 / 25.91 | 28.20 / 28.52 / 25.67 |

***Electrical conductivity PTE***

Table 46 Comparison between the electrical conductivity of the titanium fiber PTL with a non-conductive void and the electrical conductivity of the titanium fiber with the whole porous space filled with catalyst layer.

| Electrical Conductivity [S/m] | $\boldsymbol{\sigma}_{\boldsymbol{elec, pure Ti}}$ | $\boldsymbol{\sigma}_{\boldsymbol{elec, Ti+pore as CL}}$ | *Abs. change*$\boldsymbol{\sigma}_{\boldsymbol{elec}}$ | *Rel. change*$\boldsymbol{\sigma}_{\boldsymbol{elec}}$ |
| --- | --- | --- | --- | --- |
| X (In-plane) | 6.71276E+05 | 6.71906E+05 | 6.29E+02 | 0.094% |
| Y (In-plane) | 6.78819E+05 | 6.79414E+05 | 5.95E+02 | 0.088% |
| Z (Through-plane) | 6.10963E+05 | 6.11652E+05 | 6.89E+02 | 0.113% |

***Permeability and diffusivity PTE***


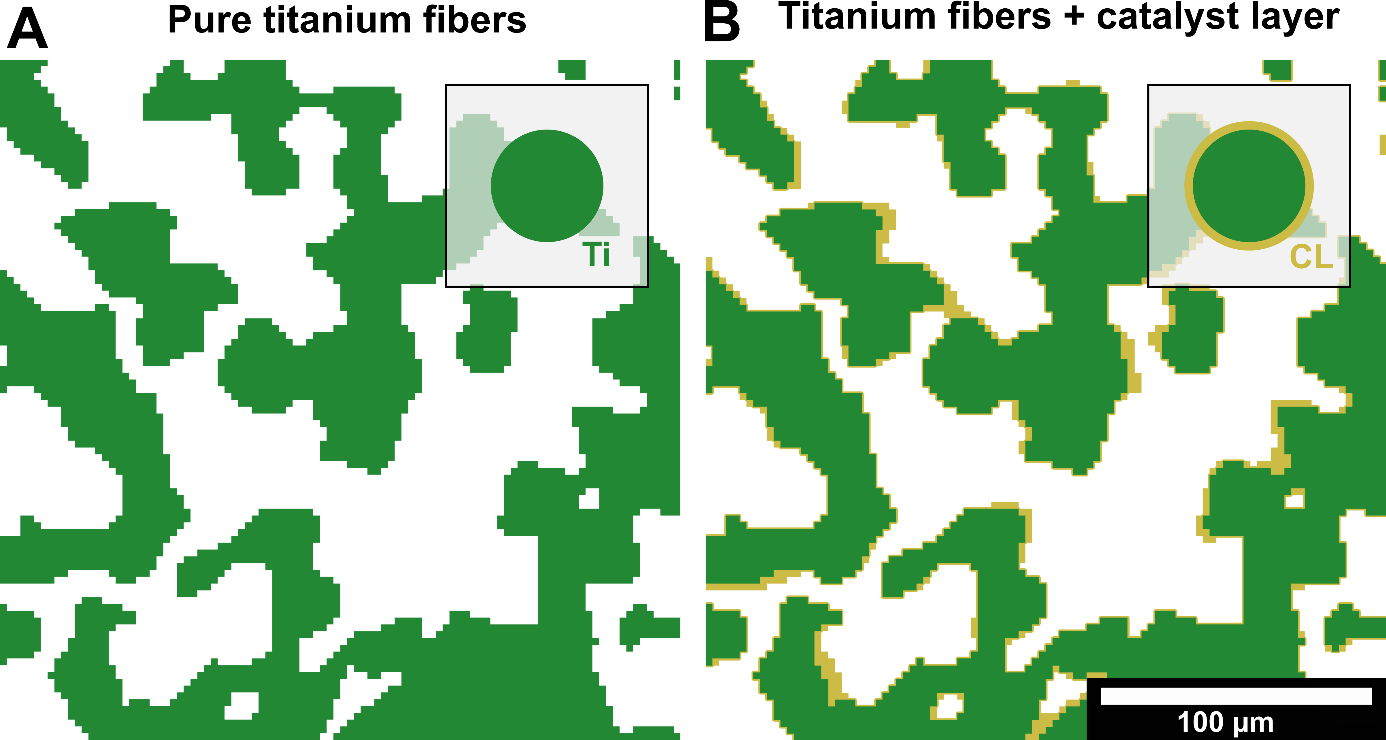


Figure 23 Illustration of the modeling of the catalyst layer around the titanium fibers using a morphological dilation operation. Note that the catalyst layer was modelled on the 3D dataset, and B) only shows a single 2D slice. Therefore, the catalyst layer does not show a uniform coating thickness on all surfaces.


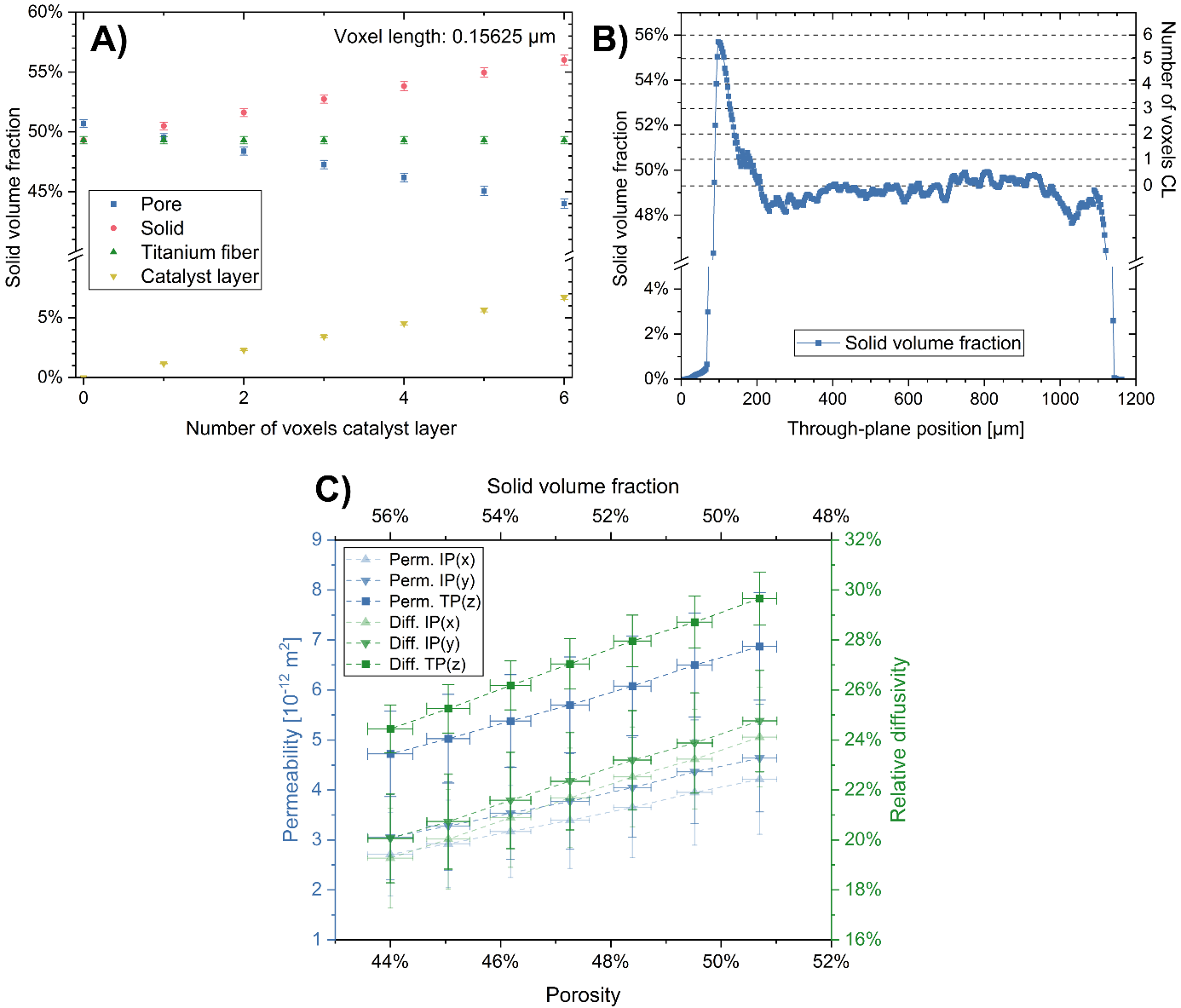


Figure 24 Modeling the CL around the titanium fibers by dilating the structure of the fiber from 0 to 6 voxels. A) With increasing CL thickness or CL voxels, the porosity decreases, and the CL volume fraction increases. B) The catalyst layer solid volume fraction was matched up with the solid volume trend of the PTE, taken from our previous publication.^[1]^ C) Change of permeability and diffusivity with increasing solid volume fraction and decreasing porosity due to the increasing amount in CL volume fraction.

***Outlook permeability PTE for future PTLs with thinner fibers***

Table 47 Calculated permeability values of the modelled PTEs with a solid and porous CL thickness of 6 voxel of a size of 0.15625 µm, equals a total thickness of around 1 µm. The permeability values were simulated for the original size fibers, the half size fibers and the quarter size fibers. The relative difference between the permeability values was calculated as (k_porous_-k_solid_) / k_porous_. Additionally, the permeability values for the titanium fiber PTL without any coating were determined.

| Permeability | Solid CL perm. | Porous CL perm. | Rel. diff. perm. | PTL ref. perm. |
| --- | --- | --- | --- | --- |
| **Original** | [10^-12^ m^2^] | [10^-12^ m^2^] |  | [10^-12^ m^2^] |
| IP (x) | 2.71 ± 0.84 | 2.76 ± 0.80 | 1.82 % | 4.21 ± 1.10 |
| IP (y) | 3.05 ± 0.85 | 3.11 ± 0.82 | 1.75 % | 4.64 ± 1.07 |
| TP (z) | 4.72 ± 0.76 | 4.79 ± 0.72 | 1.53 % | 6.87 ± 0.91 |
| **Half** | [10^-13^ m^2^] | [10^-13^ m^2^] |  | [10^-13^ m^2^] |
| IP (x) | 4.56 ± 0.64 | 4.71 ± 0.65 | 3.16 % | 10.62 ± 0.97 |
| IP (y) | 4.76 ± 0.59 | 4.92 ± 0.61 | 3.19 % | 11.04 ± 1.15 |
| TP (z) | 7.22 ± 0.46 | 7.44 ± 0.47 | 3.00 % | 16.16 ± 0.75 |
| **Quarter** | [10^-14^ m^2^] | [10^-14^ m^2^] |  | [10^-14^ m^2^] |
| IP (x) | 4.68 ± 1.02 | 4.97 ± 1.05 | 5.96 % | 27.22 ± 2.47 |
| IP (y) | 4.79 ± 0.74 | 5.10 ± 0.78 | 6.06 % | 28.74 ± 2.92 |
| TP (z) | 7.56 ± 0.73 | 8.03 ± 0.75 | 5.80 % | 41.41 ± 1.91 |


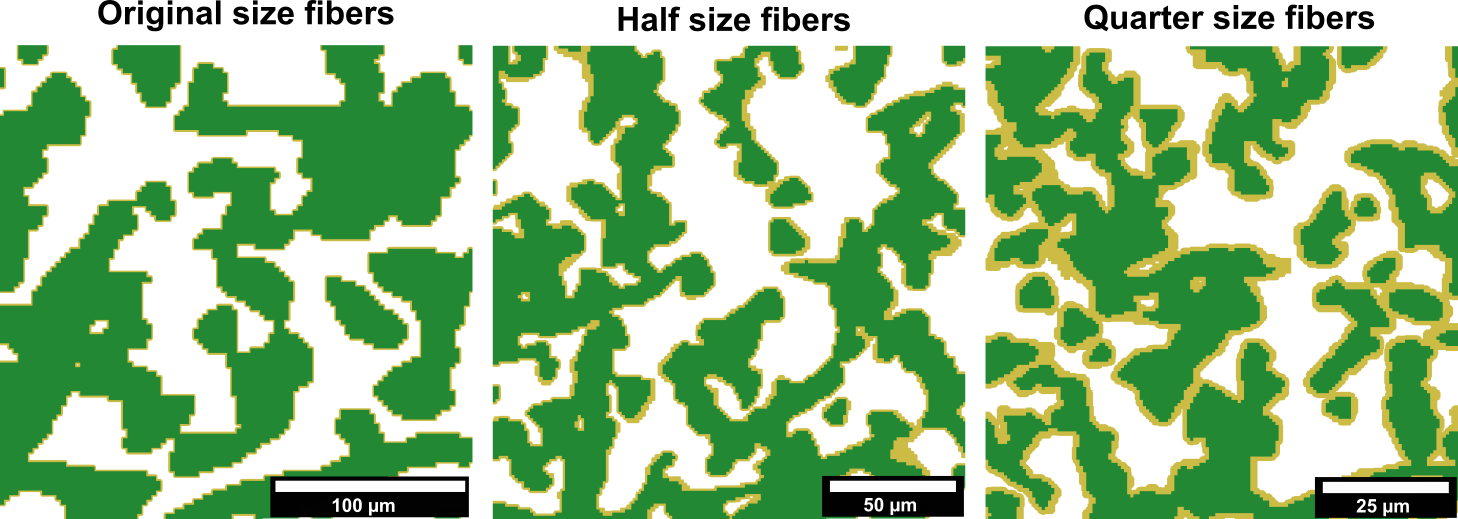


Figure 25 Images of different thick titanium fibers coated with catalyst. The catalyst layer thickness is around 1 µm for all three of them.

Table 48 Solid volume percentages (SVP) of the PTL, CL, and the void of the three modeled scenarios of the PTE.

|  | SVP PTL | SVP CL | Porosity |
| --- | --- | --- | --- |
| *Original size fibers* | 49.30 % ± 0.31 % | 6.70 % ± 0.19 % | 44.00 % ± 0.41 % |
| *Half size fibers* | 49.30 % ± 0.27 % | 12.41 % ± 0.18 % | 38.28 % ± 0.25 % |
| *Quarter size fibers* | 49.30 % ± 0.32 % | 21.85 % ± 0.30 % | 28.85 % ± 0.32 % |

**Depositions for FIB-SEMt**


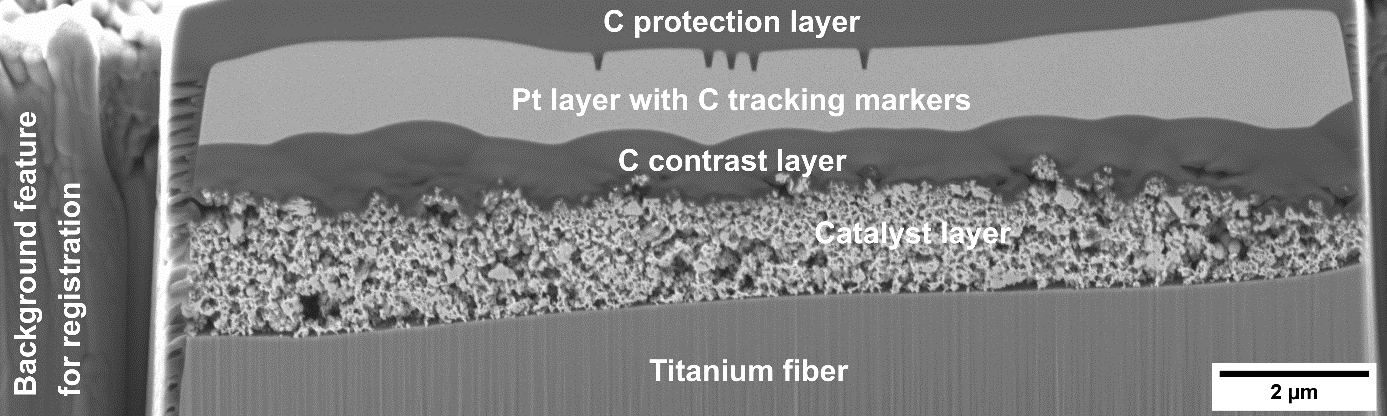


Figure 26 Single image to illustrate the different layers: Titanium fiber, catalyst layer, Carbon contrast layer, Pt layer with Carbon tracking markers, and Carbon protection layer. Additionally, a part of the background is visible as having features for the registration process.


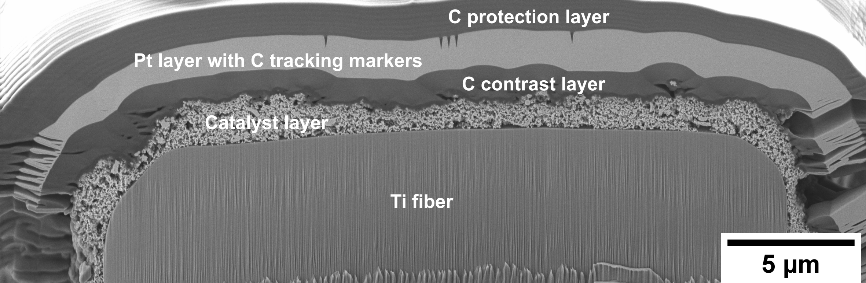


Figure 27 Illustration of the applied deposition steps for the fiber-catalyst layer tomography. First, a thin Carbon layer was deposited via an electron beam on top of the catalyst layer to prevent any damage occurring due to later ion-beam deposition. Afterward, an additional Carbon layer deposition via an ion beam was performed to obtain a good contrast layer. Further, a Platinum layer via an ion-beam deposition was deposited, including the milled tracking markers filled with Carbon. Finally, a protective carbon layer was applied to the fiber via ion-beam deposition.

**FIB and imaging parameters for CL serial sections**

Table 49 Parameters and settings for the preparation and imaging of the FIB-SEMt.

|  | CL | Catalyst-coated fiber |
| --- | --- | --- |
| Deposition of C contrast layer (e-beam) | 2 kV : 6 nA | 2 kV : 6 nA |
| Deposition of C contrast layer (ion-beam) | 30 kV : 300 pA | 30 kV : 300 pA |
| Deposition of Pt protection layer (e-beam) | 2 kV : 6 nA | - |
| Deposition of Pt protection layer (ion-beam) | 30 kV : 700 pA | 30 kV : 1.5 nA |
| Coarse cross section and trenches | 30 kV : 7 nA | 30 kV : 7 nA |
| Auto-tune and tracking markers | 30 kV : 50 pA | 30 kV : 50 pA |
| C highlighting of markers (ion-beam) | 30 kV : 50 pA | 30 kV : 50 pA |
| Deposition of C protection layer (ion-beam) | 30 kV : 700 pA | 30 kV : 700 pA |
| Fine cross section / Polishing | 30 kV : 700 pA / 100 pA | 30 kV : 1.5 nA / 300 pA |
| Serial section | 30 kV: 50 pA | 30 kV : 100 pA |
| Imaging conditions (SE & InLens) | 3 kV : 750 pA | 3 kV : 750 pA |

**Segmentation CL and catalyst-coated fiber**


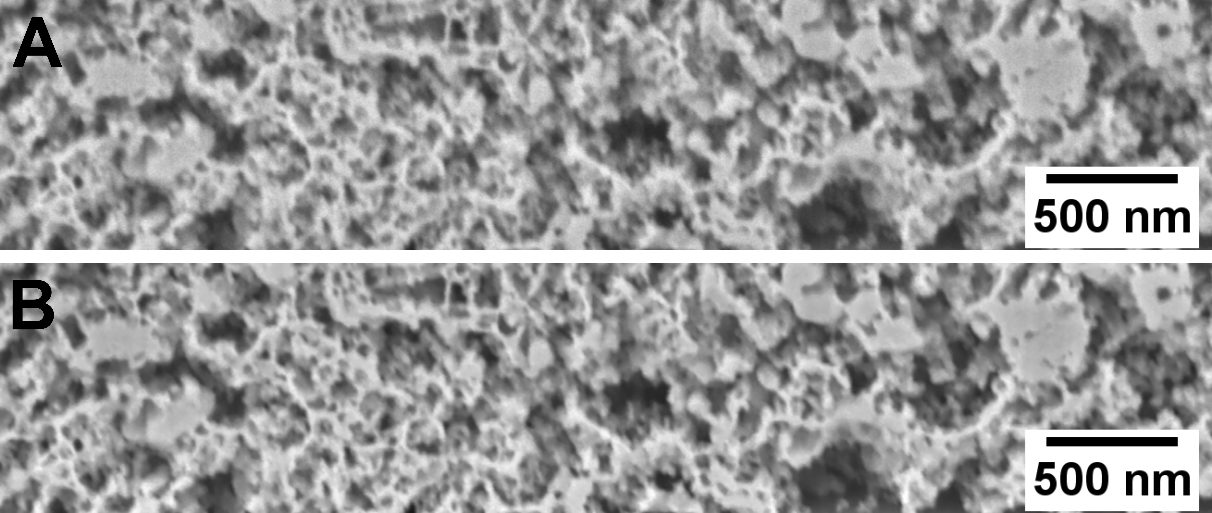


Figure 28 Difference between raw A) and processed B) image for segmentation. A non local-mean filter denoised the 3D dataset, and a sharpening filter improved the visibility of the boundaries between pore and grain.


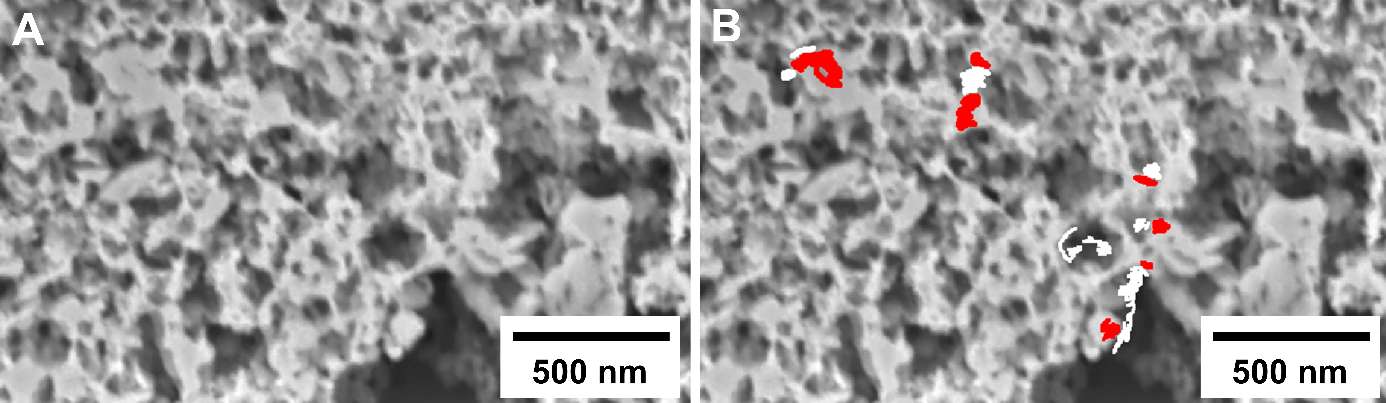


Figure 29 Illustration of raw image slice A) and labeling process B) of grain (red) and pore (white).


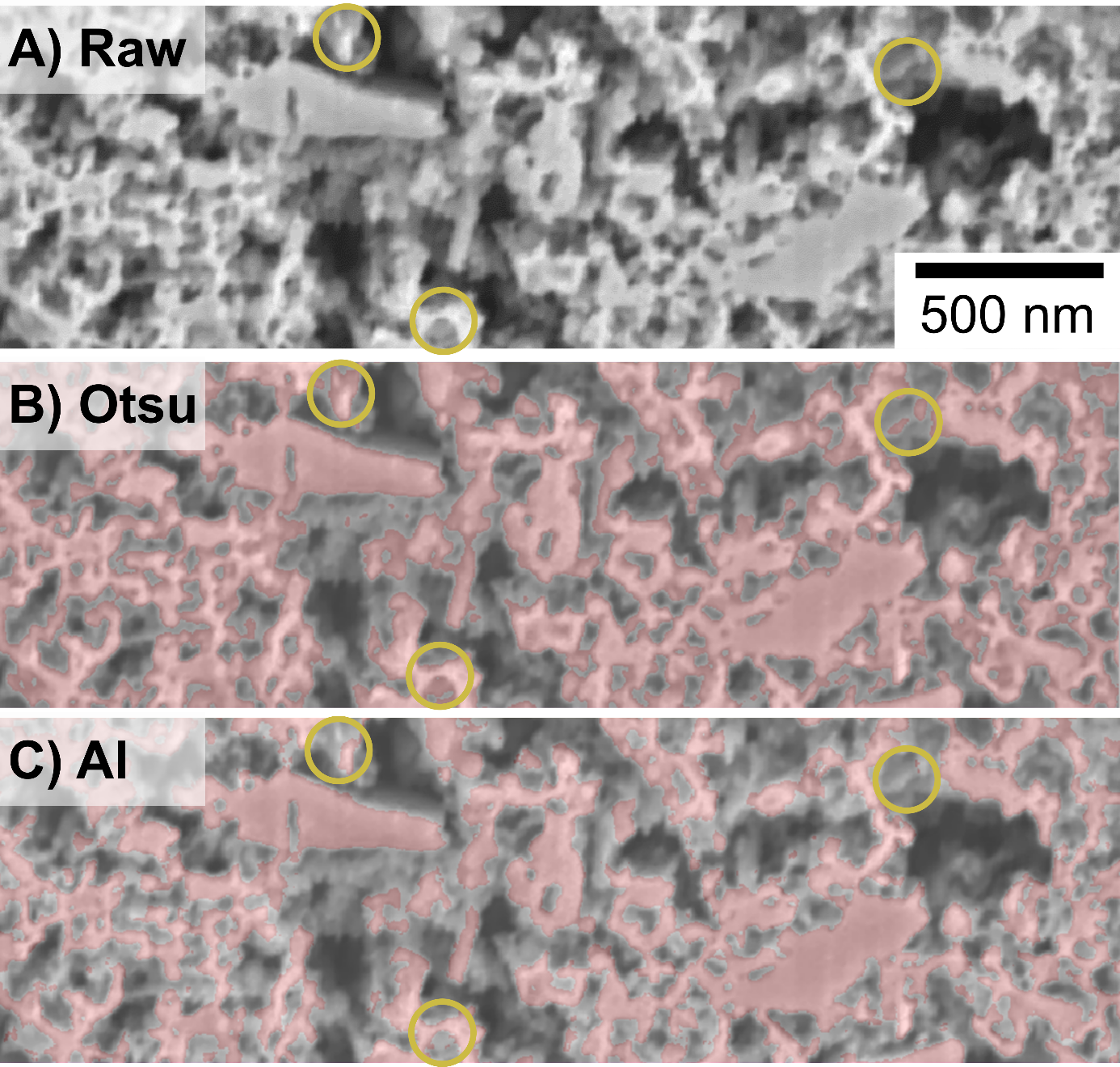


Figure 30 Illustration of the segmentation of the grey scale raw data A) with Otsu thresholding B) and the AI Unet3D algorithm C). Compared to the AI segmentation, the Otsu algorithm overestimates the grain area and falsely identifies background features as grain (see yellow circles). This would lead to an overestimation of the solid volume fraction (e.g., for volume 2: Otsu volume fraction 43.87%, and AI volume fraction 39.36%).


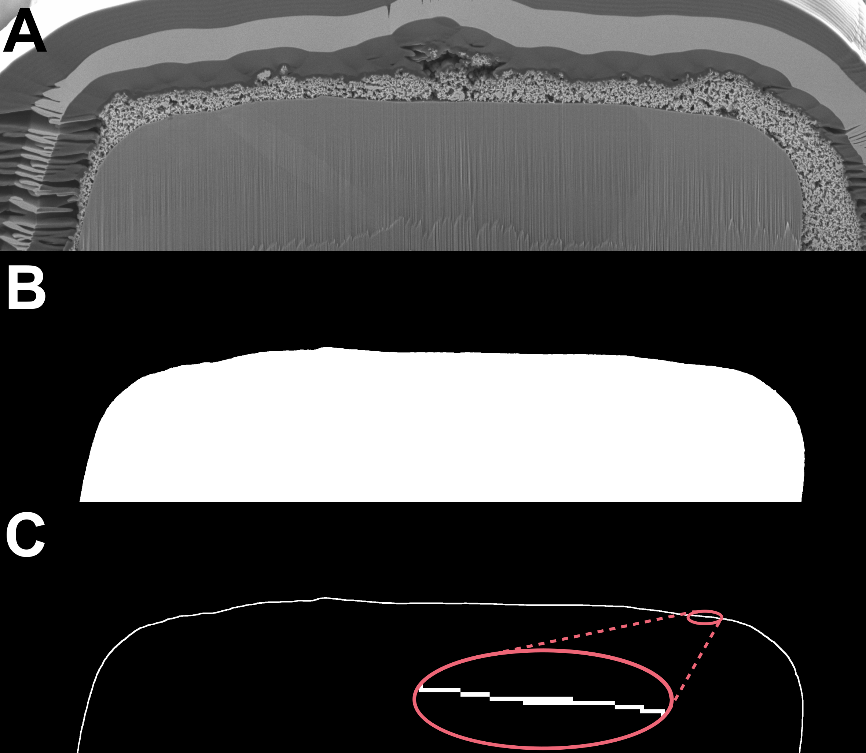


Figure 31 Illustration of edge detection of the fiber catalyst layer interface. The raw image (A) was segmented, and the segmented fiber part (B) was selected. Afterward, a canny edge algorithm was applied to obtain the edge between the fiber and catalyst layer (C). In (C), a zoomed-in view of the segmented line is highlighted. Therefore, it becomes visible that some parts of the edge are represented with two pixels. Our algorithm only considers the upper pixel to ensure that the later catalyst layer thickness analysis is not distorted by a double calculation of the thickness at the same location.


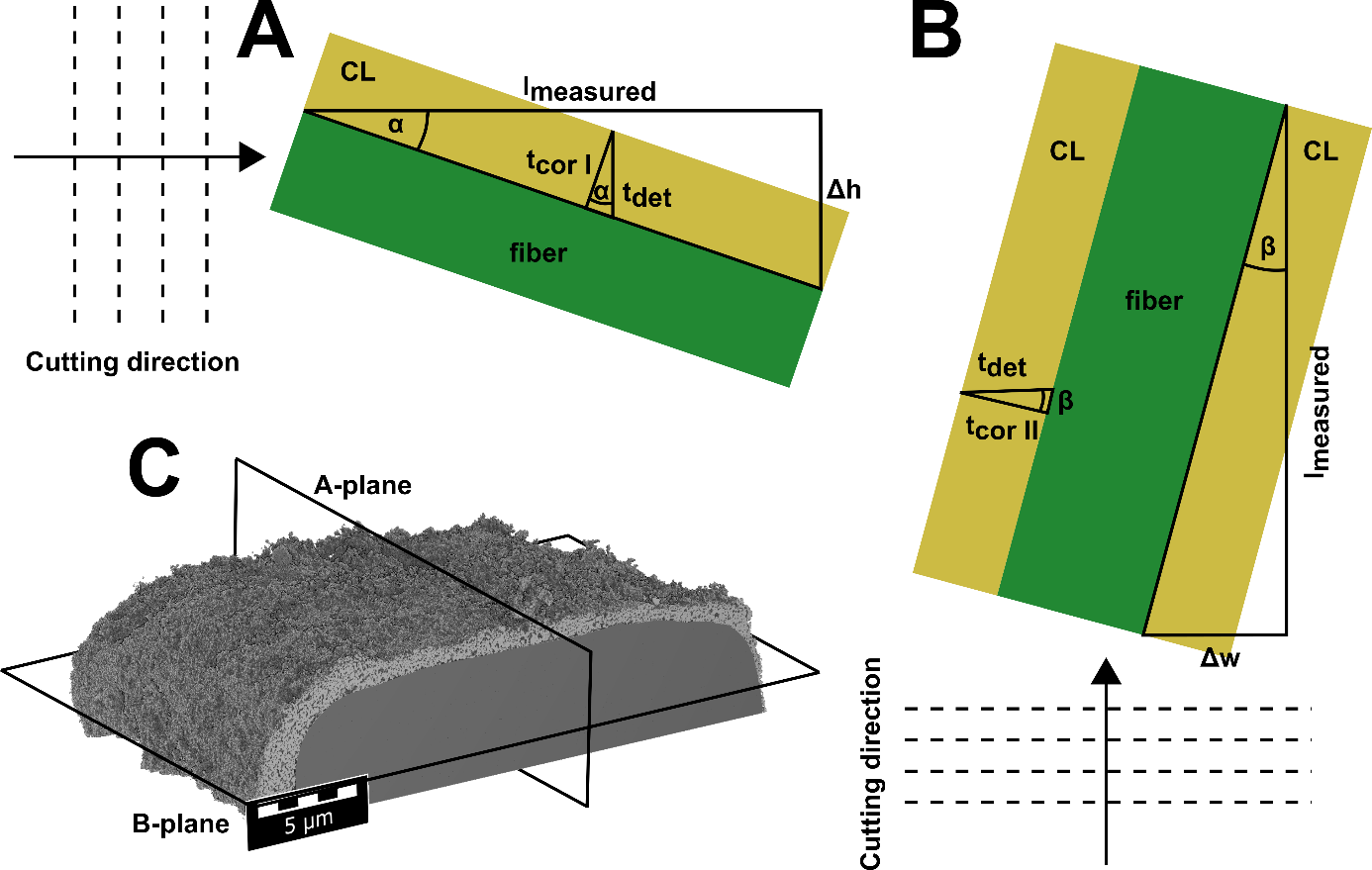


Figure 32 Illustration of the two possible geometric reasons for overestimating the determined catalyst layer thickness. A) The fiber is not completely horizontally aligned. This tilt leads to a thicker CL. B) The fiber and the FIB-cutting line are not aligned parallel due to a misalignment or a curved fiber. C) Illustration of the viewing planes of cases A and B. Normally, the distortion is a mixture of cases A and B. Only case B or A is valid if the normal's slope is infinity or zero.

**Possible geometric catalyst layer thickness errors**

Figure S32 illustrates two possible geometric reasons for the overestimation of the determined catalyst layer thickness. The fiber can have a slight tilt (Figure S32A) or the cutting direction is not completely parallel to the fiber (Figure S30B). The fiber ascended slightly (Figure S15), which leads to a height difference of Δ*h* =0.481 µm. Additionally, the fiber is lightly twisted in the right direction, which summed up to a shift of Δ*w* =1.490 µm (Figure S32). Knowing the total length of the reconstructed fiber volume of *l*_measured_ = 20.8 µm, the possible errors can be estimated according to the following geometric relationships (Figure S32):

| $t_{cor I}= t_{\det}\cos\alpha= t_{\det} \cos\left( \arctan\left( \frac{\Delta h}{l_{\mathrm{measured}}} \right) \right)$= $t_{\det}$*0.99973 | (9) |
| --- | --- |
| $t_{cor II}= t_{\det}\cos\beta= t_{\det} \cos\left( \arctan\left( \frac{\Delta w}{l_{\mathrm{measured}}} \right) \right)=t_{\det}$*0.99744 | (10) |
| $t_{\mathrm{cor}}> t_{\det}\cos\alpha\cos\beta=t_{\det}$*0.99717 | (11) |

This geometric estimation shows that the possible error of less than 0.3% is negligible.


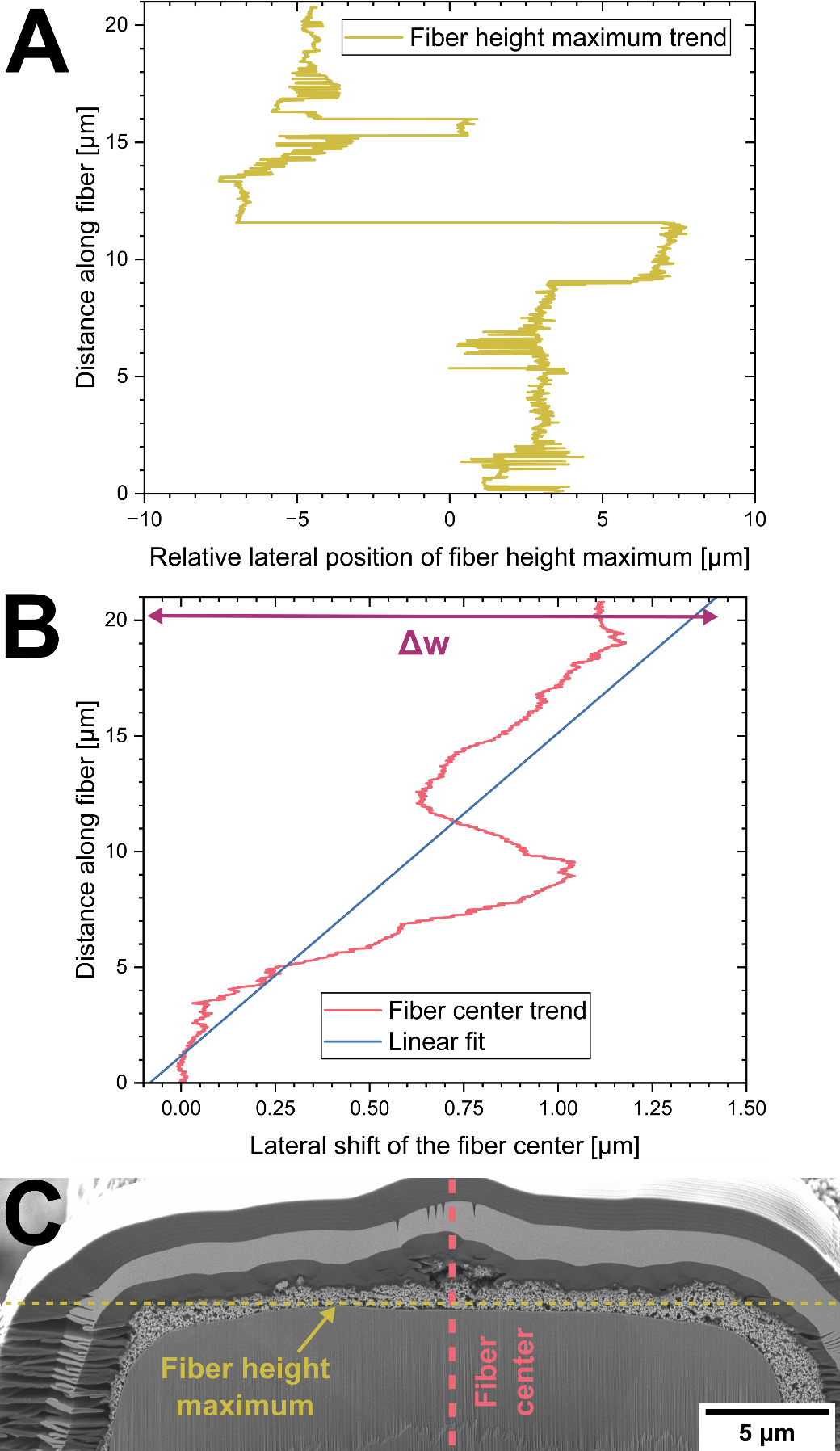


Figure 33 A) Trend of the lateral position of the highest fiber pixel in relation to fiber center. The highest fiber point jumps from the right to the left side after 10 µm. B) The vertical center line moves further right in the imaging window with increasing imaging time. Δw indicates the lateral shift of the fiber center based on linear interpolated trend of the data. C) Illustration and explanation of the above depicted parameters.

**Theoretical background of the simulation of transport parameters**

This chapter provides a comprehensive overview of the physical equation employed in simulations and the calculation of transport parameters performed in this work. All transport simulations were conducted using the GeoDict software from Math2Market. The mathematical and physical descriptions of the respective transport simulations were obtained from the respective GeoDict user guides^[11]^ (FlowDict^[12]^, DiffuDict^[13]^, and ConductoDict^[14]^). As stated in the main part of the publication, symmetric boundary conditions were implemented in the computation direction, while periodic boundary conditions were employed in the corresponding tangential directions. The material constants for diffusion, electric and ionic conductivity and thermal conductivity can be found also in the main part of our manuscript.

***Flow simulation and permeability (FlowDict^[12]^)***

The Stokes equation, a simplification of the Navier-Stokes equation, was used to simulate the flow in the PTL and CL because laminar flow was assumed due to the low Reynolds number *Re* ($Re\ll1$, see Equation S13 and Table S39):

| $-\mu\Delta\boldsymbol{u}+\nabla p=\boldsymbol{f}$ | (12) |
| --- | --- |
| $\nabla\boldsymbol{u}=0$ | (13) |
| $\boldsymbol{u}\vert_{\partial\Omega}=0$ (no-slip boundary conditions) | (14) |
|  |  |

Where *μ* is the fluid viscosity, ***u*** the fluid velocity, *p* the fluid pressure, and ***f*** external forces, like gravity and buoyancy. The permeability tensor ***K*** is calculated from Darcy’s law, which can be derived by the Stokes equation:^[15]^

| $\boldsymbol{v}^{i}=-\mu^{-1}\boldsymbol{K}\nabla p^{i}$ | | (15) | |
| --- | --- | --- | --- |
|  |  | |  |

Where ***v****^i^* is the averaged velocity vector occurring due to a pressure gradient $\nabla p^{i}$ in the *i*^th^ direction. Here, a flow rate of 40 ml min^-1^ in a flow area of 5 cm^-2^ and no external forces were applied for the simulation. For the calculation of the Reynolds number *Re*

| $Re=\frac{\rho vL}{\eta}$ | (16) |
| --- | --- |

the density of the fluid *ρ*, the flow speed *v*, the characteristic length L (here determined via the square root of the permeability), and the dynamic viscosity *η* are required. The density and dynamic viscosity values of water were taken from the GeoDict material data base: *ρ*_H2O@80°C,1atm_ = 917.80 kg m^-3^ and *η*_ρH2O@80°C,1atm_ *=* 3.54E-4 kg m^-1^ s^-1^.

***Diffusion (DiffuDict^[13]^)***

The diffusivity of the individual components was calculated depending on the Knudsen number Kn:

| $Kn=\frac{\lambda}{L}=\frac{length of mean free path}{representative physical length scale}$ | (17) |
| --- | --- |

Bulk or Laplace diffusion is present at small Knudsen numbers (Kn ≪ 1), where intermolecular collisions dominate the diffusion process. By contrast, high Knudsen numbers (Kn ≫ 1) indicate Knudsen diffusion, where collisions mainly happen through molecule-wall collisions.

Knudsen diffusion is the dominant mechanism in porous structures which have average pores sizes between 0 and 50 nm.^[16,17]^ The here investigated catalyst layer has pore sizes of around 80 nm, leading to a Kn ≅ 1 assuming an ideal gas with a mean free path of 68 nm for gas molecules for ambient conditions. In this intermediate regime (Kn ≅ 1), the Bosanquet approximation^[18]^ can be used to calculate the effective diffusion coefficient as the sum of two parallel diffusion resistances, the Knudsen and Laplace diffusion:

| $D_{\mathrm{Bosanquet}}=\left( D_{\mathrm{Knudsen}}^{-1}+D_{\mathrm{Laplace}}^{-1} \right)^{-1}$ | (18) |
| --- | --- |

Thus, bulk or Laplace diffusion is the dominant diffusion process in the anodic PTL. The bulk diffusion is expressed by Fick’s first law:

| $\boldsymbol{j}=-\boldsymbol{D}_{\mathrm{rel}}\nabla C$ | (19) |
| --- | --- |
| $\Delta C=0$ | (20) |

Where ***j*** is the diffusion flux, ***D***_rel_ is the relative diffusivity, and *C* is the concentration. A concentration of 1 at the inlet and of 0 at the outlet is assumed. For the bulk diffusion, the effective diffusivity ***D***_eff_ is calculated with the species-dependent part *D*_0_:

| $\boldsymbol{D}_{\mathrm{eff}}=D_{0}\boldsymbol{D}_{\mathrm{rel}}$ | (21) |
| --- | --- |
|  |  |

***Electrical and ionic conductivity (ConductoDict^[14]^)***

The electrical conduction is caused by a potential gradient $\nabla\varphi$ and is described by Ohm’s law and the Poisson equation:

| $\boldsymbol{j}=-\sigma_{\mathrm{rel}}\nabla\varphi$ | (22) | |  |
| --- | --- | --- | --- |
| $\nabla\left( \sigma_{l\mathrm{ocal}}\nabla\varphi\right)=0$ | | (23) | |

Where ***j*** is the current density, *σ*_rel_ the relative electrical conductivity, and *σ*_c_ the local electrical conductivity. A voltage difference of 1 V, as an arbitrary value, in the respective calculation direction is assumed to solve the above differential equation. Only considering a single conductive material in the porous system (IrO_x_ for the CL and Ti for the PTL), the effective electrical conductivity *σ*_eff_ is defined as:

| $\boldsymbol{\sigma}_{\mathrm{eff}}=\sigma_{0}\boldsymbol{\sigma}_{\mathrm{rel}}$ | (24) |
| --- | --- |

Where *σ*_0_ is the electrical bulk conductivity.

The ionic conductivity was calculated based on the same equations.

***Thermal conductivity (ConductoDict^[14]^)***

The thermal conductivity was solved with Fourier’s law, which is based on a temperature gradient $\nabla T$:

| $\dot{\boldsymbol{q}}=-k\nabla T$ | (25) | |  |
| --- | --- | --- | --- |
| $\nabla\left( k_{local}\nabla T \right)=0$ | | (26) | |

Where $\dot{\boldsymbol{q}}$ is the heat flux, *k* the effective thermal conductivity, and *k*_local_ the local thermal conductivity. A temperature drop from 81°C to 79 °C, as an arbitrary temperature difference, and no thermal conduction resistance were assumed.

[1] M. Bierling, D. McLaughlin, B. Mayerhöfer, S. Thiele, Toward Understanding Catalyst Layer Deposition Processes and Distribution in Anodic Porous Transport Electrodes in Proton Exchange Membrane Water Electrolyzers, *Advanced Energy Materials* **2023**, *13*, 2203636.

[2] D. McLaughlin, M. Bierling, B. Mayerhöfer, G. Schmid, S. Thiele, Digital Twin of a Hierarchical CO 2 Electrolyzer Gas Diffusion Electrode, *Adv Funct Materials* **2023**, *33*, 2212462.

[3] T. Schuler, R. de Bruycker, T. J. Schmidt, F. N. Büchi, Polymer Electrolyte Water Electrolysis: Correlating Porous Transport Layer Structural Properties and Performance: Part I. Tomographic Analysis of Morphology and Topology, *J. Electrochem. Soc.* **2019**, *166*, F270.

[4] L. G. Melo, A. P. Hitchcock, Electron beam damage of perfluorosulfonic acid studied by soft X-ray spectromicroscopy, *Micron* **2019**, *121*, 8.

[5] S. Yakovlev, N. P. Balsara, K. H. Downing, Insights on the study of nafion nanoscale morphology by transmission electron microscopy, *Membranes* **2013**, *3*, 424.

[6] M. Bühler, F. Hegge, P. Holzapfel, M. Bierling, M. Suermann, S. Vierrath, S. Thiele, Optimization of anodic porous transport electrodes for proton exchange membrane water electrolyzers, *Journal of Materials Chemistry A* **2019**, *7*, 26984.

[7] F. Hegge, R. Moroni, P. Trinke, B. Bensmann, R. Hanke-Rauschenbach, S. Thiele, S. Vierrath, Three-dimensional microstructure analysis of a polymer electrolyte membrane water electrolyzer anode, *J. Power Sources* **2018**, *393*, 62.

[8] S. de Angelis, T. Schuler, M. Sabharwal, M. Holler, M. Guizar-Sicairos, E. Müller, F. N. Büchi, Understanding the microstructure of a core–shell anode catalyst layer for polymer electrolyte water electrolysis, *Sci Rep* **2023**, *13*, 4280.

[9] C. C. Weber, S. de Angelis, R. Meinert, C. Appel, M. Holler, M. Guizar-Sicairos, L. Gubler, F. N. Büchi, Microporous transport layers facilitating low iridium loadings in polymer electrolyte water electrolysis, *EES Catalysis* **2024**, *2*, 585.

[10] CalcDampf, https://www.peacesoftware.de/einigewerte/wasser_dampf.html, accessed: September 2022.

[11] Math2Market GmbH, *GeoDict User Guide*, Math2Market GmbH **2023**.

[12] J. Hilden, S. Linden, B. Planas, *GeoDict 2023 User Guide: FlowDict Handbook*, Math2Market GmbH, Germany **2023**.

[13] J. Becker, A. Weber, B. Planas, *GeoDict 2023 User Guide: DiffuDict Handbook*, Math2Market GmbH, Germany **2022**.

[14] J. Becker, L. Chen, B. Planas, *GeoDict 2023 User Guide: ConductoDict Handbook*, Math2Market, Germany **2022**.

[15] S. Whitaker, Flow in porous media I: A theoretical derivation of Darcy's law, *Transport in Porous Media* **1986**, *1*, 3.

[16] K. Malek, M.-O. Coppens, Knudsen self- and Fickian diffusion in rough nanoporous media, *J. Chem. Phys.* **2003**, *119*, 2801.

[17] Y. Zeng, M. Luo, C. Qin, C. Liu, B. Chen, Investigation on the performance of proton exchange membrane water electrolyzer coupled with a catalyst layer pore network model, *Energy Conversion and Management: X* **2024**, *21*, 100523.

[18] W. G. Pollard, R. D. Present, On Gaseous Self-Diffusion in Long Capillary Tubes, *Phys. Rev.* **1948**, *73*, 762.
